# Characterising a Microsatellite for DRB Typing in *Aotus vociferans* and *Aotus nancymaae* (Platyrrhini)

**DOI:** 10.1371/journal.pone.0096973

**Published:** 2014-05-12

**Authors:** Carolina López, Carlos F. Suárez, Luis F. Cadavid, Manuel E. Patarroyo, Manuel A. Patarroyo

**Affiliations:** 1 Molecular Biology and Immunology Department, Fundación Instituto de Inmunología de Colombia (FIDIC), Bogotá, Cundinamarca, Colombia; 2 School of Medicine and Health Sciences, Universidad del Rosario, Bogotá, Cundinamarca, Colombia; 3 MSc Microbiology Programme, Instituto de Biotecnología (IBUN), Universidad Nacional de Colombia, Bogotá, Cundinamarca, Colombia; 4 Genetics Institute, Universidad Nacional de Colombia, Bogotá, Cundinamarca, Colombia; 5 School of Medicine, Universidad Nacional de Colombia, Bogotá, Cundinamarca, Colombia; University of Florence, Italy

## Abstract

Non-human primates belonging to the *Aotus* genus have been shown to be excellent experimental models for evaluating drugs and vaccine candidates against malaria and other human diseases. The immune system of this animal model must be characterised to assess whether the results obtained here can be extrapolated to humans. Class I and II major histocompatibility complex (MHC) proteins are amongst the most important molecules involved in response to pathogens; in spite of this, the techniques available for genotyping these molecules are usually expensive and/or time-consuming. Previous studies have reported MHC-DRB class II gene typing by microsatellite in Old World primates and humans, showing that such technique provides a fast, reliable and effective alternative to the commonly used ones. Based on this information, a microsatellite present in MHC-DRB intron 2 and its evolutionary patterns were identified in two *Aotus* species (*A. vociferans* and *A. nancymaae*), as well as its potential for genotyping class II MHC-DRB in these primates.

## Introduction

Using non-human primates in the field of biomedical research is useful for validating methodologies for diagnosing and treating diseases affecting human beings [1], [2]. Monkeys from the *Aotus* genus are used for studying the main types of human malaria (*Plasmodium falciparum* and *Plasmodium vivax*), being suitable models due to their susceptibility to the infection, thereby facilitating the evaluation of vaccines and drugs for treating and controlling this disease. These primates have also been used for studying leishmaniasis, schistosomiasis, hepatitis, tuberculosis and various types of enteric infection [3]–[9].

Previous studies have shown that this animal model is similar to humans regarding immune system molecules, particularly concerning MHC class II and especially those corresponding to human HLA-DR. Such similarity enables evaluating the immune response to different pathogens and evaluating the potential of molecules which are candidates for a vaccine aimed at controlling diseases of importance for human health [10]–[12].

The high degree of polymorphism and allele diversity shown by MHC-DRB molecules in humans and other primates, as well as their importance in interaction with peptides so that they can be presented to the T-lymphocyte receptor, makes their typing relevant for evaluating an immune response to malaria and vaccines designed for controlling it [13]. MHC-DR variability is mainly concentrated in MHC-DRB exon 2 and to a lesser extent in MHC-DRA exon 2 [14], both regions encoding the peptide binding region (PBR). This sector mainly defines the alleles observed in vertebrates and is subject to diversifying selection and recombination, thereby modelling its variability [15]–[17]. Twelve allele lineages have been characterised for *Aotus* MHC class II DRB, having considerable similarity with human HLA-DRB lineages [12], [18], [19].

Precise typing of MHC genes implies using laborious and costly techniques due to their complex genomic organisation (usually into different haplotypes) and their individual (expressing different genes) and population variability (polymorphism) [13]. Current techniques would include restriction fragment length polymorphism (RFLP), single strand conformation polymorphism (SSCP), denaturing gradient gel electrophoresis (DGGE), reference strand-mediated conformational analysis (RSCA) and amplifying, cloning and sequencing fragments of interest, especially exon 2. The latter represents the most precise approach but does involve some disadvantages such as its high cost and the longer time involved in obtaining results. The other approaches offer results having variable agreement with the data obtained by sequencing [20]–[22].

In addition to the above, a microsatellite located at the start of intron 2 in humans, macaques and chimpanzees has been used for typing MHC-DRB [23], [24]. Short tandem repeat (STR) polymorphism has been shown to be well-correlated with the diversity shown by exon 2. The microsatellite is basically presented as the repeat of (GT)_x_ (GA)_y_ dinucleotides, showing different degrees of complexity, according to the species being analysed [23].

Regarding HLA-DRB, the STR has been called D6S2878, being present in all HLA-DRB genes/pseudogenes, except HLA-DRB2, HLA-DRB8 and HLA-DRB9 where the first part of intron 2 is lost. It is highly polymorphic in composition and length and can specifically differentiate between HLA-DRB gene alleles [25]. This sector also exhibits polymorphism in *Macaca mulatta*, having high variability regarding length and sequence, thus allowing the characterisation of different MHC-DRB alleles in this primate [24]. DRB-STR microsatellite ancestral structure in Old World monkeys (OWM) contains a simple nucleotide repeat, whilst HLA and Mamu-DRB-associated microsatellite structure is more complex [25]. Taking into account that this microsatellite's variability pattern in humans and macaques is correlated with exon 2 polymorphism, making it an attractive option for typing these genes [25], [26], it was thus of interest to verify whether the same occurs in New World monkeys (NWM). The MHC-DRB intron 2 in Platyrrhini is very variable in length, ranging from 50 bp to 1 Kbp [27], including a simple repeat sequence of around 50 bp downstream the limit between exon 2 and intron 2 [28], [29].

The microsatellite present at the start of MHC-DRB genes' intron 2 in individuals from the *A. vociferans* and *A. nancymaae* species has thus been verified and characterised here, this being the first systematic characterisation of this marker in NWM, indicating the feasibility of its use in these primates for typing MHC-DRB.

## Materials and Methods

### Sample origin

Monkeys from the *Aotus nancymaae* (25 adults) and *Aotus vociferans* species (23 adults) were studied; they came from FIDIC's primate station in Leticia, Amazonas, Colombia. Blood samples from *A. vociferans* were collected fresh, whilst those from *A. nancymaae* had been collected in 2001. All primates were kept in conditions laid down by Colombian Ministry of Health (law 84/1989) and Colombian Institute of Health regulations for animal care, monitored weekly by CORPOAMAZONIA (resolutions 0202/1999 and 0028/2010). All procedures were approved and supervised by the Health Research Ethics Committee and FIDIC's Primate Station Ethics Committee.

The US Committee on the Care and Use of Laboratory Animals' guidelines were followed for all animal handling procedures, in turn complying with Colombian regulations for biomedical research (resolution 8430/1993 and law 84/1989).Monkeys at the station were numbered, sexed, weighed, given a physical-clinical exam and kept temporally in individual cages, prior to all experimental procedures. They were kept in controlled conditions regarding temperature (25^°^–30^°^ centigrade) and relative humidity (83%), similar to those present in their natural environment. The monkeys' diet was based on a supply of fruit typical of the Amazon region (i.e. such primates' natural diet), vegetables and a nutritional supplement including vitamins, minerals and proteins. Environmental enrichment included visual barriers to avoid social conflict, feeding devices, some branches and vegetation, perches and habitat. Any procedure requiring animal handling was undertaken by trained veterinary personnel and animals were submitted to sedation and analgesia procedures to reduce stress when necessary [30].

### Molecular characterisation of species of owl monkeys studied

Mitochondrial gene cytochrome c oxidase subunit II (mtCOII) sequences were used for determining the species to which the owl monkeys being studied belonged to, following the methodology described by Ashley & Vaughn [31]. PCR was used for amplifying the gene, using high fidelity Taq DNA polymerase. Two independent PCR reactions were performed and the amplified products were purified using a Wizard SV gel and PCR clean-up system kit (Promega, Madison, WI, USA); these were sent for sequencing with mtCOII-specific primers using the BigDye Terminator method (MACROGEN, Seoul, South Korea). The sequences so obtained were analysed for constructing phylogenetic trees and these were then compared to previously described sequences from databases for mtCOII from primates.

### DNA, RNA extraction and cDNA synthesis

Genomic DNA (gDNA) from each specimen was isolated for *A. vociferans* from 300 µL peripheral blood samples using an UltraClean Blood DNA Isolation kit (Carlsbad, CA, USA), following the manufacturer's instructions. Total RNA was isolated from 2 mL peripheral blood in EDTA diluted 1∶1 with PBS. A Ficoll-Hypaque density gradient (Lymphocyte Separation Medium, ICN Biomedicals, CA, USA) was used for isolating mononuclear cells, according to the manufacturer's recommendations. The lymphocytes so recovered were immediately homogenised with TRIzol reagent (Life Technologies, NY, USA). cDNA was synthesised with a SuperScript III First-Strand Synthesis System for RT-PCR kit (Life Technologies, NY, USA), using Oligo(dT)_20_ (Invitrogen, NY, USA) as primer, according to the manufacturer's instructions.

Genomic DNA was isolated from leucocytes for *A. nancymaae*, using a NucleoSpin C+T kit (Macherey-Nagel AG, Oensingen, Switzerland), according to the manufacturer's protocol. Total RNA was isolated from PBMC using a NucleoSpin RNA kit (Macherey-Nagel AG, Oensingen, Switzerland), according to the manufacturer's recommendations. Reverse transcription was performed using SuperScript and Oligo(dT)_12–18_ primer (Gibco BRL Life Technologies, Basel, Switzerland). Both gDNA and cDNA were preserved in 95% ethanol at −80°C until use. DNA integrity was verified by electrophoresis on 1% agarose gel, stained with SYBR Safe (Invitrogen) for visualisation under UV light. NanoDrop 2000 (Thermo Scientific) was used for calculating the concentration.

### Amplifying, cloning and sequencing

**Figure 1:**
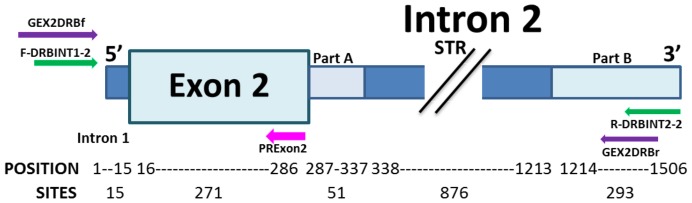
Diagram of the MHC-DRB region studied. The primers used for amplifying the exon 2+ intron 2 (partial) from gDNA are shown as arrows (purple and green); the PRExon2 primer was designed for confirmatory colony PCR (pink arrow). The MHC-DRB amplified sector (exon 2, intron alignable sectors 2 (A and B) and STR) was partitioned for sequence analysis (position and sites).

The primers used here were designed by aligning available genome sequences for the *Callithrix jaccus*, *Homo sapiens* and *Macaca mulatta* MHC-DRB region (Table S1 in File S1), using Netprimer software [32] for optimising parameters. Two sets of primers were used for amplifying exon 2+ intron 2 sequences. The first primer set included direct primer GEX2DRBf (5′-GGTCAAGGTTCCCAGAGC-3) to the end of intron 1 and reverse GEX2DRBr (5′-CTCCAAGGATAAGAAGAAGCC-3′) located about 100 bp downstream of the end of the microsatellite. The second set included direct primer F-DRBINT1-2 (5′-TTCGTGTCCCCACAGCAC-3′) to the end of intron 1 and reverse R-DRBINT2-2 (5′-TAAACCCTCACCCCAGCC-3′) situated about 160 bp downstream of the end of the microsatellite (Figure 1). Direct primer DRBExon1PF (5′-CACTGGCTTTGGCTGGGGAC-3′) in exon 1 was used for amplification from cDNA with either DRBExon6PR1 (5′-CCACAAGGGAGGACATTTCTGC-3′) or DRBExon6PR2 (5′-CCAAGGGCAGAAGCTGAGGAA-3′) reverse primers in exon 6.

Two independent PCR reactions were carried out for each primate; the reactions followed recommendations made by Lenz *et al*., [33] for avoiding chimera formation. The KAPA HiFi HotStart Readymix enzyme (Kapa Biosystems, Woburn, MA, USA) was used with 0.3 µM each primer and 10–40 ng DNA (in the case of gDNA) or 2 µL recently synthesised cDNA for 25 µL final volume. The PCR reaction at saturation was carried out in a PerkinElmer GeneAmp 9600 thermocycler. The following thermal profile was used for cDNA: 95°C for 5 min, 35 cycles at 98°C for 20 s, 66°C/67°C (when using the first or the second reverse primer, respectively) for 15 s, 72°C for 30 s and a final 5 min extension step at 72°C. The following thermal profile was used for gDNA: 95°C for 5 min, 35 cycles at 98°C for 20 s, 57°C/66°C (for the set of primers 1 or 2, respectively) for 15 s, 72°C for 30 s and the final extension step at 72°C for 5 min.

Amplified products were purified using a Wizard SV Gel and PCR Clean-Up System kit (Promega, USA) and a protocol was used for extending A with GoTaq Flexi DNA polymerase (Promega) to enable ligating them with the pGem-T Easy Vector Systems (Promega, Madison, WI, USA)vector, following the manufacturer's recommendations. The transformation was carried out in *Escherichia coli* JM109 strain competent cells. The clones were selected using positive selection with ampicillin and lacZ gene α-complementation. Plasmid DNA was extracted using an UltraClean 6 Minute Mini Plasmid Prep kit (MO BIO, USA).

Given that other targets were observed for the pairs of primers used for amplifying the exon 2+ intron 2 STR sector, a primer was designed at the end of exon 2 (PRExon2) (5′-TCGCCGCTGCACTGTGAAG-3′), enabling confirmatory colony PCR, using those used in amplifying gDNA as direct primers (Figure 1). The reaction contained 1 µL enzyme buffer, 0.6 µL MgCl_2_ [25 mM], 1.6 µL dNTPs [1.25 mM], 0.8 µL of each primer [5 µM], 0.12 µL GoTaq Flexi DNA polymerase (Promega) and 10–40 ng colony DNA at 10 µL final volume. PCR conditions consisted of one cycle at 95°C for 5 min, 35 cycles at 95°C for 1 min, 60°C for 1 min, 72°C for 1 min and a final extension step at 72°C for 5 min.

At least 8 clones (confirmed from each amplification) were selected for sequencing; their DNA was sequenced in both directions using T7 and SP6 primers, following the BigDye Terminator method (MACROGEN, Seoul, South Korea).

### Sequence analysis

The MHC-DRB sequence electropherograms were assembled using CLC Main Workbench software v.5 (CLC bio, Cambridge, MA, USA). The sequences so obtained had to comply with the following requirements to be considered as being valid: having been found in at least two independent PCR from the same individual, or coming from two different individuals (including previously reported sequences in this category). The alleles found were validated and named by a curator from the Immuno Polymorphism Database (IPD) [34], [35].

Clustal X software (v2.1) was used for aligning all the MHC-DRB exon 2 and exon 2+ intron 2 sequences [36], using BioEdit Sequence Alignment Editor software for manual editing [37].

MEGA software (v5.2) was used for selecting the best nucleotide substitution model using Bayesian Information Criteria (BIC); phylogenetic trees were constructed using minimum evolution, neighbour joining, parsimony and maximum likelihood methods. The bootstrap test was used for supporting the trees so obtained, in addition, the interior branch test was used for supporting trees constructed using the minimum evolution and neighbour joining methods. 1,000 replicates were carried out; those groups having greater than or equal to 70% by bootstrap and greater than or equal to 95% by interior branch test were considered as supported groups [38], [39].

### Microsatellite analysis

Microsatellite search and building database (MSDB) software [40] was used for identifying the microsatellite, using the imperfect search mode; valid repeats were considered as those having 12 or more mononucleotide segments and repeats having 4 or more di-tri-tetra-penta-hexa nucleotides. Their descriptors were constructed using previous results and manual edition as guidelines. A compressibility method was used, given the difficulty of obtaining an unambiguous alignment of repeat sectors when they were analysed exclusively. The sequences were organised as 100 tandem repeats and compressed into separate files using an adaptive Lempel-Ziv algorithm (using the Linux command *compress*). From the resulting vector obtained from the bytes for each compressed sequence, a distance matrix was then calculated using either the Euclidean, Maximum or Manhattan metrics through the DIST package from R [41].Hierarchical clusters were constructed with the R hclust package [41], using single and complete methods.

## Results

**Figure 2:**
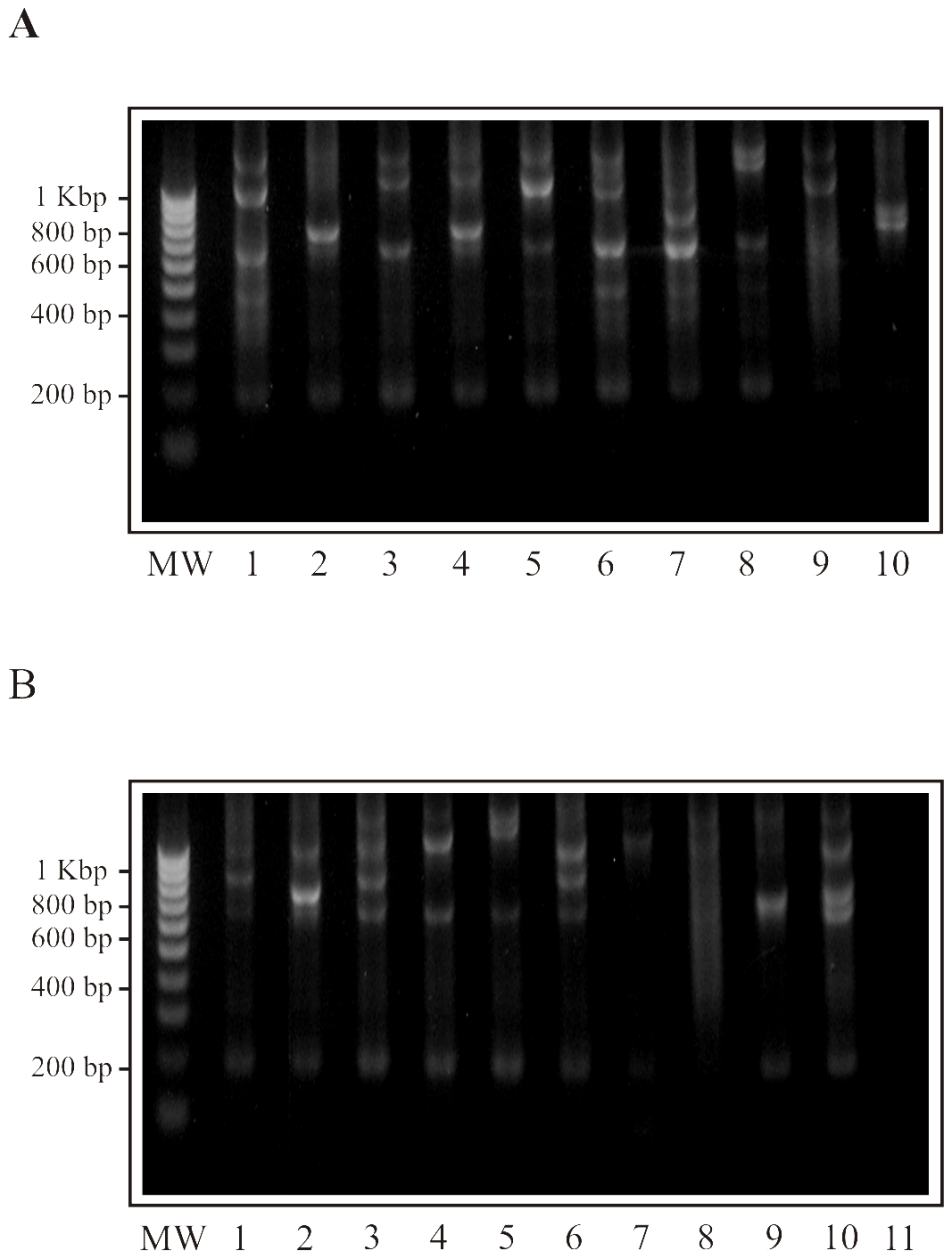
*A. nancymaae* and *A. vociferans* exon 2+ intron 2 partial amplicons. Amplicons ranging from ∼700 bp to ∼1,000 bp were obtained from *A. vociferans* and *A. nancymaae* samples. A. Lanes 1–10 show *A. nancymaae* amplicons. B. Lanes 1–10 show *A. vociferans* amplicons, lane 11 negative control. MW. molecular weight.

Amplicons ranging from ∼700 bp to ∼1,000 bp were obtained for *A. vociferans* and *A. nancymaae* samples (Figure 2); 289 sequences were obtained from exon 2+STR intron 2. One to five different MHC-DRB sequences per animal were observed from two independent PCR reactions; this implied the duplication of this loci, as has been reported previously [12]. A total of 34 distinct nucleotide sequences were validated, 28 of which were also isolated from cDNA: two new sequences belonging to two new *A. nancymaae* lineages (Aona-DRB*W9101 and Aona-DRB*W8901), 7 new sequences belonging to five new *A. vociferans* lineages (Aovo-DRB*W9101, Aovo-DRB*W9102, Aovo-DRB*W9201, Aovo-DRB*W9202, Aovo-DRB*W9301, Aovo-DRB*W8801, Aovo-DRB*W9001), 11 new sequences from previously reported *A. vociferans* lineages (Aovo-DRB1*0304, Aovo-DRB1*0305, Aovo-DRB1*0306, Aovo-DRB1*0307, Aovo-DRB3*0601, Aovo-DRB*W1801, Aovo-DRB*W1802, Aovo-DRB*W1803, Aovo-DRB*W2901, Aovo-DRB*W3001, Aovo-DRB*W4501), 6 new from previously reported *A. nancymaae* lineages (Aona-DRB1*031701, Aona-DRB1*0329, Aona-DRB3*062502, Aona-DRB3*0628, Aona-DRB*W1808, Aona-DRB*W3002) and 8 already reported sequences for *A. nancymaae* lineages (Aona-DRB1*0328, Aona-DRB3*0615, Aona-DRB3*062501, Aona-DRB3*0626, Aona-DRB3*0627, Aona-DRB*W1806, Aona-DRB*W2908, Aona-DRB*W2910) (see Table S1 in File S1).

The MHC-DRB amplified sector was divided into the following partitions for sequence analysis: intron 1 (positions 1–15: 15 sites), exon 2 (positions 16–285: 270 sites), intron 2A (alignable; positions 286-325: 40 sites), intron 2R (STR sector; positions 326–1,110: 785 sites), intron 2B (alignable; positions 1,111–1,378: 268 sites) (Figure 1). These size ranges were related to aligning the sequences given in Figure S1 (within File S1).

Greater conservation of alignable areas was observed in intron 2 (A+B, 95±1% identity) compared to exon 2 (91±1% identity). An unambiguous alignment could not be made for intron 2 STR. This had substantial variation regarding its size, representing an 83 bp (Aovo-DRB*W9301) to 761 bp (Aona-DRB1*0329GA) interval.

**Figure 3:**
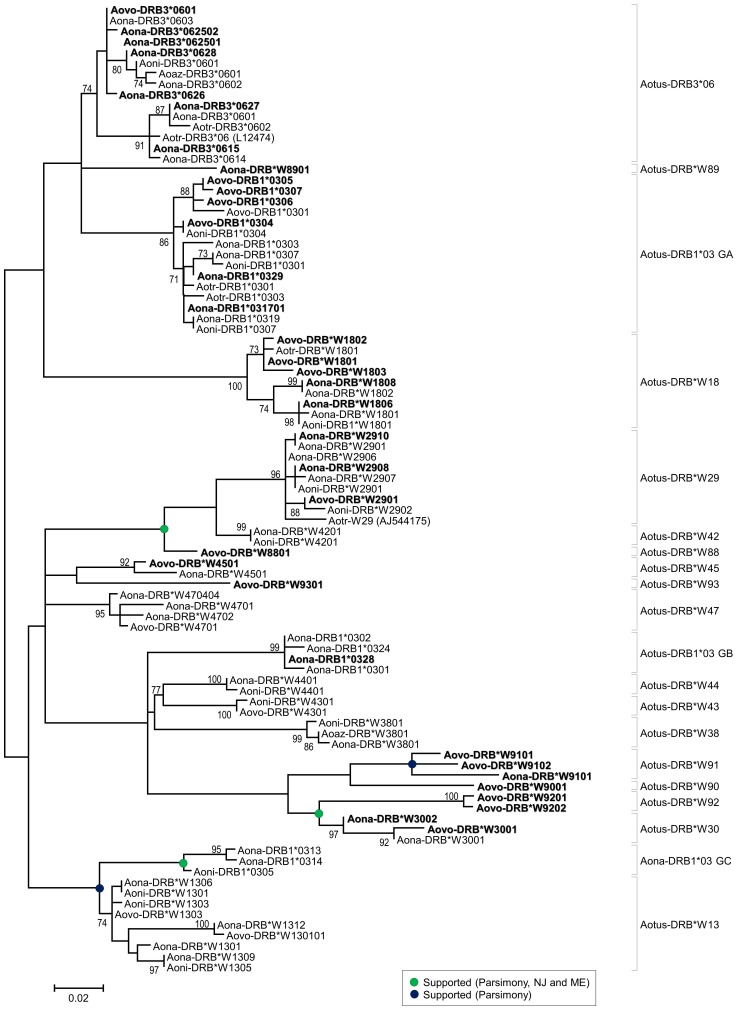
Maximum likelihood tree constructed from *Aotus* MHC-DRB exon 2 sequences (91 OTUs, 268 aligned positions). The analysis involved using the general time reversible model with invariable positions and Gamma distribution (5 categories, +G, parameter  = 0.3371), >70% bootstrap values are displayed. Green dots represent nodes supported by parsimony (>70% bootstrap), Neighbour joining and minimum evolution tests (>70% bootstrap and >95% interior branch test), but not in maximum likelihood analysis. Nodes represented by blue dots were supported only by parsimony (>70% bootstrap), but not in the maximum likelihood analysis. Bootstrap and interior branch tests involved using 1,000 replicates. The scale bar represents substitutions per site. New sequences reported in this study are shown in bold. Abbreviations and GenBank accession numbers for the sequences compared here are shown in Table S1 (within File S1).

Exon 2 in the sequences reported here were analysed together with 57 representative sequences of *Aotus* MHC-DRB allele lineages reported in previous studies by Suárez *et al*., and Niño *et al*.,[12], [18] and others available in Genbank. The evolutionary analysis methods described in the methodology were used on an alignment of 268 positions. Figure 3 shows the tree with the maximum likelihood method using a GTR+G+I model.

The alleles observed came from some lineages previously reported by Suárez *et al*., [12] thereby highlighting the existence of seven new lineages. Most lineages were supported by all the phylogenetic reconstruction and support methods (those only supported by some of them are indicated by circles in the node); however, the relationships between such lineages had low support (Figure 3). Based on the sequences studied here, most observed lineages were trans-specific, DRB1*03 GB and DRB*W89 lineages being species-specific for *A. nancymaae* and DRB*W88, DRB*W92, DRB*W90, DRB*W45 and DRB*W93 for *A. vociferans*.

**Figure 4:**
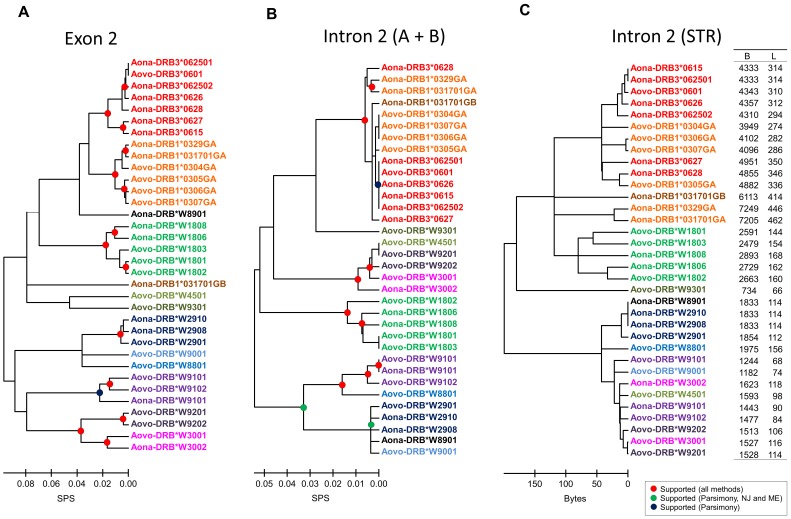
Comparison amongst exon 2, alignable sectors of intron 2 and intron 2 STR. A. Maximum likelihood tree constructed from *Aotus* MHC-DRB exon 2 sequences (34 OTUs, 344 aligned positions). The analysis used the Hasegawa-Kishino-Yano model with invariable positions and Gamma distribution (5 categories, +G, parameter  = 0.2659, +I, 51.7393% sites). **B. Maximum likelihood tree constructed from** ***Aotus*** **MHC-DRB intron 2 (A+B) sequences (34 OTUs, 271 aligned positions).** The analysis involved using the Hasegawa-Kishino-Yano model with invariable positions and Gamma distribution (5 categories, +G, parameter  = 0.2378, +I, 0.0% sites). **C. Complete linkage tree constructed from** ***Aotus*** **MHC-DRB intron 2 STR sequences.** The analysis was done using a Manhattan distance over Lempel-Ziv compression. Compression in bytes (B) and length in nucleotides (L) are also shown. Nodes indicated by red dots were supported by all methods. Nodes shown by green dots were supported by parsimony (>70% bootstrap), Neighbour joining and minimum evolution tests (>70% bootstrap and >95% interior branch test), but not in maximum likelihood analysis. Nodes represented by blue dots were supported only by parsimony (bootstrap >70%), but not in maximum likelihood analysis. Bootstrap and interior branch tests were performed using 1,000 replicates. The scale bar represents substitutions per site (A and B), and bytes (C). Abbreviations and GenBank accession numbers of the analysed sequences are shown in Table S1 (within File S1).

Molecular phylogenetic analysis was made regarding the 34 sequences reported here, examining separately either exon 2 or the concatenated intron 2 alignable sectors (2A+2B) using previously described evolutionary analysis methods. Figure 4A shows the tree obtained by aligning exon 2 sequences (271 positions) with the maximum likelihood method, using a HKY+G+I model. Figure 4B shows the tree obtained by aligning intron 2 alignable sectors (344 positions) using the maximum likelihood method and an HKY+G+I model.

Most groups' identity was maintained regarding intron 2 alignable sectors compared to those observed in exon 2, although some became fused (i.e. DRB3*06 - DRB1*03 GA, DRB*W45 - DRB*W92 and DRB*W89 - DRB*W29), changing their relationships for each partition. However, lineage differentiation was well supported and even the association between some lineages (e.g. DRB3*06 - DRB1*03 GA, DRB*W30 – DRB*W92) was very clear, being maintained for the sets of data and methods analysed.

Compressibility was used for estimating similarity between sequences, given that the intron 2 repeat sector was not unequivocally alignable due to its repeat nature. The Lempel–Ziv algorithm was used with the Linux standard command *compress* for compressing files. Each sequence was repeated 100 times in tandem to ensure better resolution, so that files were 734–7,249 bytes after having been compressed (Figure 4C). Equivalent results were obtained using different metrics and grouping/clustering methods. Figure 4C shows the results using Manhattan metrics and the complete linkage agglomeration method. The STR grouping pattern is an intermediate between that of exon 2 and that generated from intron 2 A+B sectors.

It was observed that DRB3*06 and DRB1*03 GA lineages were associated in all the sectors analysed, being included in this grouping the DRB1*03GB lineage sequence in intron 2 A+B sectors and in STR. Each lineage's definition became lost in the STR, Aona-DRB1-0329GA, Aona-DRB1-031701GA and Aona-DRB1-0328GB sequences being differentiated by differences in STR length but being maintained in a common cluster with the remaining DRB3*06 and DRB1*03 sequences.

DRB*W88, DRB*W29, DRB*W30, DRB*W92, DRB*W91 and DRB*W90 lineages were associated in both exon 2 and the STR, the difference being that DRB*W89 and DRB*W45 lineages were inserted in the latter analysis, grouping with DRB*W29 and DRB*W30/*W91 lineages, respectively, in the STR and intron 2 A+B sectors. DRB*W89 and DRB*W45 were grouped in exon 2 with the DRB1*03GA - DRB3*06 - DRB*W18 group. The DRB*W30 and DRB*W92 lineages formed a cluster with the DRB1*03GA and DRB3*06 group in the intron 2 A+B sectors. The DRB*W18 lineage was always well characterised, having a cluster in STR and exon 2 which included DRB1*03GA - DRB3*06 – DRB1*03 GB lineages. The DRB*W92/*W91/*W45 lineages were also included in intron 2 A+B sectors in this group.

The DRB*W93 lineage appeared in all analysis as a divergent member of the cluster formed by DRB3*06 - DRB1*03 GA - DRB1*03 GB - DRB*W18 and was related to the DRB*W45 lineage in exon 2, losing such relationship in intron 2. This lineage had a similar pattern to that of DRB*W89, whose grouping was very different between exon 2 and intron 2.

MSDB software [40] was used for characterising the amplified sequences (exon 2+ intron 2 (partial)) for analysing motifs (Table S2 in File S1). The different types of microsatellite agreed with the results found by the compression method.

It was observed that the microsatellite was characteristic for some lineages, being clearly differentiated by length and structure, forming 3 groups which included the 34 sequences described for the *Aotus* species included in this study. The STR could be divided into 3 sectors (Table S2 in File S1), the initial and final sectors being similar in all sequences; greater variability (intra and inter lineages) was observed in the microsatellite's central region. (GA)_y_ was the main repeat motif found in all cases.

The STR had a similar structure throughout the DRB1*03 and DRB3*06 lineage sequence repeat sector, but there were differences regarding the number of repeats. The microsatellite had lengths ranging from 294 to 354 bp in *A. nancymaae* and *A. vociferans* DRB3*06 lineage sequences, a very similar structure being maintained in the initial and final part. There were slight differences in the repeats towards its central part and identical sequences were even observed in the STR, such as Aona-DRB3*062501/*0615. The DRB1*03 lineage sequences did not have a specific STR pattern, length varying from 274 to 462 bp. However, two defined groups were identified, one for the Aovo-DRB1*0304, 1*0307 and 1*0306GA sequences and another for the Aona-DRB1*0328GB, Aona-DRB1*031701 and 1*0329GA sequences, having similar structure and length. The Aona-DRB1*0329GA and *031701GA sequences had very similar distribution, having minimal differences regarding length at the start of the STR. Aovo-DRB1*0305GA had an STR having a particular structure, but maintaining similarity concerning lineage. The Aona-DRB3*0627 and Aona-DRB3*0628 sequences' repeat sector had similar distribution with DRB1*03 lineage sequences regarding repeats and length.

Regarding DRB*W18 lineage sequences, the STR had a size ranging from 144 to 160 bp, having similar distribution concerning composition and number of repeats at the beginning and end of the STR. Each sequence varied specifically at the central part in both nucleotide sequence and number of repeats. The Aovo-DRB*W9301 sequence had a 66 bp STR, being the smallest of all the sequences. It maintained a similar structure in the initial and final part to that described in other lineages, having a relatively short central region (26 bp).

The microsatellite had similar structure at the start and end in the DRB*W89/*W29/*W88/*W90/*W91/*W45/*W30/and *W92 lineages, having a length ranging from 68 to 156 bp. Various sequences had practically identical STR in this group, such as Aovo-DRB*W9201 and Aovo-DRB*W3001 (only one repeat being different), or identical STR, such as Aona-DRB*W8901, Aona-DRB*W2910 and Aona-DRB*W2908. Regarding this group, the Aovo-DRB*W2901 sequence had very similar organisation in the STR, having slight differences regarding structure and the number of repeats, given that even though belonging to the same lineage (W29), it came from a different species. The Aovo-DRB*W8801 sequence was similar to the DRB*W29 lineage, but had differences concerning the number of repeats in the central region. The Aovo-DRB*W9102 and Aona-DRB*W9101 sequences in the DRB*W91 lineage had similar microsatellite structure, having few differences concerning the number of repeats in the central region.

Regarding primates, 34 sequences from the MHC-DRB gene's exon 2+ intron 2 (partial) were analysed in *A. nancymaae* and *A. vociferans*; sequences related to the sector being studied were selected from previous typing reports [14], [27] and a search of available complete or ongoing primate genomes using the BLAST algorithm [42]. This led to 86 primate sequences being included, including representatives for distinct human lineages (Table S1 in File S1). Clustal X v2.1 software was used for aligning the sequences [36]; these were then edited manually (especially in the repeat sector). The MHC-DRB sector was divided into the partitions shown in Figure 1 for their analysis.

A satisfactory alignment could not be made for the intron 2 repeat area (which is why it has not been considered in the phylogenetic analysis); however, the alignable sectors from intron 2 (A and B) had a notable degree of identity (90±0.8% for all primates), this being 94.1±0.7% for NWM and 90.2±0.7% for OWM. Such degree of conservation was even greater than that observed for exon 2, whose average identity for the primates studied here was 87.3±0.1% (similar values being obtained for both OWM and NWM). The intron 2 repeat region had notable variation regarding length between the primates analysed here; however, the presence of a central motif (GA)y was constant, being very idiosyncratic for each allelic lineage analysed. The sequences obtained from the *C. jacchus* genome were illustrative in this respect; whilst Caja-DRB*04 only had 3 base pairs in the repeat sector, Caja-DRB*05 was 849 bp.

**Figure 5:**
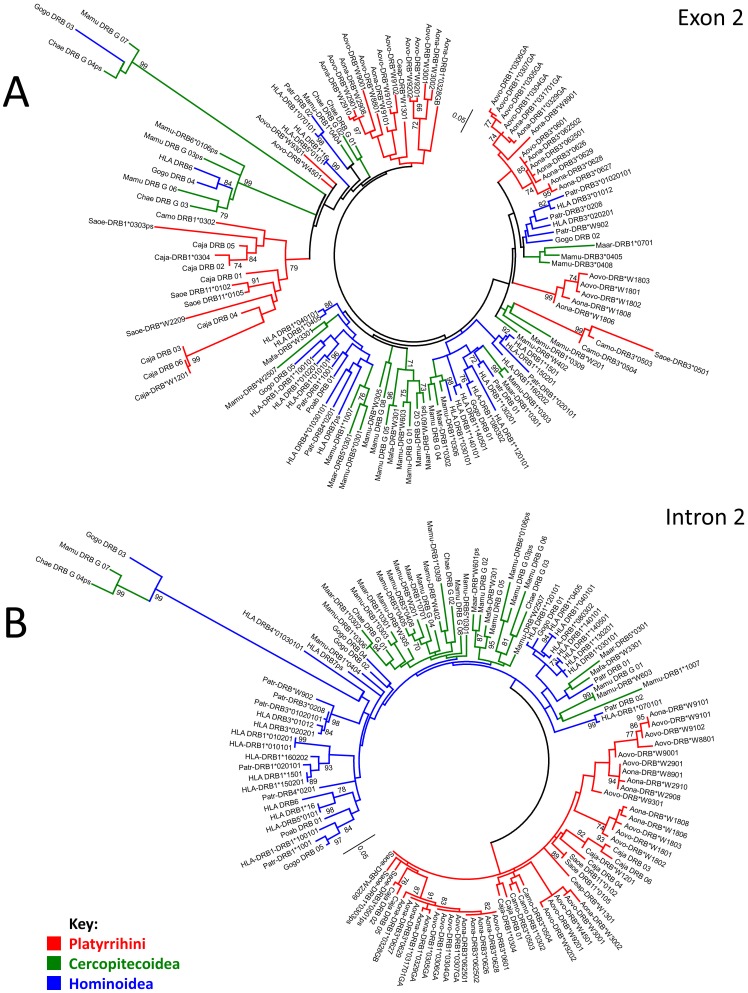
Maximum likelihood trees. A. Maximum likelihood tree constructed from *Aotus* MHC-DRB exon 2 sequences (120 OTUs, 271 aligned positions). The analysis involved using Kimura's 2 parameter model with invariable positions and Gamma distribution (5 categories, +G, parameter  = 0.5550). **B Maximum likelihood tree constructed from** ***Aotus*** **MHC-DRB alignable sectors of intron 2 (132 OTUs, 359 aligned positions).** The analysis involved using the general time reversible model with invariable positions and Gamma distribution (5 categories, +G, parameter  = 1.2072). >70% bootstrap values are displayed. The bootstrap test involved using 1,000 replicates. The scale bar represents substitutions per site. Abbreviations and GenBank accession numbers for the sequences compared here are shown in Table S1 (within File S1).

The selected sequences were subjected to two molecular phylogenetic analysis; one used just exon 2 and another used intron 2 alignable sectors (A+B). Figure 5A shows the maximum likelihood analysis for exon 2. Several Catarrhini and Platyrrhini sequences were associated, presenting a mixture of alleles from both types of primate in several groups. For Catarrrihini, some groups were formed by a mixture of species belonging to different genera and families. This did not happen for NWM; the Callitrichidae maintained their identity in well-supported nodes, whilst the *Cebus* sequence was associated with one of the groups of sequences formed by *Aotus* sequences.

Figure 5B shows the maximum likelihood analysis for intron 2 alignable sectors (2A+2B). having clear division of Platyrrhini and Catarrhini sequences. Regarding Catarrhini, most groups were seen to be well-differentiated, being mainly groups exclusively containing Anthropoidea (*Homo, Pan, Gorilla*) or Cercopithecoidea (*Macaca, Chlorocebus*), few cases involving both groups occurring simultaneously. A genus-specific disposition predominated in Platyrrhini. The *Aotus* sequences were configured into three groups, whilst Callitrichidae formed multiple genus- specific clusters. The result for this sector was similar to that observed for exon 2+ intron 2 (A+B) (not shown).

## Discussion

Analysis of *Aotus* MHC-DRB gene exon 2 sequences showed how the number of trans-specific lineages for the genus were increased and defined by improving *A. vociferans* sampling. Except for DRB*W41, DRB*W43, DRB*W44, DRB*W38, DRB*W42, DRB*W47, DRB*W13 and DRB1*03GC lineages, the remaining *Aotus* lineages were sampled in the present study (Figure 3).

The definition of two sub-lineages could be observed in lineages like DRB*W18 (having no report of alleles for *A. vociferans*), one belonging to species typically from the north of the Amazon region (*A. vociferans* and *A. trivirgatus*) and another related to species typically from the south of the Amazon region (*A. nancymaae* and *A. nigriceps*). Such tendency (although less marked) was observed for the DRB1*03GA lineage where a well-supported sub-lineage was exclusively grey-neck (there were also exclusively red-neck sub-lineages). An *A. vociferans* sequence (Aovo-DRB3*0601) was reported for the DRB3*06 lineage (apparently exclusive to red-neck monkeys) which was identical to an *A*. *nancymaae* sequence (Aona-DRB3*062501). This was also true for the DRB*W45 and DRB*W30 lineages where *A*. *vociferans* sequences were described (Figure 3). Apparently exclusive lineages exist, such as the DRB1*03GB lineage, which has just *A. nancymaae* sequences; however, differing degrees of trans-specificity were observed in the rest of the lineages, even though there could be specific sub-lineages.

There were differences regarding frequencies but not regarding the repertoires of the two *Aotus* species studied here, indicating that each had undergone diversification; however, they maintained notable identity between their MHC-DRB repertoires over a relatively long period of time (from 13–8 mya) [43]. Such trans-specific polymorphism in repertoires suggests that using both species as animal models could be equivalent for MHC-DRB-mediated processes [44].

Comparative analysis of *Aotus* DRB genes' exon 2 phylogenies (Figure 4A) and intron 2 alignable sectors (Figure 4B) showed that some of the lineages clearly maintained their identity, whilst others became merged. The relationship between lineages also changed from one sector to another, groups of well-supported lineages becoming formed in analysis of intron 2 (this did not happen in exon 2). The degree of intron 2 A+B sector conservation was notable compared to exon 2, thereby highlighting the magnitude of the latter's selection process.

Differential grouping showed that distinct forces have modulated each DRB gene sector's evolution, thereby posing the question, “Which one reflects more accurately the origin of DRB genes en *Aotus*?” If the intron 2 alignable sectors were to be chosen (given that they apparently have not undergone the previously described phenomena generating diversity in exon 2), then one would have a scenario where the number of lineages would be less than that proposed based on exon 2 polymorphism, and the relationships between them would have been different. Positive selection and recombination would thus have generated variability which would have grouped (by convergence and/or recombination) the sequences in previously described lineages. If exon 2 were to be chosen, the scenario would be marked by intron 2 recombination which would lead to the different groups' homogenisation in fewer lineages.

Recombination substantially affects support for trees [45], [46], thereby making the first scenario more probable, given that the tree for the intron 2 alignable sectors was better supported than that for exon 2. However, complete DRB gene sequences (including coding and non-coding sectors) are needed to clarify this point.

STR in *Aotus* mainly had (GA)_y_ repeats interrupted by CT motifs and a similar structure between sequences at the 5′ and 3′ extremes belonging to the same group according to phylogeny for the intron 2 alignable sectors (Figure S1 and Table S2 in File S1, Figure 4B). The (GA)_y_ repeats form part of the ancestral structure described for *Catarrhini* [29], [47], [48].

The *Aotus* MHC-DRB microsatellite is variable in length, as has been described for humans, macaques and chimpanzees. Exon 2 analysis led to observing that the microsatellite for the DRB3*06 lineage (the Aovo-DRB3*0601, Aona-DRB3*062502, Aona-DRB3*0626, Aona-DRB3*0628 and Aona-DRB3*0627 sequence group) could differentiate them due to their variable length, except for the Aona-DRB3*062501 and Aona-DRB3*0615 sequences which had identical length and sequence, meaning that sequencing methods were needed for identifying these alleles.

The microsatellite had highly variable length in the DRB1*03GA, DRB*W18, DRB*W91, DRB*W93, DRB*W88, DRB*W90, DRB*W91, DRB*W45 and DRB*W30 lineage and could differentiate the sequences to which it belonged in *A. nancymaae* and *A. vociferans*, except for the Aona-DRB*W8901, Aona-DRB*W2910/*W2908 and Aovo-DRB*W9201 sequences where the microsatellite had the same length thereby differentiating it as a group, but not individually, and thus working as a screening but not as a typing method for these alleles.

**Figure 6:**
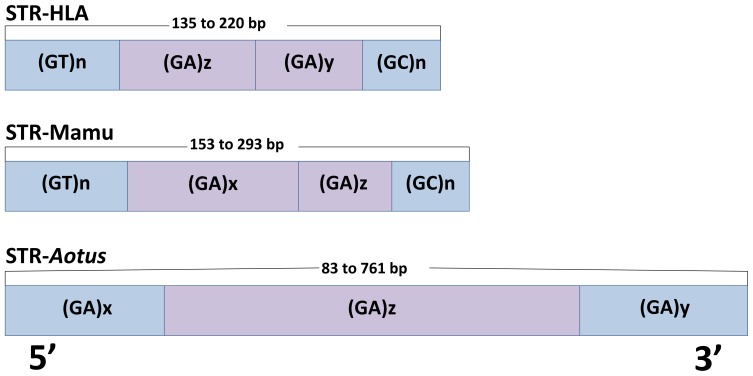
MHC-DRB STR model for Platyrrhini *cf* Catarrhini. The Figure shows the STR structure described by Bontrop *et al*., for Human HLA-DRB (STR-HLA) and *Macaca mulatta* MHC-DRB (STR-Mamu); and our proposed *Aotus* MHC-DRB model (STR-*Aotus*). The lengths ranges for each STR are shown. The ancestral structure of the microsatellite in Catarrhini has evolved from dinucleotide repeats (GT)x (GA)y; the (GA)y dinucleotide in *Aotus* was maintained in STR structure and the (GT)x repeat was not present. STR in *Aotus* mainly had (GA)y repeats interrupted by CT motifs, this being more complex and bigger than Catarrhini STR.

According to the results reported here, the composition of the microsatellite described for MHC-DRB sequences in *A. nancymaae* and *A. vociferans* was more variable and complex than in humans and other *Catarrhini* (Figure 6). Comparison of the groups deduced from exon 2 and those observed for the STR was not always consistent, just as in previous reports concerning OWM published by Bontrop *et al*., [23], [24], [49].

The ancestral structure of the microsatellite in *Catarrhini* has evolved from dinucleotide repeats (GT)_x_ (GA)_y_. Current structure of the HLA- and Mamu-DRB-associated microsatellite was seen to be more complex (Figure 6). The repeat in the 5′ extreme was the longest, uninterrupted part; the second part (GA)z was short and interrupted by other dinucleotides, being able to correlate well with different DRB gene lineages. The length of the third segment (GA)y could also be correlated with some DRB gene lineages in *M. mulatta*. The 3′ extreme consisted of a short (GC)n repeat part. It is known that mutation tendency depends on repeat length, since there is less microsatellite stability in the longer dinucleotide repeats than in the shorter ones [23], [28], [47].

The (GA)_y_ dinucleotide in *Aotus* was maintained in STR structure and the (GT)_x_ repeat was not present. Initial and final extreme repeat length in the microsatellites was similar between lineages, whilst repeat composition and number in the middle part could have been associated with specific lineages, sequences or groups; this could have been explained by the inherent differences in mutation rate between the different parts of the microsatellite.

The *A. nancymaae* and *A. vociferans* MHC-DRB microsatellite was present in all the DRB genes studied here, having considerable differences regarding length and variability, enabling it to differentiate some lineages, and even DRB sequences, thereby agreeing with exon 2 diversity. STR variability in other primate species was not always consistent with a given lineage; however, others could be characterised by a unique pattern [23], [26].

Analysis of the repeat region of 5 sequences from another Platyrrhini genus, *Callithrix jacchus* (Caja-DRB*01/*02/*03/*05/*06), revealed the same organisational pattern described for *Aotus*, having a (GA)_y_ repeat in the central sector which was complex, interrupted by CT motifs, highly variable in length and number of repeats; it came within the same ranges observed for *Aotus*, having 130-554 bp repeats. The initial and final parts of the Caja-DRB STR had similar length and sequence, the initial part being similar to that for *Aotus*, but having a more complex final part (Table S2 in File S1).

Using techniques which did not require sequence alignment for comparing them was useful in cases where this was impractical (i.e. analysis of complete genomes). As compression gives a basic measurement of a sequence of characters' algorithmic complexity, it could be especially useful when dealing with biological sequences. Using Lempel-Ziv complexity as a tool for data-mining and classifying nucleic acid and protein sequences has already been proposed [50], [51].

Compression in the present work measured two relevant parameters in microsatellite analysis, given that compressed size (in bytes) would have depended on a sequence's length and degree of simplicity (monotony), being very correlated with length in this case (R^2^ = 0.9793) given that the repeats between sequences were the same type and had the same complexity, mainly varying regarding number (Figure S1 and Table S2 in File S1).

Results for the repeat sector and exon 2 and intron 2 alignable sectors (Figures 4A and B) highlighted sector agreement. There were two large groups, one formed by DRB3*06, DRB1*03, DRB*W18 and DRB*W93 and another formed by DRB*W29, DRB*W91 and DRB*W88, DRB*W89, DRB*W45, DRB*W30 - DRB*W92 lineages being associated with one of the two, according to the sector being analysed. The DRB*W89 and DRB*W45 lineages had the greatest differences regarding grouping pattern between exon 2 and the STR, whilst this occurred between the STR and intron 2 alignable sectors in DRB*W30 - DRB*W92 groups.

There was no differentiation between lineages for the DRB3*06-DRB1*03, DRB*W89, DRB*W29, DRB*W30, DRB*W92 and DRB*W91 groups, suggesting that exon 2 origin and diversity represented a characteristic which could have been derived from a less diverse original set. This agreed with the origin of NWM arising from these primates' African transfer during the Eocene age (35 mya) [52], implying that current class I and class II MHC lineages were generated from a founding event [53].

Phylogenetic analysis of MHC-DRB gene exon 2 in primates (Figure 5A) highlighted the difficulty of inferring this gene's evolutionary relationships based just on this sector. Previous studies [12], [27], [54] have shown that even though the alleles being studied have been associated in assigned lineages, there has been poor support for such relationships, given the occurrence of phenomena guaranteeing PBR functional and structural stability. However, as a response to the diversity exhibited by pathogen proteins as a mechanism for avoiding the immune response, variation in the PBR has been produced by several mechanisms, thereby establishing a co-evolutionary arms race [55]. The most relevant features would include balanced selection (for conserving both functional integrity and diversifying the receptor) and recombination (intra-locus and inter-loci) [15]–[17], [56].

Analysis of just exon 2 has revealed the occurrence of groups of multiple primate species, thus showing the existence of groups containing Platyrrhini and Catarrhini sequences, even though most groups of sequences were biased regarding the types of primate forming them (i.e. showing some group as being predominant) (Figure 5A). The inferences drawn regarding exon 2 did not lead to concluding whether such grouping reflected a common origin for these lineages or convergence.

Concerning the particular case of MHC-DRB, molecular convergence at exon 2 level has been described in both primates [27], [53], [57] and other orders of mammals [58]–[60]. Evidence sustaining such observation has been based on independent analysis of other MHC-DRB sectors not implicated in PBR formation, where sequences belonging to Catarrhini and Platyrrhini have been shown to cluster apart, whilst for exon 2, they cluster within common allelic lineages [27], [53], [57], thus favouring the appearance of common motifs between different lineages, thereby contributing towards reducing bootstrap support [45].

Phylogenetic comparison of exon 2 (Figure 5A) and intron 2 alignable sectors (Figure 5B) from the *Aotus* sequences so obtained and a representative sample from other primates, showed that whilst the last displays a clear division between Platyrrhini MHC-DRB sequences (shown in red) and Catarrhini (Hominoidea in blue and Cercopithecoidea in green), the analysis of exon 2 presented a mixture of alleles from both types of primate, and thus molecular convergence between several groups is observed. This agreed with previous reports [27], [53].

Differently to the convergence regarding phenotypical features, convergence at molecular level is a rare phenomenon producing the same effect as another phenomenon which has shaped MHC evolution, trans-specific polymorphism, implying the maintenance of allele diversity going beyond speciation events due to balanced selection [61].

The extent of the convergence between related groups' lineages has not been previously described for DRB genes in primates; our analysis showed that the phylogenies obtained from exon 2 and those obtained for intron 2 differed regarding the relationship inside Platyrrhini and Catarrhini. The occurrence of groups containing Hominoidea and Cercopithecoidea sequences was greater in analysis inferred from exon 2 (Figure 5A) than in clusters obtained from intron 2 (Figure 5B). The same was true for Platyrrhini, where the *C. apella* sequence appeared to be included within a group of *Aotus* sequences in analysis of exon 2 (Figure 5A), whilst this did not occur regarding inference from intron 2 (Figure 5B). The foregoing could imply more recent convergence than that described to date. It also shows that MHC-DRB in primates has had a complex evolutionary mode in which trans-specific evolution has occurred at the same time as convergence between the different species analysed, underlining a predominantly intra-generic TSP pattern.

The molecular study in primates of the DRB gene in intron 2 (without considering the repeat sector) showed a high degree of identity for all the primates, indicating a clear division between NWM and OWM and between DRB gene lineages, demonstrating an independent origin for each DRB repertoire in Platyrrhini and Catarrhini. The study also verified that the microsatellite present in *A. nancymaae* and *A. vociferans* MHC-DRB gene intron 2 could be a useful marker for high and medium resolution genotyping of the MHC-DRB gene in these species, and probably in NWM. The microsatellite sequences could have been associated with the polymorphism observed for the corresponding *Aotus* MHC-DRB exon 2, making this a valuable tool for studying these genes' variability.

## Supporting Information

File S1 **Supporting tables and figure. Table S1. Sequences used for designing primers and analysis of exon 2+ intron 2.** Available genome sequences for the *Callithrix jaccus*, *Homo sapiens* and *Macaca mulatta* MHC-DRB region were used for designing the primers. Sequences used for comparative analysis of *Aotus* MHC-DRB exon 2+ intron 2 (partial), as well as those used for analysing MHC-DRB exon 2+ intron 2 (partial) in primates. **Table S2. Microsatellite sequence and length in Platyrrhini MHC-DRB.** STR structure corresponding to each DRB gene sequence for *A. nancymaae* and *A. vociferans*. The colours signify microsatellite identity or similarity and microsatellite sequences corresponding to MHC-DRB *Callithrix jacchus* (in bold) are shown at the end. **Figure S1. Aligning** ***A. vociferans*** **and** ***A. nancymaae*** **MHC-DRB gene exon 2+ intron 2 (partial) sequences.**
(PDF)
