# Supplementary material for: Characterising a Microsatellite for DRB Typing in Aotus vociferans and Aotus nancymaae (Platyrrhini)
Source: PLoS One. 2014 May 12;9(5):e96973. doi: 10.1371/journal.pone.0096973 (PMC4018467; doi:10.1371/journal.pone.0096973)
Supplement: File S1 — Supporting tables and figure. Table S1. Sequences used for designing primers and analysis of exon 2+ intron 2. Available genome sequences for the Callithrix jaccus, Homo sapiens and Macaca mulatta MHC-DRB region were used for designing the primers. Sequences used for comparative analysis of Aotus MHC-DRB exon 2+ intron 2 (partial), as well as those used for analysing MHC-DRB exon 2+ intron 2 (partial) in primates. Table S2. Microsatellite sequence and length in Platyrrhini MHC-DRB. STR structure corresponding to each DRB gene sequence for A. nancymaae and A. vociferans. The colours signify microsatellite identity or similarity and microsatellite sequences corresponding to MHC-DRB Callithrix jacchus (in bold) are shown at the end. Figure S1. Aligning A. vociferans and A. nancymaae MHC-DRB gene exon 2+ intron 2 (partial) sequences. (PDF) [file pone.0096973.s001.pdf]

Table S1. Sequences used in this article

Sequence	Remarks	Accesion Number Genbank
**Sequences used for primer designing**		
Caja_DRB_G_01	43380-44258 Callithrix jacchus BAC clone CH259-77F15 from chromosome unknown, complete sequence	AC242730
Caja_DRB_G_02	105908-107178 Callithrix jacchus BAC clone CH259-77F15 from chromosome unknown, complete sequence	AC242730
Caja_DRB_G_03	192613-193403 Callithrix jacchus BAC clone CH259-77F15 from chromosome unknown, complete sequence	AC242730
Caja_DRB_G_04	138963-138357 Callithrix jacchus BAC clone CH259-15O7 from chromosome unknown, complete sequence	AC243457
Caja_DRB_G_05	76772-75338 Callithrix jacchus BAC clone CH259-15O7 from chromosome unknown, complete sequence	AC243457
Caja_DRB_G_06	161835-162584 Callithrix jacchus BAC clone CH259-49P2 from chromosome unknown, complete sequence	AC242576
Mamu_DRB_G_01	140068-140822 Macaca mulatta Major Histocompatibility Complex BAC MMU012K22	AC148663
Mamu_DRB_G_02	28917-29606 Macaca mulatta Major Histocompatibility Complex BAC MMU012K22	AC148663
Mamu_DRB_G_03ps	111593-112214 Macaca mulatta Major Histocompatibility Complex BAC MMU012K22	AC148663
Mamu_DRB_G_05	c75283-74620 Macaca mulatta Major Histocompatibility Complex BAC MMU248N17	AC148697
Mamu_DRB_G_06	c151148-150454 Macaca mulatta Major Histocompatibility Complex BAC MMU248N17	AC148697
Mamu_DRB_G_07	c29018-28359 Macaca mulatta Major Histocompatibility Complex BAC MMU248N17	AC148697
Mamu_DRB_G_08	c26068-25409 Macaca mulatta Major Histocompatibility Complex BAC MMU281E18	AC148700
Mamu_DRB_G_04	173697-174407 Macaca mulatta Major Histocompatibility Complex BAC MMU370O02	AC148706
HLA_DRB1*040101	Homo sapiens major histocompatibility complex, class II, DR53 haplotype (DR53)	NG_002433
HLA_DRB1*070101	Human DNA sequence from clone DADB-102D14 on chromosome 6	CR753309
HLA_DRB1*1501	Homo sapiens major histocompatibility complex, class II, DR51 haplotype (DR51) on chromosome 6	NG_002432
HLA_DRB1*1602	Homo sapiens MHC class II antigen (HLA-DRB1) gene DR51	AB774985
HLA_DRB3*01012	Homo sapiens major histocompatibility complex, class II, DR52 haplotype (DR52) on chromosome 6	NG_002392
HLA_DRB3*020201	Human DNA sequence from clone DAQB-97F8 on chromosome 6	AL929581
HLA_DRB4*01030101	Homo sapiens major histocompatibility complex, class II, DR53 haplotype (DR53)	NG_002433
HLA_DRB5*0101	Homo sapiens major histocompatibility complex, class II, DR51 haplotype (DR51) on chromosome 6	NG_002432
HLA_DRB6ps	Homo sapiens major histocompatibility complex, class II, DR51 haplotype (DR51) on chromosome 6	NG_002432
HLA_DRB7ps	Homo sapiens major histocompatibility complex, class II, DR53 haplotype (DR53)	NG_002433
HLA_DRB1*03	c91958-91273 Human DNA sequence from clone DAQB-97F8 on chromosome 6	AL929581
**SEQUENCES HERE REPORTED**		
Aovo-DRB1*03:05	Aotus vociferans MHC class II antigen beta chain (Aovo-DRB) gene	KF447756
Aovo-DRB1*03:06	Aotus vociferans MHC class II antigen beta chain (Aovo-DRB) gene	KF447757
Aovo-DRB1*03:07	Aotus vociferans MHC class II antigen beta chain (Aovo-DRB) gene	KF447758
Aovo-DRB*W91:01	Aotus vociferans MHC class II antigen beta chain (Aovo-DRB) gene	KF447733
Aovo-DRB*W92:01	Aotus vociferans MHC class II antigen beta chain (Aovo-DRB) gene	KF447735
Aovo-DRB*W92:02	Aotus vociferans MHC class II antigen beta chain (Aovo-DRB) gene	KF447736
Aovo-DRB*W91:02	Aotus vociferans MHC class II antigen beta chain (Aovo-DRB) gene	KF447737
Aovo-DRB*W93:01	Aotus vociferans MHC class II antigen beta chain (Aovo-DRB) gene	KF447740
Aovo-DRB*W88:01	Aotus vociferans MHC class II antigen beta chain (Aovo-DRB) gene	KF447741
Aovo-DRB*W29:01	Aotus vociferans MHC class II antigen beta chain (Aovo-DRB) gene	KF447742
Aovo-DRB1*03:04	Aotus vociferans MHC class II antigen beta chain (Aovo-DRB) gene	KF447759
Aovo-DRB*W18:01	Aotus vociferans MHC class II antigen beta chain (Aovo-DRB) gene	KF447762
Aovo-DRB*W18:02	Aotus vociferans MHC class II antigen beta chain (Aovo-DRB) gene	KF447763
Aovo-DRB*W18:03	Aotus vociferans MHC class II antigen beta chain (Aovo-DRB) gene	KF447764
Aovo-DRB*W90:01	Aotus vociferans MHC class II antigen beta chain (Aovo-DRB) gene	KF447765
Aovo-DRB3*06:01	Aotus vociferans MHC class II antigen beta chain (Aovo-DRB) gene	KF447766
Aovo-DRB*W30:01	Aotus vociferans MHC class II antigen beta chain (Aovo-DRB) gene	KF447738
Aovo-DRB*W45:01	Aotus vociferans MHC class II antigen beta chain (Aovo-DRB) gene	KF447739
Aona-DRB*W91:01	Aotus nancymaae MHC class II antigen beta chain (Aona-DRB) gene	KF447734
Aona-DRB*W29:10	Aotus nancymaae MHC class II antigen beta chain (Aona-DRB) gene	KF447743
Aona-DRB*W29:08	Aotus nancymaae MHC class II antigen beta chain (Aona-DRB) gene	KF447744
Aona-DRB*W30:02	Aotus nancymaae MHC class II antigen beta chain (Aona-DRB) gene	KF447745
Aona-DRB1*03:28	Aotus nancymaae MHC class II antigen beta chain (Aona-DRB) gene	KF447746
Aona-DRB*W89:01	Aotus nancymaae MHC class II antigen beta chain (Aona-DRB) gene	KF447747
Aona-DRB3*06:25:01	Aotus nancymaae MHC class II antigen beta chain (Aona-DRB) gene	KF447748
Aona-DRB3*06:26	Aotus nancymaae MHC class II antigen beta chain (Aona-DRB) gene	KF447749
Aona-DRB3*06:27	Aotus nancymaae MHC class II antigen beta chain (Aona-DRB) gene	KF447750
Aona-DRB3*06:25:02	Aotus nancymaae MHC class II antigen beta chain (Aona-DRB) gene	KF447751
Aona-DRB3*06:15	Aotus nancymaae MHC class II antigen beta chain (Aona-DRB) gene	KF447752
Aona-DRB3*06:28	Aotus nancymaae MHC class II antigen beta chain (Aona-DRB) gene	KF447753
Aona-DRB1*03:29	Aotus nancymaae MHC class II antigen beta chain (Aona-DRB) gene	KF447754
Aona-DRB1*03:17:01	Aotus nancymaae MHC class II antigen beta chain (Aona-DRB) gene	KF447755
Aona-DRB*W18:08	Aotus nancymaae MHC class II antigen beta chain (Aona-DRB) gene	KF447760
Aona-DRB*W18:06	Aotus nancymaae MHC class II antigen beta chain (Aona-DRB) gene	KF447761

Sequence	Remarks	Accesion Number Genbank
**Sequences of Aotus MHC-DRB (molecular phylogenetic analysis exon 2)**		
Aoaz-DRB*W3801	Aotus azarai MHC class II antigen (Aoaz-DRB) gene Aoaz-DRB*W3801 allele	AY429143
Aoaz-DRB3*0601	Aotus azarai MHC class II antigen (Aoaz-DRB3) gene Aoaz-DRB3*0601 allele	AY429142
Aona-DRB*W1301	Aotus nancymaae MHC class II antigen beta chain (Aona-DRB) gene Aona-DRB*W1301 allele	AF132767
Aona-DRB*W1305	Aotus nancymaae isolate 20896_2 MHC class II antigen beta chain (DRB) mRNA DRB*W1305 allele	AY563223
Aona-DRB*W1306	Aotus nancymaae isolate 21100_1 MHC class II antigen beta chain (DRB) mRNA DRB*W1306 allele	AY563218
Aona-DRB*W1309	Aotus nancymaae isolate 22417-7 MHC class II antigen beta chain (DRB) mRNA DRB*W1309 allele	AY563255
Aona-DRB*W1312	Aotus nancymaae MHC class II antigen (DRB) mRNA DRB*W1312 allele	DQ162705
Aona-DRB*W1801	Aotus nancymaae MHC class II antigen beta chain (Aona-DRB) gene Aona-DRB*W1801 allele	AF132768
Aona-DRB*W1802	Aotus nancymaae MHC-DRB (DRB*W) mRNA DRB*W1802 allele	AF169487
Aona-DRB*W2901	Aotus nancymaae MHC class II antigen beta chain (Aona-DRB) gene Aona-DRB*W2901 allele	AF129806
Aona-DRB*W2906	Aotus nancymaae MHC class II antigen (DRB) mRNA DRB*W2906 allele	DQ162688
Aona-DRB*W2907	Aotus nancymaae isolate 20894_3 MHC class II antigen beta chain (DRB) mRNA DRB*W2907 allele	AY563201
Aona-DRB*W3001	Aotus nancymaae MHC class II antigen beta chain (Aona-DRB) gene Aona-DRB*W3001 allele	AF132766
Aona-DRB*W3801	Aotus nancymaae isolate 16606_7 MHC class II antigen beta chain (DRB) mRNA DRB*W3801 allele	AY563194
Aona-DRB*W4201	Aotus nancymaae isolate 20337_3 MHC class II antigen beta chain (DRB) mRNA DRB*W4201 allele	AY563209
Aona-DRB*W4401	Aotus nancymaae isolate 20559_2 MHC class II antigen beta chain (DRB) mRNA DRB*W4401 allele	AY563206
Aona-DRB*W4501	Aotus nancymaae isolate 20249_12 MHC class II antigen beta chain (DRB) mRNA DRB*W4501 allele	AY563180
Aona-DRB*W4701	Aotus nancymaae isolate 20465_3 MHC class II antigen beta chain (DRB) mRNA DRB*W4701 allele	AY563181
Aona-DRB*W4702	Aotus nancymaae isolate 22822_13 MHC class II antigen beta chain (DRB) mRNA DRB*W4702 allele	AY563183
Aona-DRB*W470404	Aotus nancymaae MHC class II antigen (DRB) mRNA DRB*W470404 allele	DQ162645
Aona-DRB1*0301	Aotus nancymaae MHC class II antigen beta chain (Aona-DRB1) gene Aona-DRB1*0301 allele	AF129793
Aona-DRB1*0302	Aotus nancymaae MHC class II antigen beta chain (Aona-DRB1) gene Aona-DRB1*0302 allele	AF129792
Aona-DRB1*0303	Aotus nancymaae MHC class II antigen beta chain (Aona-DRB1) gene Aona-DRB1*0303 allele	AF129794
Aona-DRB1*0305	Aotus nancymaae MHC class II antigen beta chain (Aona-DRB1) gene Aona-DRB1*0305 allele	AF129796
Aona-DRB1*0307	Aotus nancymaae MHC class II antigen beta chain (Aona-DRB1) gene Aona-DRB1*0307 allele	AF129798
Aona-DRB1*0313	Aotus nancymaae MHC class II antigen beta chain (Aona-DRB1) gene Aona-DRB1*0313 allele	AF132760
Aona-DRB1*0314	Aotus nancymaae MHC class II antigen beta chain (Aona-DRB1) gene Aona-DRB1*0314 allele	AF132761
Aona-DRB1*0319	Aotus nancymaae isolate 20719_2 MHC class II antigen beta chain (DRB1) mRNA DRB1*0319 allele	AY563188
Aona-DRB1*0324	Aotus nancymaae isolate 21955_10 MHC class II antigen beta chain (DRB1) mRNA DRB1*0324 allele	AY563193
Aona-DRB3*0601	Aotus nancymaae MHC class II antigen beta chain (Aona-DRB3) gene Aona-DRB3*0601 allele	AF129799
Aona-DRB3*0602	Aotus nancymaae MHC class II antigen beta chain (Aona-DRB3) gene Aona-DRB3*0602 allele	AF129800
Aona-DRB3*0603	Aotus nancymaae MHC class II antigen beta chain (Aona-DRB3) gene Aona-DRB3*0603 allele	AF129801
Aona-DRB3*0614	Aotus nancymaae isolate 20444_7 MHC class II antigen beta chain (DRB3) mRNA DRB3*0614 allele	AY563212
Aoni-DRB*W1301	Aotus nigriceps isolate 21921_5 MHC class II antigen beta chain (DRB) mRNA DRB*W1301 allele	AY563261
Aoni-DRB*W1303	Aotus nigriceps MHC class II antigen (DRB) mRNA DRB*W1303 allele	DQ162732
Aoni-DRB*W2901	Aotus nigriceps isolate 20596_8 MHC class II antigen beta chain (DRB) mRNA DRB*W2901 allele	AY563259
Aoni-DRB*W2902	Aotus nigriceps isolate 21919_18 MHC class II antigen beta chain (DRB) mRNA DRB*W2902 allele	AY563246
Aoni-DRB*W3801	Aotus nigriceps isolate 16584_5 MHC class II antigen beta chain (DRB) mRNA DRB*W3801 allele	AY563245
Aoni-DRB*W4201	Aotus nigriceps isolate 20483_1 MHC class II antigen beta chain (DRB) mRNA DRB*W4201 allele	AY563253
Aoni-DRB*W4301	Aotus nigriceps isolate 20848_16 MHC class II antigen beta chain (DRB) mRNA DRB*W4301 allele	AY563249
Aoni-DRB*W4401	Aotus nigriceps isolate 20596_4 MHC class II antigen beta chain (DRB) mRNA DRB*W4401 allele	AY563247
Aoni-DRB1*0301	Aotus nigriceps MHC class II antigen beta chain (Aoni-DRB1) gene Aoni-DRB1*0301 allele	AF129797
Aoni-DRB1*0304	Aotus nigriceps isolate 21791_15 MHC class II antigen beta chain (DRB1) mRNA DRB1*0304 allele	AY563242
Aoni-DRB1*0307	Aotus nigriceps MHC class II antigen (DRB1) mRNA DRB1*0307 allele	DQ162711
Aoni-DRB1*W1801	Aotus nigriceps isolate 20456_8 MHC class II antigen beta chain (DRB1) mRNA DRB1*W1801 allele	AY563257
Aoni-DRB3*0601	Aotus nigriceps isolate 20506_2 MHC class II antigen beta chain (DRB3) mRNA DRB3*0601 allele	AY563229
Aotr-DRB*W1801	Aotus trivirgatus MHC class II DRB*W1801 gene exon	L12477
Aotr-DRB1*0301	Aotus trivirgatus MHC class II DRB1*0301 gene exon	L12472
Aotr-DRB1*0303	Aotus trivirgatus partial DRB1 gene for MHC class II antigen DRB1*0303 allele exon 2	AJ544176
Aotr-DRB3*0602	Aotus trivirgatus partial DRB3 gene for MHC class II antigen DRB3*0602 allele exon 2	AJ544174
Aotr-DRB3*0603	Aotus trivirgatus partial DRB3 gene for MHC class II antigen DRB3*0603 allele exon 2	AJ544175
Aotr-DRB3*06	Aotus trivirgatus MHC class II DRB gene exon	L12474
Aovo-DRB*W130101	Aotus vociferans MHC class II antigen (DRB) mRNA DRB*W130101 allele	DQ162634
Aovo-DRB*W1303	Aotus vociferans isolate 20789_1 MHC class II antigen beta chain (DRB) mRNA DRB*W1303 allele	AY563258
Aovo-DRB*W4301	Aotus vociferans MHC class II antigen (DRB) mRNA DRB*W4301 allele	DQ162630
Aovo-DRB*W4701	Aotus vociferans isolate 16704_9 MHC class II antigen beta chain (DRB) mRNA DRB*W4701 allele	AY563227
Aovo-DRB1*0301	Aotus vociferans MHC class II antigen (DRB1) mRNA DRB1*0301 allele	DQ162628

Sequence	Remarks	Accesion Number Genbank
**Sequences of primates MHC-DRB (molecular phylogenetic analysis exon 2 + intron 2)**		
Caja_DRB_G_01	43380-44258 Callithrix jacchus BAC clone CH259-77F15 from chromosome unknown, complete sequence	AC242730
Caja_DRB_G_02	105908-107178 Callithrix jacchus BAC clone CH259-77F15 from chromosome unknown, complete sequence	AC242730
Caja_DRB_G_03	192613-193403 Callithrix jacchus BAC clone CH259-77F15 from chromosome unknown, complete sequence	AC242730
Caja_DRB_G_04	138963-138357 Callithrix jacchus BAC clone CH259-15O7 from chromosome unknown, complete sequence	AC243457
Caja_DRB_G_05	76772-75338 Callithrix jacchus BAC clone CH259-15O7 from chromosome unknown, complete sequence	AC243457
Caja_DRB_G_06	161835-162584 Callithrix jacchus BAC clone CH259-49P2 from chromosome unknown, complete sequence	AC242576
SAOE-DRB1*0303	Saguinus oedipus MHC class II antigen (SAOE-DRB1) pseudogene	AF173332
SAOE-DRB3*0501	Saguinus oedipus MHC class II antigen (SAOE-DRB3) pseudogene	AF173333
SAOE-DRB11*0102	Saguinus oedipus MHC class II antigen (SAOE-DRB11) gene	AF173334
SAOE-DRB11*0105	Saguinus oedipus MHC class II antigen (SAOE-DRB11) gene	AF173335
SAOE-DRB*W2209	Saguinus oedipus MHC class II antigen (SAOE-DRB) gene	AF173336
CAJA-DRB1*0304	Callithrix jacchus MHC class II antigen (CAJA-DRB1) gene	AF173337
CAMO-DRB1*0302	Callicebus moloch MHC class II antigen (CAMO-DRB1) gene	AF173338
CAMO-DRB3*0503	Callicebus moloch MHC class II antigen (CAMO-DRB3) gene	AF173339
CAMO-DRB3*0504	Callicebus moloch MHC class II antigen (CAMO-DRB3) pseudogene	AF173340
CEAP-DRB*W1301	Cebus apella MHC class II antigen (CEAP-DRB) gene	AF173341
CAJA-DRB*W1201	Callithrix jacchus MHC class II antigen (CAJA-DRB) gene	AF173348
Mamu_DRB_G_01	140068-140822 Macaca mulatta Major Histocompatibility Complex BAC MMU012K22	AC148663
Mamu_DRB_G_02	28917-29606 Macaca mulatta Major Histocompatibility Complex BAC MMU012K22	AC148663
Mamu_DRB_G_03ps	111593-112214 Macaca mulatta Major Histocompatibility Complex BAC MMU012K22	AC148663
Mamu_DRB_G_05	c75283-74620 Macaca mulatta Major Histocompatibility Complex BAC MMU248N17	AC148697
Mamu_DRB_G_06	c151148-150454 Macaca mulatta Major Histocompatibility Complex BAC MMU248N17	AC148697
Mamu_DRB_G_07	c29018-28359 Macaca mulatta Major Histocompatibility Complex BAC MMU248N17	AC148697
Mamu_DRB_G_08	c26068-25409 Macaca mulatta Major Histocompatibility Complex BAC MMU281E18	AC148700
Mamu_DRB_G_04	173697-174407 Macaca mulatta Major Histocompatibility Complex BAC MMU370O02	AC148706
Chae_DRB_G_01	63309-64000 Chlorocebus aethiops BAC clone CH252-249I23 from chromosome 6	AC241608
Chae_DRB_G_02	143590-144262 Chlorocebus aethiops BAC clone CH252-249I23 from chromosome 6	AC241608
Chae_DRB_G_03	111557-112224 Chlorocebus aethiops BAC clone CH252-249I23 from chromosome 6	AC241608
Chae_DRB_G_04	21256-21901 Chlorocebus aethiops BAC clone CH252-249I23 from chromosome 6	AC241608
MAFA-DRB*W3301	Macaca fascicularis MHC class II antigen (MAFA-DRB) gene	AF173349
MAAR-DRB1*0301	Macaca arctoides MHC class II antigen (MAAR-DRB1) gene	AF173350
MAAR-DRB1*0302	Macaca arctoides MHC class II antigen (MAAR-DRB1) gene	AF173351
MAAR-DRB1*0701	Macaca arctoides MHC class II antigen (MAAR-DRB1) gene	AF173352
MAFA-DRB*W301	Macaca fascicularis MHC class II antigen (MAFA-DRB) gene	AF173353
MAMU-DRB*W402	Macaca mulatta MHC class II antigen (MAMU-DRB) gene	AF173354
MAAR-DRB*W601	Macaca arctoides MHC class II antigen (MAAR-DRB) pseudogene	AF173355
MAAR-DRB5*0301	Macaca arctoides MHC class II antigen (MAAR-DRB5) gene	AF173356
MAMU-DRB6*0106	Macaca mulatta MHC class II antigen (MAMU-DRB6) pseudogene	AF173357
Mamu-DRB*W201	7681-8413 Macaca mulatta partial Mamu-DRB gene for MHC class II antigen	AM910410
Mamu-DRB*W305	7704-8400 Macaca mulatta partial Mamu-DRB gene for MHC class II antigen	AM910411
Mamu-DRB*W603	6501-7247 Macaca mulatta partial Mamu-DRB gene for MHC class II antigen	AM910412
Mamu-DRB*W2507	6540-7190 Macaca mulatta partial Mamu-DRB gene for MHC class II antigen	AM910413
Mamu-DRB1*0303	3087-3748 Macaca mulatta partial Mamu-DRB gene for MHC class II	AM910414
Mamu-DRB1*0306	Macaca mulatta partial Mamu-DRB1 gene for MHC class II antigen	AM910415
Mamu-DRB1*0309	7622-8327 Macaca mulatta partial Mamu-DRB1 gene for MHC class II antigen	AM910417
Mamu-DRB1*0404	2994-3688 Macaca mulatta partial Mamu-DRB1 gene for MHC class II antigen	AM910419
Mamu-DRB1*1007	8014-8684 Macaca mulatta partial Mamu-DRB1 gene for MHC class II antigen	AM910420
Mamu-DRB3*0405	Macaca mulatta partial Mamu-DRB3 gene for MHC class II antigen	AM910421
Mamu-DRB3*0408	2995-3667 Macaca mulatta partial Mamu-DRB3 gene for MHC class II antigen	AM910422
Mamu-DRB5*0301	976-8612 Macaca mulatta partial Mamu-DRB5 gene for MHC class II antigen	AM910423

Sequence	Remarks	Accesion Number Genbank
**Sequences of primates MHC-DRB (molecular phylogenetic analysis exon 2 + intron 2)**		
HLA_DRB1*010101	Homo sapiens HLA-DRB1 gene for MHC class II antigen	AM493435
HLA_DRB1*010201	Homo sapiens voucher Coriell Cell Repository DNA sample NA01018	AY663400
HLA_DRB1*040101	Homo sapiens major histocompatibility complex, class II, DR53 haplotype (DR53)	NG_002433
HLA_DRB1*0405	Homo sapiens MHC class II antigen (HLA-DRB1) gene, HLA-DRB1*0404	AB715390
HLA_DRB1*070101	Human DNA sequence from clone DADB-102D14 on chromosome 6	CR753309
HLA_DRB1*08:03:02	Homo sapiens HLA-DRB1 gene for MHC class II antigen	FN823238
HLA_DRB1*110101	Homo sapiens voucher Coriell Cell Repository DNA sample NA00576	AY663412
HLA_DRB1*110401	Homo sapiens voucher Coriell Cell Repository DNA sample NA14661	AY663394
HLA_DRB1*12:01:01	Homo sapiens HLA-DRB1 gene for major histocompatibility complex	AB715399
HLA_DRB1*130201	Homo sapiens voucher Coriell Cell Repository DNA sample NA14663	AY663413
HLA_DRB1*140101	Homo sapiens voucher Coriell Cell Repository DNA sample NA10540	AY663405
HLA_DRB1*140501	Homo sapiens voucher Coriell Cell Repository DNA sample NA04535	AY663408
HLA_DRB1*1501	Homo sapiens major histocompatibility complex, class II, DR51 haplotype (DR51) on chromosome 6	NG_002432
HLA_DRB1*16	Homo sapiens major histocompatibility complex, class II	NG_002432
HLA_DRB1*1602	Homo sapiens MHC class II antigen (HLA-DRB1) gene DR51	AB774985
HLA_DRB3*01012	Homo sapiens major histocompatibility complex, class II, DR52 haplotype (DR52) on chromosome 6	NG_002392
HLA_DRB3*020201	Human DNA sequence from clone DAQB-97F8 on chromosome 6	AL929581
HLA_DRB4*01030101	Homo sapiens major histocompatibility complex, class II, DR53 haplotype (DR53)	NG_002433
HLA_DRB5*0101	Homo sapiens major histocompatibility complex, class II, DR51 haplotype (DR51) on chromosome 6	NG_002432
HLA_DRB6ps	Homo sapiens major histocompatibility complex, class II, DR51 haplotype (DR51) on chromosome 6	NG_002432
HLA_DRB7ps	Homo sapiens major histocompatibility complex, class II, DR53 haplotype (DR53)	NG_002433
HLA_DRB1*10:01:01	Homo sapiens MHC class II antigen (HLA-DRB1) gene	JN157606
HLA_DRB1*15:02:01	Homo sapiens HLA-DRB1 gene for MHC class II antigen	AB774991
HLA_DRB1*03-AL929581	c91958-91273 Human DNA sequence from clone DAQB-97F8 on chromosome 6	AL929581
Patr-DRB1*020101	Pan troglodytes partial Patr-DRB1 gene for MHC class II antigen, Patr-DRB1*020101	AM910425
Patr-DRB3*0208	Pan troglodytes partial Patr-DRB3 gene for MHC class II antigen	AM910428
Patr-DRB1*10:01	Pan troglodytes verus partial patr-DRB1 gene for MHC class II antigen	HE800526
Gogo_DRB_01	44822-45507 Gorilla gorilla voucher Coriell Cell Repository DNA sample NG05251	AY663402
Patr-DRB*W902	Pan troglodytes partial Patr-DRB gene for MHC class II antigen	AM910424
Patr-DRB3*01020101	Pan troglodytes partial Patr-DRB3 gene for MHC class II antigen	AM910426
Patr_DRB_01	Pan troglodytes voucher Coriell Cell Repository DNA sample NS03646	AY663401
Gogo_DRB_02	c218276-217597 Gorilla DNA sequence from clone CH255-114D6, complete sequence	CU104652
Gogo_DRB_03	c218276-217597 Gorilla DNA sequence from clone CH255-114D6, complete sequence	CU104652
Gogo_DRB_04	73853067:151462-152106 Gorilla DNA sequence from clone CH255-351B13	CT025711
Gogo_DRB_05	73853067:151462-152106 Gorilla DNA sequence from clone CH255-351B13	CT025711
Patr-DRB4*02:01	5938-6579 Pan troglodytes troglodytes partial patr-DRB4	HE800525
Poab_DRB_01	200269-200926 Pongo abelii BAC clone CH276-191M9 from chromosome 6	AC206450
Patr_DRB_01	27628-28281 Pan troglodytes genomic DNA, chromosome 5	AP006503

Table S2. Microsatellite sequence and length in Platyrrhini MHC-DRB.

SEQUENCE	MICROSATELLITE			LENGTH	LZSize (Bytes)
	FIRST PART	CENTRAL PART	FINAL PART		
Aona DRB3*062501	CA(GA)4CT(GA)4CT(GA)4CC	(GA)5CACA(GA)2CACT(GA)4CA(GA)6CT(GA)4CAGT((GA)3,3,4CT)(GA)4CACT(GA)4CACAGT(GA)2CA(GA)2AACA(GA)2AAGACT(GA)3TACACT(GA)4CA(GA)2CT(GA)3CA(GA)3CACA(GA)3CA(GA)3CTGAAAGACT(GA)4CA((GA)3,4CT)(GA)3AAGACT((GA)4,3CT)(GA)3AACA((GA)2,3CT)(GA)3CACT(GA)3CT(GA)3CACAGATA(GA)2TT((GA)3,5CT)(GA)3CACTGATA((GA)2,5,3CT)	(GA)15CT(GA)3GCGCCTTG	314	4333
Aovo DRB3*0601	CA(GA)4CT(GA)4CT(GA)4CC	(GA)5(CA)2(GA)2CACT(GA)4CAGAG(GA)4CT(GA)4CAGT((GA)3,3,4CT)(GA)4CACT(GA)4CACAGT(GA)2CA(GA)2AACA(GA)2AAGACT(GA)3TACACT(GA)4CA(GA)2CT(GA)3CA(GA)3CACA(GA)3CA(GA)3CTGAAAGACT(GA)4CA((GA)3,4CT)(GA)3AAGACT((GA)4,3)(GA)3AACA((GA)2,3CT)(GA)3CACT(GA)3CT(GA)3CACAGATA(GA)2TT((GA)3,5CT)(GA)3CACTGATA((GA)2,5,3CT)	(GA)15CT(GA)3GCGCCTTG	310	4343
Aona DRB3*062502	CA(GA)4CT(GA)4CC(GA)4CC	(GA)5CACA(GA)2CACT(GA)4CA(GA)6CT(GA)4CAGT((GA)3,3,4CT)(GA)4CACT(GA)4CACAGT(GA)2CA(GA)2AACA(GA)2AAGACT(GA)3TACACT(GA)4CA(GA)2CT((GA)3,3CA)CA(GA)3CA(GA)3CTGAAAGACT(GA)4CA((GA)3,4CT)GAAAGACT((GA)4,3CT)(GA)3AACA((GA)2,3CT)(GA)3CACT(GA)3CT(GA)3CACAGATA(GA)2TT((GA)3,5CT)(GA)3CACTGATA(GA)2CT	(GA)16CT(GA)3GCGCCTTG	294	4310
Aona DRB3*0626	CA(GA)4CT(GA)4CT(GA)4CC	(GA)5CACA(GA)2CACT(GA)4CA(GA)5CT(GA)4CAGT((GA)3,3,4CT)(GA)4CACT(GA)4CACAGT(GA)2CA(GA)2AACA(GA)2AAGACT(GA)3TACACT(GA)4CA(GA)2CT((GA)3,3CA)CA(GA)3CA(GA)3CTGAAAGACT(GA)4CA((GA)3,4CT)(GA)3AAGACT((GA)4,3CT)(GA)3AACA((GA)2,3CT)(GA)3CACT(GA)3CT(GA)3CACAGATA(GA)2TT((GA)3,5CT)(GA)3CACTGATA((GA)2,5,3CT)	(GA)15CT(GA)3GCGCCTTG	312	4357
Aona DRB3*0628	CA(GA)4CT(GA)4CT(GA)4CC	(GA)5CACA(GA)2CACT(GA)4CA(GA)6CT(GA)4CAGT((GA)3,3,4CT)(GA)4CACT(GA)4CAGAG((GA)2,3CA)(GA)2AAGACT(GA)3CA(GA)2CT(GA)3TACACT(GA)4CA(GA)2CT((GA)3,3CA)CT(GA)4CA(GA)3CTGAAAGAT(GA)4CA((GA)4,5,3CT)(GA)3TACTTAGG(GA)2CT(GA)4CA(GA)3CT(GA)3AAGACT((GA)4,3,3CT)(GA)3AACA((GA)2,3CT)(GA)3CACT(GA)3CT(GA)3CACAGATA(GA)2TT(GA)3CT(GA)3CACTGATA((GA)5,3)	(GA)11AACT(GA)3GCACTTG	346	4855
Aona DRB3*0627	CA(GA)4CT(GA)4CT(GA)4CC	(GA)5CACA(GA)2CACT(GA)4CA(GA)6CT(GA)4CAGT((GA)3,3,4CT)(GA)4CACT(GA)4CAGAG((GA)2,3CA)(GA)2AAGACT(GA)3CA(GA)4CT(GA)3TACACT(GA)4CA(GA)2CT((GA)3,3CA)CT(GA)4CA(GA)3CTGAAAGACTCA(GA)4CA(GA)4CT(GA)4GGCT(GA)3CT(GA)3TACTTAGG(GA)2CT(GA)4CA(GA)3CT(GA)3AAGACT((GA)4,3,3CT)(GA)3AACA((GA)2,3CT)(GA)3CACT(GA)3CT(GA)3CACAGATA(GA)2TT(GA)3CT(GA)3CACTGATA((GA)2,5,3CT)	(GA)10AACT(GA)3GCACTTG	350	4951
Aona DRB3*0615	CA(GA)4CT(GA)4CT(GA)4CC	(GA)5CACA(GA)2CACT(GA)4CA(GA)6CT(GA)4CAGT((GA)3,3,4CT)(GA)4CACT(GA)4CACAGT(GA)2CA(GA)2AACA(GA)2AAGACT(GA)3TACACT(GA)4CA(GA)2CT((GA)3,3CA)CA(GA)3CA(GA)3CTGAAAGACT(GA)4CA((GA)3,4CT)(GA)3AAGACT((GA)4,3CT)(GA)3AACA((GA)2,3CT)(GA)3CACT(GA)3CT(GA)3CACAGATA(GA)2TT((GA)3,5CT)(GA)3CACTGATA((GA)2,5,3CT)	(GA)15CT(GA)3GCGCCTTG	314	4333
Aona DRB1*0329GA	CA(GA)4CTGAAA(GA)2CT(GA)4CACTGAGT	(GA)2GT((GA)2,3CA)(GA)2AAGACT(GACA)2(GA)4CT(GA)2GGTACACT(GA)4CAGAGT((GA)2,3CA)GAGTAAGACTGACA(GA)4CT(GA)3TACACT(GA)4CA(GA)2CT((GA)3,3CA)CT(GA)4CA(GA)3CTGAAAGACT(GA)4CA((GA)4,5,3CT)(GA)3TACTTAGG(GA)2CT(GA)4CA(GA)3CT(GA)2CAGACT(GA)3AAGACT(GA)6CT(GA)3GGCT(GA)5CTGAGG(GA)2CA(GA)2AT(GA)5CT(GA)3CAAGACA(GA)2CT(GA)4T(GA)2CA((GA)4,4CT)AA(GA)3CTGAGG((GA)2,4CT)GAAAGACT(GA)5CT(GA)4CAAGACA(GA)2CT(GA)4T(GA)2CA(GA)4CT(GA)3AAGACT(GA)3CA((GA)2,3,2CT)(GA)3AATAGACT(GA)3CT(GA)3CACT(GA)3CT(GA)3CACAGATA(GA)2TT((GA)3,5CT)(GA)3CACTGATA((GA)3,5,3CT)	(GA)23CT(GA)3GCGCCGTG	446	7249
Aona DRB1*031701	CA(GA)4CT(GA)4CT(GA)4CACT	(GA)4GT((GA)2,3CA)(GA)2AAGACTGACAGACA(GA)4CT(GA)2GGTACACT(GA)4CAGAGT((GA)2,3CA)GAGTAAGACTGACA(GA)4CT(GA)3TACACT(GA)4CA(GA)2CT(GA)3CAGC(GA)2CACT(GA)4CA(GA)3CTGAAAGACT(GA)4CA((GA)4,5,3CT)(GA)3TACTTAGG(GA)2CT(GA)4CA(GA)3CT(GA)2CAGACT(GA)3AAGACT(GA)6CT(GA)3GGCT(GA)5CTGAGG(GA)2CC(GA)2AT(GA)5CT(GA)3CAAGACA(GA)2CT(GA)4T(GA)2CA((GA)4,4CT)AA(GA)3CTGAGG((GA)2,4CT)GAAAGACT(GA)5CT(GA)4CAAGACA(GA)2CT(GA)4T(GA)2CA(GA)4CT(GA)3AAGACT(GA)3CA((GA)2,3,2CT)(GA)3AATAGACT(GA)3CT(GA)3CACT(GA)3CT(GA)3CACAGATA(GA)2TT((GA)3,5CT)(GA)3CACTGATA ((GA)2,5,3CT)	(GA)25CT(GA)3GCGCCGTG	462	7205

Aovo DRB1*0304	CA(GA)4CT(GA)4CC(GA)5CACA	(GA)2CACT(GA)4CA(GA)6CT(GA)4CAGT((GA)3,3CT)(GA)4CACT(GA)4CAGAGT(GA)2CA(GA)2AACA(GA)2AAGACT(GA)3TACACT(GA)4CA(GA)2CT((GA)3,3CA)CT(GA)4CA(GA)3CTGAAAGACT(GA)4CA(GA)3CT(GA)3AAGACT((GA)4,3CT)(GA)3AACA((GA)2,3CT)(GA)3CACT(GA)3CT(GA)3CACAGATA(GA)2TT(GA)3CT(GA)4CACTGATA((GA)2,4,3CT)	(GA)11AACT(GA)3GCACTTG	274	3949
Aovo DRB1*0305	CA(GA)4CT(GA)4CC(GA)5CACA	(GA)2CACT(GA)4CA(GA)6CT(GA)4CAGT((GA)3,3,2CT)(GA)4CACT(GA)4CAGAGG((GA)2,3CA)(GA)2AAGACT(GA)3CA(GA)3CT(GA)3TACACT(GA)4CA(GA)2CT(GA)3CAGG(GA)2CACT(GA)4CA(GA)3CTGAAAGACT(GA)4CA(GA)4CT((GA)5,3CT)(GA)3TACTTAGG(GA)2CT(GA)4CA(GA)3CT(GA)3AAGACTCT(GA)4CT(GA)3AACAGGGACT(GA)3CT(GA)3CACT(GA)3CT(GA)3CACAGATAGAAATT(GA)3CT(GA)3CACTGATA ((GA)2,5,3CT)(GA)3CA(GA)2CT	(GA)9AACT(GA)3GCACTTG	336	4282
Aovo DRB1*0306	CA(GA)4CT(GA)4CC(GA)5CACA	(GA)2CACT(GA)4CA(GA)6CT(GA)4CAGT((GA)3,3CT)(GA)4CT(GA)4CACT(GA)4CAGAGT(GA)2CA(GA)2AACA(GA)2AAGACT(GA)2AATACACT(GA)4CA(GA)2CT((GA)3,3CA)CT(GA)4CA(GA)3CTGAAAGACT(GA)4CA(GA)3CT(GA)3AAGACT((GA)4,3CT)(GA)3AACA((GA)2,3CT)(GA)3CACT(GA)3CT(GA)3CACAGATA(GA)2TT(GA)3CT(GA)3CACTGATA((GA)2,4,3CT)	(GA)13AACT(GA)3GCACTTG	282	4102
Aovo DRB1*0307	CA(GA)4CT(GA)4CC(GA)7CACA	(GA)2CACT(GA)4CA(GA)6CT(GA)4CAGT((GA)3,3,4CT)(GA)4CACT(GA)4CAGAGT(GA)2CA(GA)2AACA(GA)2AAGACT(GA)2AATACACT(GA)4CA(GA)2CT((GA)3,3CA)CT(GA)4CA(GA)3CTGAAAGACT(GA)4CA(GA)3CT(GA)3AAGACT((GA)4,3CT)(GA)3AACA((GA)2,3CT)(GA)3CACT(GA)3CT(GA)3CACAGATA(GA)2TT(GA)3CT(GA)3CACTGATA((GA)2,4,3CT)	(GA)13AACT(GA)3GCACTTG	286	4096
Aona DRB*W8901	CA(GA)2CA(GA)2CT(GA)4CT	(GA)7AT((GA)4,5,4CT)(GA)3TA(GA)2CTGAAAGAC(GA)2CA(GA)3AACT(GA)3AACATA(GA)2CT(GA)5CACA	(GA)5CAGAGC(GA)4GCG	114	1833
Aona DRB*W1808	CA(GA)5CT(GA)4CT(GA)10CACA	(GA)4TAGACA(GA)2CA((GA)3,3CT)GT((GA)4,4CT)GTGCAAGACC((GA)5,2CT)(GA)3TT(GA)4CA(GA)2CT(GA)3GGCT(GA)3CTCG(GA)3CTGAGC(GA)3CT(GA)2TAGACT(GA)3AACT(GA)3TACT	(GA)6(GC)3GACAGCG	168	2893
Aona DRB*W1806	CA(GA)5CT(GA)4CT(GA)10CACA	(GA)4TAGACA(GA)2CA((GA)3,3CT)GT(GA)4CTGTGCAAGACC((GA)5,2CT)(GA)3TT(GA)4CA((GA)2,4CT)(GA)3CTCG(GA)3CTGAGC(GA)3CT(GA)2TAGACT(GA)3AACT(GA)3TACT	(GA)6(GC)3GACAGCG	162	2729
Aovo DRB*W1803	CA(GA)5CT(GA)15CACA	(GA)4TAGACA(GA)2CA((GA)3,3CT)GT(GA)4CT(GA)4CTGTGCAAGACC((GA)5,2,4CT)(GA)3CTCG(GA)3CTGAGC(GA)3CT(GA)2TAGACT(GA)3AACT(GA)3TACT	(GA)6(GC)3GACAGCG	154	2479
Aovo DRB*W1801	CA(GA)7CACA	(GA)4TAGACA(GA)2CA((GA)3,3CT)GT((GA)4,4CT)GTGCAAGACC(GA)5TT(GA)2CT(GA)2TT(GA)4CA((GA)2,4CT)(GA)3CTCG(GA)3CTGAGC(GA)3CT(GA)2TAGACT(GA)3AACT(GA)3TACT	(GA)6(GC)3GACAGCG	144	2591
Aovo DRB*W1802	CA(GA)5CT(GA)4CT(GA)13CACA	(GA)4TAGACA((GA)2,3CT)GT(GA)4CTGTGCAAGACC((GA)5,2CT)(GA)3TT(GA)4CA((GA)2,4CT)(GA)3CTCG(GA)3CTGAGC(GA)3CT(GA)2TAGACT(GA)3AACA(GA)2TACT	(GA)6(GC)3GACAGCG	160	2663
Aona DRB1*0328	CA(GA)4CT(GA)4CT(GA)4CC	(GA)5CACA(GA)2CACT(GA)4CA(GA)6CT(GA)4CAGT((GA)3,3,2CT)(GA)4CACT(GA)4CAGAGT((GA)2,3CA)(GA)2AAGGCTGACAGACA(GA)4CT(GA)3TACACT(GA)4CA(GA)2CT(GA)3CAGACACTCAGACACT((GA)4,3CT)GT(GA)5CT(GA)4CA(GA)4CT(GA)4CACT(GA)4CA(GA)3CTGAAAGACT(GA)3TACTTAGG(GA)2CT(GA)3AACA((GA)3,4CT)(GA)3AAGACT(GA)5CT(GA)3GGCT(GA)3CTGAGG(GA)2CA(GA)2AT(GA)5CT(GA)3TAAGACA(GA)2CT(GA)4T(GA)2CA(GA)4CT(GA)3AAGACT(GA)3CA((GA)3,3CT)(GA)4CA((GA)2,3,3CT)(GA)3CACAGATA(GA)2TT((GA)3,5CT)GGGGGGG	(GA)11CT(GA)3GCACGTG	414	6113

Aovo DRB*W4501	CA(GA)2CA(GA)2CT(GA)4CT	(GA)3CAGACT(GA)5GT(GA)12G((GA)2,4CA)(GA)2GGCT(GA)3GCCA(GA)3CT(GA)2AAGACA	(GA)2GCCAGAGCCA(GA)3GCA	98	1593
Aovo DRB*W8701	CA(GA)4CT	(GA)6CT(GA)5CT(GA)15CT	(GA)3GCGCGTG	66	734
Aona DRB*W2910	CA(GA)2CA(GA)2CT(GA)4CT	(GA)7AT((GA)4,5,4CT)(GA)3TA(GA)2CTGAAAGAC(GA)2CA(GA)3AACT(GA)3AACATA(GA)2CT(GA)5CACA	(GA)5CAGAGC(GA)4GCG	114	1833
Aona DRB*W2908	CA(GA)2CA(GA)2CT(GA)4CT	(GA)7AT((GA)4,5,4CT)(GA)3TA(GA)2CTGAAAGAC(GA)2CA(GA)3AACT(GA)3AACATA(GA)2CT(GA)5CACA	(GA)5CAGAGC(GA)4GCG	114	1833
Aovo DRB*W2901	CA(GA)2CA(GA)2CT(GA)4CT	(GA)7ATGAAA((GA)2,5,4CT)(GA)3TA(GA)2CTGAAAGAC(GA)2CA(GA)4CT(GA)3AACATA(GA)2CT(GA)5CACA	(GA)5CAGAGC(GA)4GCG	112	1854
Aovo DRB*W9001	CA(GA)2CA(GA)2CT(GA)4CT	(GA)4AT((GA)4,4CT)(GA)3TA(GA)2CT(GA)3CAGACACA	(GA)5CAGAGC(GA)4GCG	74	1182
Aovo DRB*W8801	CA(GA)4CA(GA)7CT(GA)7CT	(GA)7CT(GA)4AT((GA)4,5,4,6CT)(GA)6CTGAAAGAC(GA)2CA(GA)4CT(GA)3AACA(GA)3CT(GA)3GGGACACA	(GA)5CAGAGC(GA)4GCG	156	1975
Aovo DRB*W8501	CA(GA)2CA(GA)3CC(GA)5CT	(GA)6CTGAAAGAC(GA)2CA(GA)4CT(GA)3GGGACACA	(GA)5CAGAGC(GA)4GCG	68	1244
Aovo DRB*W8502	CA(GA)2CA(GA)3CC	(GA)3GCGACT((GA)4,6CT)(GA)6CTGAAAGAC(GA)2CA(GA)4CT(GA)3GGGACACA	(GA)5CAGAGC(GA)4GCG	84	1477
Aona DRB*W8501	CA(GA)2CA(GA)3CC	((GA)5,4CT)GC((GA)5,6CT)GAAAGAC(GA)2CA(GA)4CT(GA)3GGGACACACACA	(GA)5CAGAGC(GA)4GCG	90	1443
Aovo DRB*W8601	CA(GA)2CA(GA)2CA	(GA)10AA(GA)7AGACA(GA)14G((GA)2,4CA)((GA)3,3CT)(GA)2AAGACA(GA)3GCCA	(GA)2GCCAGAGCCA(GA)3GCA	114	1528
Aovo DRB*W8602	CA(GA)2CA(GA)2CA	(GA)9AA(GA)7AGACA(GA)15G((GA)2,4CA)(GA)3CT(GA)2AAGACA(GA)3GCCA	(GA)2GCCAGAGCCA(GA)2GCA	106	1513

Aovo DRB*W3001	CA(GA)2CA(GA)2CA	(GA)10AA(GA)7AGACA(GA)15G((GA)2,4CA)((GA)3,3CT)(GA)2AAGACA(GA)3GCCA	(GA)2GCCAGAGCCA(GA)3GCA	116	1527
Aona DRB*W3002	CA(GA)2CA(GA)2CA(GA)9AA	(GA)11AA(GA)7AGACA(GA)9GTG((GA)2,4CA)(GA)3GCCA(GA)3CT(GA)2AAGACA	(GA)2GCCAGAGCCA(GA)3GCA	118	1623
**Caja-DRB03**	**CA(GA)3CA(GA)2TACA**	**(GA)4CT(GA)3GG(GA)2CA(GA)2CT(GA)4CA(GA)9CA(GA)2TACA(GA)4CT(GA)4CA(GA)2GC(GA)2GCCA(GA)3AA**	**(GA)3GGAAA(GA)30GGGCG**	**158**	
**Caja-DRB06**	**CA(GA)3CA(GA)2TACA**	**(GA)4CT(GA)4CA(GA)2GC(GA)2GC(GA)2GC(GA)2GCCA**	**(GA)7GGAAA(GA)37GGGCG**	**130**	
**Caja-DRB02**	**CA(GA)11CA(GA)9CA**	**(GA)10TTGACA(GA)2CA(GA)3CT(GA)3CGAGGCAAAGACT(GA)2CAGACGAGAAG(GA)4CA(GA)2TT(GA)3AAGACT(GA)3CA(GA)4CT(GA)2AACT(GA)4CA(GA)2CT(GA)2CACT(GA)3CT(GA)3CC(GA)4C(GA)2CA(GA)4TA(GA)2AAGACA(GA)4CT(GA)3CA(GA)2CAGACT(GA)4CT(GA)2CT(GA)4CTTA(GA)2CT(GA)3CTGAAAGACT(GA)5CT(GA)3CACT(GA)4CT(GA)2GC(GA)2CA(GA)3CT(GA)5CCGAGG(GA)2CT(GA)2CAGAAACT(GA)3CT(GA)2AAGACT(GA)3CTAA(GA)2TACT(GA)5CT(GA)2CACTGATA(GA)2CT(GA)4CA(GA)2CT(GA)4TA(GA)4CTGAGCGACTAA(GA)2CACT(GA)3CT(GA)2CACTCATA(GA)2CT(GA)3CT(GA)2GGGACT(GA)3CTGACAGACACTGATA(GA)2CTCA(GA)4CT(GA)3CACT(GA)4CT**	**(GA)2GCCAGAGTGACACT(GA)16GCG**	**434**	
**Caja-DRB05**	**CA(GA)34CA(GA)13TTGACA**	**(GA)2CT(GA)3CT(GA)3CGAGGCAAAGACT(GA)2CAGAC(GA)2AG(GA)4CA(GA)2TT(GA)3AAGACT(GA)3CA(GA)4CT(GA)2AACT(GA)4CA(GA)2CT(GA)2CACT(GA)3CT(GA)3CTGAAAGTAAGACT(GA)5CT(GA)2GT(GA)2CA(GA)3CA(GA)3GCCT(GA)3CAGACA(GA)2CA(GA)4CT(GA)2CT(GA)5TT(GA)4CT(GA)3CACT(GA)7CT(GA)3CC(GA)4C(GA)2CA(GA)3GATAAAGACA(GA)4CT(GA)3CA(GA)2CAGACT(GA)4CT(GA)2CT(GA)4CTTA(GA)2CT(GA)3CTGAAAGACT(GA)3CACT(GA)4CT(GA)2GC(GA)2CA(GA)3CT(GA)5CCGAGG(GA)2CT(GA)2CAGAAACT(GA)3CT(GA)4CT(GA)3CTAA(GA)2TACT(GA)5CT(GA)2CACTGATA(GA)2CT(GA)4CA(GA)2CT(GA)4TA(GA)4CT(GA)3CTAA(GA)2CACT(GA)3CT(GA)2CACTCATA(GA)2CT(GA)2GGGACT(GA)3CTGACAGACACTGATA(GA)2CTCA(GA)4CT(GA)3CACT(GA)4CT**	**(GA)2GCCAGAGTGACACT(GA)15(GCGA)2GCGCAAGCG**	**554**	
**Caja-DRB01**	**(GA)16CA(GA)9CT(GACA)2CT**	**(GA)5CT(GA)3GTCTCA(GA)2CT(GA)4CA(GA)2CT(GA)3CTGT(GA)5CT(GA)4C(GA)2GG(GA)2(CAGA)2(GA)2AAGACT(GA)2CT(GA)5CT(GA)4CTGAAAGACT(GA)3CT(GA)3CAAGACA(GA)2CT(GA)4TAAGACAGACT(GA)4CTGATG(GA)4CT(GA)9GG**	**(GA)2AAGG(GA)3GGGCG**	**212**	

Figure S1. *Aotus* MHC-DRB Exon 2 + Intron 2 (partial)

```
                             *        20         *        40         *        60
                   TTCGTGTCCCCACAGCACGTTTCtTg   C G  TA g  TGAGTGT ATTTC TCAAcGGGACGGAGC
Aona-DRB1*0328GB   : TTCGTGTCCCCACAGCACGTTTCTTGTTCCAGACTACGTCTGAGTGTCATTTCTTCAACGGGACGGAGC :   69
Aona-DRB1*0329GA   : TTCGTGTCCCCACAGCACGTTTCTTGTTCCAGACTACGTCTGAGTGTCATTTCTTCAACGGGACGGAGC :   69
Aona-DRB1*031701GA : TTCGTGTCCCCACAGCACGTTTCTTGTTCCAGACTACGTCTGAGTGTCATTTCTTCAACGGGACGGAGC :   69
Aovo-DRB1*0304GA   : TTCGTGTCCCCACAGCACGTTTCTTGTTCCAGACTACGTCTGAGTGTCATTTCTTCAACGGGACGGAGC :   69
Aovo-DRB1*0305GA   : TTCGTGTCCCCACAGCACGTTTCTTGTTCCAGACTACGTCTGAGTGTCATTTCTTCAACGGGACGGAGC :   69
Aovo-DRB1*0306GA   : TTCGTGTCCCCACAGCACGTTTCTTGTTCCAGACTACGTCTGAGTGTTATTTCTTCAACGGGACGGAGC :   69
Aovo-DRB1*0307GA   : TTCGTGTCCCCACAGCACGTTTCTTGTTCCAGACTACGTCTGAGTGTTATTTCTTCAACGGGACGGAGC :   69
Aona-DRB3*0615     : TTCGTGTCCCCACAGCACGTTTCTTGGAGCTGGTTAAGCATGAGTGTCATTTCTTCAACGGGACGGAGC :   69
Aona-DRB3*0627     : TTCGTGTCCCCACAGCACGTTTCTTGGAGCTGGTTAAGCATGAGTGTCATTTCTTCAACGGGACGGAGC :   69
Aona-DRB3*062501   : TTCGTGTCCCCACAGCACGTTTCTTGGAGCTGGTTAAGCATGAGTGTCATTTCTTCAACGGGACGGAGC :   69
Aona-DRB3*0626     : TTCGTGTCCCCACAGCACGTTTCTTTGAGCTGGTTAAGCATGAGTGTCATTTCTTCAACGGGACGGAGC :   69
Aona-DRB3*062502   : TTCGTGTCCCCACAGCACGTTTCTTGGAGCTGGTTAAGCATGAGTGTCATTTCTTCAACGGGACGGAGC :   69
Aona-DRB3*0628     : TTCGTGTCCCCACAGCACGTTTCTTGGAGCTGGTTAAGCATGAGTGTCATTTCTTCAACGGGACGGAGC :   69
Aovo-DRB3*0601     : TTCGTGTCCCCACAGCACGTTTCTTGGAGCTGGTTAAGCATGAGTGTCATTTCTTCAACGGGACGGAGC :   69
Aona-DRB*W8901     : TTCGTGTCCCCACAGCACGTTTCTTGGAGCAGACTAAGAGTGAGTGTCATTTCCTCAACGGGACGGAGC :   69
Aona-DRB*W1808     : TTCGTGTCCCCACAGCACGTTTCTTGGAGCTGGTTAAGTCTGAGTGTTATTTCTTCAACGGGACGGAGC :   69
Aona-DRB*W1806     : TTCGTGTCCCCACAGCACGTTTCTTGGAGCAGGTTAAGTCTGAGTGTTATTTCTTCAACGGGACGGAGC :   69
Aovo-DRB*W1801     : TTCGTGTCCCCACAGCACGTTTCTTGGAGCAGGCTAAGTCTGAGTGTTATTTCTTCAACGGGACGGAGC :   69
Aovo-DRB*W1802     : TTCGTGTCCCCACAGCACGTTTCTTGGAGCAGGCTAAGTCTGAGTGTTATTTCTTCAACGGGACGGAGC :   69
Aovo-DRB*W1803     : TTCGTGTCCCCACAGCACGTTTCTTGGAGCAGGGTAAATCTGAGTGTCATTTCTTCAACGGGACGGAGC :   69
Aovo-DRB*W8801     : TTCGTGTCCCCACAGCACGTTTCTTGGAACAGGTTAAGGATGAGTGTCATTTCCTCAACGGGACGGAGC :   69
Aovo-DRB*W2901     : TTCGTGTCCCCACAGCACGTTTCTTGGAGCAGACTAAGAGTGAGTGTCATTTCCTCAACGGGACGGAGC :   69
Aona-DRB*W2908     : TTCGTGTCCCCACAGCACGTTTCTTGGAGCAGACTAAGAGTGAGTGTCATTTCCTCAACGGGACGGAGC :   69
Aona-DRB*W2910     : TTCGTGTCCCCACAGCACGTTTCTTGGAGCAGACTAAGAGTGAGTGTCATTTCCTCAACGGGACGGAGC :   69
Aovo-DRB*W3001     : TTCGTGTCCCCACAGCACGTTTCCTGGAGCAGGTTAAGTATGAGTGTCATTTCCTCAACGGGACGGAGC :   69
Aona-DRB*W3002     : TTCGTGTCCCCACAGCACGTTTCTTGGAGCAGGTTAAGTATGAGTGTCATTTCCTCAACGGGACGGAGC :   69
Aovo-DRB*W9201     : TTCGTGTCCCCACAGCACGTTTCTTGTTCCAGACTACGTCTGAGTGTTATTTCTTCAACGGGACGGAGC :   69
Aovo-DRB*W9202     : TTCGTGTCCCCACAGCACGTTTCTTGTTCCAGACTACGTCTGAGTGTTATTTCTTCAACGGGACGGAGC :   69
Aona-DRB*W9101     : TTCGTGTCCCCACAGCACGTTTCTTGGAGCAGGCTAAGTGTGAGTGTCATTTCCTCAACGGGACGGAGC :   69
Aovo-DRB*W9101     : TTCGTGTCCCCACAGCACGTTTCTTGGAGCAGGCTAAGTGTGAGTGTCATTTCCTCAACGGGACGGAGC :   69
Aovo-DRB*W9102     : TTCGTGTCCCCACAGCACGTTTCTTGGAGCAGGCTAAGGGTGAGTGTCATTTCCTCAACGGGACGGAGC :   69
Aovo-DRB*W9001     : TTCGTGTCCCCACAGCACGTTTCTTGGAGCAGGGTAAGTCTGAGTGTCATTTCCTCAACGGGACGGAGC :   69
Aovo-DRB*W4501     : TTCGTGTCCCCACAGCACGTTTCTTGGAGCAGGTTAAGCATGAGTGTCATTTCTTCAACGGGACGGAGC :   69
Aovo-DRB*W9301     : TTCGTGTCCCCACAGCACGTTTCTTGGAGCTGATTAAGTTTGAGTGTCATTTCTTCAATGGGACGGAGC :   69

                   *        80         *        100         *        120         *        1
                   GGGTGCgGt cCTGgA AGAtactT tATAACCaGgAGGAG   gtGCGCTTCgACAGCGACGTGGGGG
Aona-DRB1*0328GB   : GGGTGCGGTTCCTGGACAGATACTTCTATAACCAGGAGGAGTATGTGCGCTTCGACAGCGACGTGGGGG :  138
Aona-DRB1*0329GA   : GGGTGCGGTACCTGGACAGATACTTTTATAACCAGGAGGAGTATGTGCGCTTCGACAGCGACGTGGGGG :  138
Aona-DRB1*031701GA : GGGTGCGGTACCTGGACAGATACTTTTATAACCAGGAGGAGTATGTGCGCTTCGACAGCGACGTGGGGG :  138
Aovo-DRB1*0304GA   : GGGTGCGGTACCTGGACAGATACTTCTATAACCAGAAGGAGTATGTGCGCTTCGACAGCGACGTGGGGG :  138
Aovo-DRB1*0305GA   : GGGTGCGGTACCTGGACAGATACTTCTATAACCAGGAGGAGTTCGTGCGCTTCGACAGCGACGTGGGGG :  138
Aovo-DRB1*0306GA   : GGGTGCGGTACCTGGACAGATACTTCTATAACCAGGAGGAGTTCGTGCGCTTCGACAGCGACGTGGGGG :  138
Aovo-DRB1*0307GA   : GGGTGCGGTACCTGGACAGATACTTCTATAACCAGGAGGAGTTCGTGCGCTTCGACAGCGACGTGGGGG :  138
Aona-DRB3*0615     : GGGTGCGGTACCTGGACAGATACATCCATAACCAGGAGGAGGTCGTGCGCTTCGACAGCGACGTGGGGG :  138
Aona-DRB3*0627     : GGGTGCGGTACCTGGACAGATACATCCATAACCAGGAGGAGGTCGTGCGCTTCGACAGCGACGTGGGGG :  138
Aona-DRB3*062501   : GGGTGCGGTACCTGGACAGATACTTCTATAACCAGAAGGAGTATGTGCGCTTCGACAGCGACGTGGGGG :  138
Aona-DRB3*0626     : GGGTGCGGTACCTGGACAGATACTTCTATAACCAGAAGGAGTATGTGCGCTTCGACAGCGACGTGGGGG :  138
Aona-DRB3*062502   : GGGTGCGGTACCTGGACAGATACTTCTATAACCAGAAGGAGTATGTGCGCTTCGACAGCGACGTGGGGG :  138
Aona-DRB3*0628     : GGGTGCGGTACCTGGACAGATACCTTTATAACCAGAAGGAGTATGTGCGCTTCGACAGCGACGTGGGGG :  138
Aovo-DRB3*0601     : GGGTGCGGTACCTGGACAGATACTTCTATAACCAGAAGGAGTATGTGCGCTTCGACAGCGACGTGGGGG :  138
Aona-DRB*W8901     : GGGTGCGGTACCTGGACAGATACTTCTATAACCAGGAGGAGTATGTGCGCTTCGACAGCGACGTGGGGG :  138
Aona-DRB*W1808     : GGGTGCAGTTCCTGGAAAGATACTTTCATAACCAGGAGGAGTTGGTGCGCTTCGACAGCGACGTGGGGG :  138
Aona-DRB*W1806     : GGGTGCAGTTCCTGGAAAGATACTTTCATAACCAGGAGGAGTTCGTGCGCTTCGACAGCGACGTGGGGG :  138
Aovo-DRB*W1801     : GGGTGCAGTTCCTGGAAAGATACTTTTATAACCAGGAGGAGTATGTGCGCTTCGACAGCGACGTGGGGG :  138
Aovo-DRB*W1802     : GGGTGCAGTTCCTGGAAAGATACTTTTATAACCAGGAGGAGTATGTGCGCTTCAACAGCGACGTGGGGG :  138
Aovo-DRB*W1803     : GGGTGCAGTTCCTGGAAAGATACTTTTATAACCAGGAGGAGTATGTGCGCTTCGACAGCGACGTGGGGG :  138
Aovo-DRB*W8801     : GGGTGCGGCTCCTGCAAAGATACTTCTATAACCAGGAGGAGTATGTGCGCTTCGACAGCGACGTGGGGG :  138
Aovo-DRB*W2901     : GGGTGCGGCTCCTGCAAAGATACTTCTATAACCAGGAGGAGTATGTGCGCTTCGACAGCGACGTGGGGG :  138
Aona-DRB*W2908     : GGGTGCGGCTCCTGCAAAGATACTTCTATAACCAGGAGGAGTATGCGCGCTTCGACAGCGACGTGGGGG :  138
Aona-DRB*W2910     : GGGTGCGGCTCCTGCAAAGATACTTCTATAACCAGGAGGAGTATGCGCGCTTCGACAGCGACGTGGGGG :  138
Aovo-DRB*W3001     : GGGTGCGGTACCTGGAAAGACTCATCTATAACCGGGAGGAGTTCGTGCGCTTCGACAGCGACGTGGGGG :  138
Aona-DRB*W3002     : GGGTGCGGTACCTGGAAAGATACTTCTATAACCGGGAGGAGTTCGTGCGCTTCGACAGCGACGTGGGGG :  138
Aovo-DRB*W9201     : GGGTGCTGTACCTGGACAGATACTTCTATAACCAGGAGGAGTTCGTGCGCTTCGACAGCGACGTGGGGG :  138
Aovo-DRB*W9202     : GGGTGCGGTACCTGGACAGATACTTCTATAACCAGGAGGAGGTCGTGCGCTTCGACAGCGACGTGGGGG :  138
Aona-DRB*W9101     : GGGTGCGGTTCCTGGAAAGATACTTCTATAACCAGGAGGAGGTCCTGCGCTTCGACAGCGACGTGGGGG :  138
Aovo-DRB*W9101     : GGGTGCGGTTCCTGGAAAGATACTTCTATAACCAGGAGGAGGTCCTGCGCTTCGACAGCGACGTGGGGG :  138
Aovo-DRB*W9102     : GGGTGCGGTTCCTGGAAAGATACTTCTATAACCAGGAGGAGGTCCTGCGCTTCGACAGCGACGTGGGGG :  138
Aovo-DRB*W9001     : GGGTGCGGCTCCTGCAAAGATACTTCTATAACCAGGAGGAGGTCCTGCGCTTCGACAGCGACGTGGGGG :  138
Aovo-DRB*W4501     : GGGTGCGGTTCCTGGACAGATACATCCATAACCAGGAGGAGGTCGTGCGCTTCGACAGCGACGTGGGGG :  138
Aovo-DRB*W9301     : GGGTGCGGTTTCTGGAAAGACAAATCCATAACCAGGAGGAGTATCTGCGCTTCGACAGCGACGTGGGGG :  138
```

```
                     40         *         160         *         180         *         200
                     AGTaCCGGGCGGTGACGGAGCTGGGgCGGCctg  GC gAGtactggAACaGcCaGaAGGAc  c TGG
Aona-DRB1*0328GB   : AGTACCGGGCGGTGACGGAGCTGGGGCGGCGGAGCGCAGAGTACTGGAACAGCCAGAAGGACTTCCTGG :  207
Aona-DRB1*0329GA   : AGTACCGGGCGGTGACGGAGCTGGGGCGGCCTGACGCCGAGTACTGGAACAGCCAGAAGGACTACGTGG :  207
Aona-DRB1*031701GA : AGTACCGGGCGGTGACGGAGCTGGGGCGGCCTGACGCCGAGTACTGGAACAGCCAGAAGGACTACGTGG :  207
Aovo-DRB1*0304GA   : AGTACCGGGCGGTGACGGAGCTGGGGCGGCCTGACGCCGAGTACTGGAACAGCCAGAAGGACTACGTGG :  207
Aovo-DRB1*0305GA   : AGTACCGGGCGGTGACGGAGCTGGGGCGGCCTGACGCCGAGTACTGGAACAGCCAGAAGGACTACGTGG :  207
Aovo-DRB1*0306GA   : AGTACCGGGCGGTGACGGAGCTGGGGCGGCCTGACGCCGAGTACTGGAACAGCCAGAAGGACTACGTGG :  207
Aovo-DRB1*0307GA   : AGTACCGGGCGGTGACGGAGCTGGGGCGGCCTGACGCCGAGTACTGGAACAGCCAGAAGGACTACGTGG :  207
Aona-DRB3*0615     : AGTACCGGGCGGTGACGGAGCTGGGGCGGCCTGACGCCGAGTACTGGAACAGCCAGAAGGACTACGTGG :  207
Aona-DRB3*0627     : AGTACCGGGCGGTGACGGAGCTGGGGCGGCCTGACGCCGAGTACTGGAACAGCCAGAAGGACTACGTGG :  207
Aona-DRB3*062501   : AGTACCGGGCGGTGACGGAGCTGGGGCGGCCTGACGCCGAGTACTGGAACAGCCAGAAGGACTACGTGG :  207
Aona-DRB3*0626     : AGTACCGGGCGGTGACGGAGCTGGGGCGGCCTGACGCCGAGTACTGGAACAGCCAGAAGGACTACGTGG :  207
Aona-DRB3*062502   : AGTACCGGGCGGTGACGGAGCTGGGCCGGCCTGACGCCGAGTACTGGAACAGCCAGAAGGACTACGTGG :  207
Aona-DRB3*0628     : AGTACCGGGCGGTGACGGAGCTGGGGCGGCCTGACGCCGAGTACTGGAACAGCCAGAAGGACTACGTGG :  207
Aovo-DRB3*0601     : AGTACCGGGCGGTGACGGAGCTGGGGCGGCCTGACGCCGAGTACTGGAACAGCCAGAAGGACTACGTGG :  207
Aona-DRB*W8901     : AGTACCGGGCGGTGACGGAGCTGGGGCGGCGGACCGCAGAGTACTGGAACAGCCAGAAGGACTACGTGG :  207
Aona-DRB*W1808     : AGTACCGGGCGGTGACGGAGCTGGGGCGGCCTGATGCAAAGTACTGGAACGGTCAGCAGGACATCCTGG :  207
Aona-DRB*W1806     : AGTACCGGGCGGTGACGGAGCTGGGGCGGCCTGATGCAAAGTACTGGAACGGTCAGCAGGACATCCTGG :  207
Aovo-DRB*W1801     : AGTACCGGGCGGTGACGGAGCTGGGGCGGCCTGATGCAAAGTACTGGAACGGTCAGCAGGACATCCTGG :  207
Aovo-DRB*W1802     : AGTACCGGGCGGTGACGGAGCTGGGGCGGCCTGATGCAAAGTACTGGAACGGTCAGCAGGACATCCTGG :  207
Aovo-DRB*W1803     : AGTACCGGGCGGTGACGGAGCTGGGGCGGCCTGATGCAAAGTACTGGAACGGTCAGCAGGACATCCTGG :  207
Aovo-DRB*W8801     : AGTACCGGGCGGTGACGGAGCTGGGGCGGCCTGACGCCGAGTACTGGAACAGCCAGAAGGACATCCTGG :  207
Aovo-DRB*W2901     : AGTTCCGGGCGGTGACGGAGCTGGGGCGGCCTGACGCCGAGTACTGGAACAGCCTGAAGGAACGCCTGG :  207
Aona-DRB*W2908     : AGTTCCGGGCGGTGACGGAGCTGGGGCGGCCTGACGCCGAGTACTGGAACAGCCTGAAGGAACGCCTGG :  207
Aona-DRB*W2910     : AGTTCCGGGCGGTGACGGAGCTGGGGCGGCCTGACGCCGAGTACTGGAACAGCCTGAAGGAACGCCTGG :  207
Aovo-DRB*W3001     : AGTACCGGGCGGTGACGGAGCTGGGGCGGCCTGTTGCAGAGAAGCTCAACAGCCAGAAGGACATCCTGG :  207
Aona-DRB*W3002     : AGTACCGGGCGGTGACGGAGCTGGGGCGGCCTAGCGCAGAGAAGCTCAACAGCCAGAAGGACATCCTGG :  207
Aovo-DRB*W9201     : AGTACCGGGCGGTGACGGAGCTGGGGCGGCCTGTTGCAGAGAAGCTCAACAGCCAGAAGGACATCCTGG :  207
Aovo-DRB*W9202     : AGTACCGGGCGGTGACGGAGCTGGGGCGGCCTGTTGCAGAGAAGCTCAACAGCCAGAAGGACATCCTGG :  207
Aona-DRB*W9101     : AGTTCCGGGCGGTGACGGAGCTGGGGCGGCCTGAGGCCGAGTCCTGGAACAGCCAGAAGGACATCCTGG :  207
Aovo-DRB*W9101     : AGTTCCGGGCGGTGACGGAGCTGGGGCGGCCTGAAGCAGAGAAGTACAACAGCCAGAAGGACTTCCTGG :  207
Aovo-DRB*W9102     : AGTTCCGGGCGGTGACGGAGCTGGGGCGGCCTAGCGCAGAGAAGTACAACAGCCAGAAGGACATCCTGG :  207
Aovo-DRB*W9001     : AGTTCCGGGCGGTGACGGAGCTGGGGCGGCCTAGCGCAGAGAAGTTAAACAGCCAGAAGGAAAGCCTGG :  207
Aovo-DRB*W4501     : AGTACCGGGCGGTGACGGAGCTGGGGCGGCCTGACGCGGAGTACTGGAACAGCCAGAAGGACATCCTGG :  207
Aovo-DRB*W9301     : AGTACCGGGCGGTGACGGAGCTGGGGCGGCCTGACGCGGAGTCCTGGAACAGCCAGAAGGACTTAATGG :  207

                      *        220         *         240         *         260         *
                     AG   a GCGGG C  GGTgGACA CTaCTGcAgAcACAAcTACGGGGTTg TGAGAGCTTCACAGTGC
Aona-DRB1*0328GB   : AGGAGAGGCGGGCCTTGGTGGACACCTACTGTAGATACAACTACGGGGTTGCTGAGAGCTTCACAGTGC :  276
Aona-DRB1*0329GA   : AGCGGAAGCGGGGCCGGGTGGACAACTACTGCAGACACAACTACGGGGTTGGTGAGAGCTTCACAGTGC :  276
Aona-DRB1*031701GA : AGCAGAAGCGGGGCCGGGTGGACAACTACTGCAGACACAACTACGGGGTTGGTGAGAGCTTCACAGTGC :  276
Aovo-DRB1*0304GA   : AGCAGAAGCGGGGCCGGGTGGACAACTACTGCAGACACAACTACGGGGTTGGTGAGAGCTTCACAGTGC :  276
Aovo-DRB1*0305GA   : AGCAGAAGCGGGGCCGGGTGGACAACTACTGCAGACACAACTACGGGGTTGTTGAGAGCTTCACAGTGC :  276
Aovo-DRB1*0306GA   : AGCAGAAGCGGGGCCGGGTGGACAACTACTGCAGACACAACTACGGGGTTGGTGAGAGCTTCACAGTGC :  276
Aovo-DRB1*0307GA   : AGCAGAAGCGGGGCCGGGTGGACAACTACTGCAGACACAACTACGGGGTTGTTGAGAGCTTCACAGTGC :  276
Aona-DRB3*0615     : AGCAGAAGCGGGGCCGGGTGGACAACTACTGCAGACACAACTACGGGGTTGTTGAGAGCTTCACAGTGC :  276
Aona-DRB3*0627     : AGCAGAAGCGGGGCCAGGTGGACAACTACTGCAGACACAACTACGGGGTTTTTGAGAGCTTCACAGTGC :  276
Aona-DRB3*062501   : AGCAGAAGCGGGGCCGGGTGGACAACTACTGCAGACACAACTACGGGGTTGTTGAGAGCTTCACAGTGC :  276
Aona-DRB3*0626     : AGCAGAAGCGGGGCCGGGTGGACAACTACTGCAGACACAACTACGGGGTTGTTGAGAGCTTCACAGTGC :  276
Aona-DRB3*062502   : AGCAGAAGCGGGGCCGGGTGGACAACTACTGCAGACACAACTACGGGGTTGTTGAGAGCTTCACAGTGC :  276
Aona-DRB3*0628     : AGCAGAAGCGGGGCCGGGTGGACAACTACTGCAGACACAACTACGGGGTTGTTGAGAGCTTCACAGTGC :  276
Aovo-DRB3*0601     : AGCAGAAGCGGGGCCGGGTGGACAACTACTGCAGACACAACTACGGGGTTGTTGAGAGCTTCACAGTGC :  276
Aona-DRB*W8901     : AGCAGAAGCGGGGCCGGGTGGACAACTACTGCAGACACAATTACGGGGTTGCTGAGAGCTTCACAGTGC :  276
Aona-DRB*W1808     : AGCTCAAGCGGGGCCAGGTAGACAACTACTGCAGACACAACTACGGGGTTTTTGAGAGCTTCACAGTGC :  276
Aona-DRB*W1806     : AGCTGAAGCGGGGCCAGGTAGACAACTACTGCAGACACAACTACGGGGTTGGTGAGAGCTTCACAGTGC :  276
Aovo-DRB*W1801     : AGCTGAAGCGGGGCCAGGTAGACAACTACTGCAGACACAACTACGGGGTTTTTGAGAGCTTCACAGTGC :  276
Aovo-DRB*W1802     : AGCTGAAGCGGGGCCAGGTAGACAACTACTGCAGACACAACTACGGGGTTTTTGAGAGCTTCACAGTGC :  276
Aovo-DRB*W1803     : AGCTGAAGCGGGGCCAGGTAGACAACTACTGCAGACACAACTACGGGGTTTTTGAGAGCTTCACAGTGC :  276
Aovo-DRB*W8801     : AGTATCTGCGGGCCGCGGTGGACAACTACTGCAGACACAACTACGGGGTTGCTGAGAGCTTCACAGTGC :  276
Aovo-DRB*W2901     : AGTATCTGCGGGCCGCGGTGGACACCTGCTGCAGACACAACTACGGGGTTGTTGAGAGCTTCACAGTGC :  276
Aona-DRB*W2908     : AGTATCTGCGGGCCGCGGTGGACACCTACTGCAGACACAACTACGGGGTTGGTGAGAGCTTCACAGTGC :  276
Aona-DRB*W2910     : AGTATCTGCGGGCCGCGGTGGACACCTACTGCAGACACAACTACGGGGTTTTTGAGAGCTTCACAGTGC :  276
Aovo-DRB*W3001     : AGGACAGGCGGGCCTCGGTGGACACCTACTGTAAACACAACTACGGGGTTGGTGAGAGCTTCACAGTGC :  276
Aona-DRB*W3002     : AGGACAGGCGGGCCGCGGTGGACACCTACTGTAAACACAACTACGGGGTTGGTGAGAGCTTCACAGTGC :  276
Aovo-DRB*W9201     : AGGACAGGCGGGCCTCGGTGGACACCTACTGTAAACACAACTACGGGGTTGGTGAGAGCTTCACAGTGC :  276
Aovo-DRB*W9202     : AGGACAGGCGGGCCTCGGTGGACACCTACTGTAAACACAACTACGGGGTTGGTGAGAGCTTCACAGTGC :  276
Aona-DRB*W9101     : AGACCAGGCGGGCCGCGGTGGACACCTTCTGCAGACACAACTACGGGGTTGGTGAGAGCTTCACAGTGC :  276
Aovo-DRB*W9101     : AGACCAGGCGGGCCGCGGTGGACACCTTCTGCAGACACAACTACGGGGTTGGTGAGAGCTTCACAGTGC :  276
Aovo-DRB*W9102     : AGACCAGGCGGGCCGCGGTGGACACCTTCTGCAGACACAACTACGGGGTTTTTGAGAGCTTCACAGTGC :  276
Aovo-DRB*W9001     : AGTATCTGCGGGCCGCGGTGGACACCTACTGCAGACACAACTACGGGGTTGGTGAGAGCTTCACAGTGC :  276
Aovo-DRB*W4501     : AGGACAAGCGGGCCTCGGTGGACACCTACTGCAGACACAACTACGGGGTTGTTGAGAGCTTCACAGTGC :  276
Aovo-DRB*W9301     : AGGACAGGCGGGCCGCGGTGGACACCTACTGCAGACACAACTACGGGGTTGTTGAGAGCTTCACAGTGC :  276
```

```
                              280         *        300         *        320         *        340
                            AGCGGAgAGGTGAGcGCGGCGGGGcGGGGCCTCCCTGTGAgCTGCcgaTCAGAGA      gaga  ga
Aona-DRB1*0328GB    : AGCGGAGAGGTGAGCGCGGCGGGGCGGGGCCTCCCTGTGAGCTGCCGATCAGAGAGAGACTGAGAGAGA :  345
Aona-DRB1*0329GA    : AGCGGAGAGGTGAGCGCGGCGGGGCGGGGCCTCCCTGTGAGCTGCCGATCAGAGAGAGACTGAAAGAGA :  345
Aona-DRB1*031701GA  : AGCGGAGAGGTGAGCGCGGCGGGGCGGGGCCTCCCTGTGAGCTGCCGATCAGAGAGAGACTGAGAGAGA :  345
Aovo-DRB1*0304GA    : AGCGGAGAGGTGAGCGCGGCGGGGCGGGGCCTCCCTGTGAGCTGCCGATCAGAGAGAGACTGAGAGAGA :  345
Aovo-DRB1*0305GA    : AGCGGAGAGGTGAGCGCGGCGGGGCGGGGCCTCCCTGTGAGCTGCCGATCAGAGAGAGACTGAGAGAGA :  345
Aovo-DRB1*0306GA    : AGCGGAGAGGTGAGCGCGGCGGGGCGGGGCCTCCCTGTGAGCTGCCGATCAGAGAGAGACTGAGAGAGA :  345
Aovo-DRB1*0307GA    : AGCGGAGAGGTGAGCGCGGCGGGGCGGGGCCTCCCTGTGAGCTGCCGATCAGAGAGAGACTGAGAGAGA :  345
Aona-DRB3*0615      : AGCGGAGAGGTGAGCGCGGCGGGGCGGGGCCTCCCTGTGAGCTGCCGATCAGAGAGAGACTGAGAGAGA :  345
Aona-DRB3*0627      : AGCGGAGAGGTGAGCGCGGCGGGGCGGGGCCTCCCTGTGAGCTGCCGATCAGAGAGAGACTGAGAGAGA :  345
Aona-DRB3*062501    : AGCGGAGAGGTGAGCGCGGCGGGGCGGGGCCTCCCTGTGAGCTGCCGATCAGAGAGAGACTGAGAGAGA :  345
Aona-DRB3*0626      : AGCGGAGAGGTGAGCGCGGCGGGGCGGGGCCTCCCTGTGAGCTGCCGATCAGAGAGAGACTGAGAGAGA :  345
Aona-DRB3*062502    : AGCGGAGAGGTGAGCGCGGCGGGGCGGGGCCTCCCTGTGAGCTGCCGATCAGAGAGAGACTGAGAGAGA :  345
Aona-DRB3*0628      : AGCGGAGAGGTGAGCGCGGCGGGGCGGGGCCTCCCTGTGAGCTGCCGATCAGAGAGAGACTGAGAGAGA :  345
Aovo-DRB3*0601      : AGCGGAGAGGTGAGCGCGGCGGGGCGGGGCCTCCCTGTGAGCTGCCGATCAGAGAGAGACTGAGAGAGA :  345
Aona-DRB*W8901      : AGCGGAGAGGTGAGCGCGGCGGGGCGGGGCCTCCCTGTGAGCTGCCGATCAGAGA----CAGAGACTGA :  341
Aona-DRB*W1808      : AGCGGAGAGGTGAGTGCGGCGGGGCGGGGCCTCCCTGTGAGCTGCCGATCAGAGA----GAGAGACTGA :  341
Aona-DRB*W1806      : AGCGGAGAGGTGAGTGCGGCGGGGCGGGGCCTCCCTGTGAGCTGCCGATCAGAGA----GAGAGACTGA :  341
Aovo-DRB*W1801      : AGCGGAAAGGTGAGTGCGGCGGGGCGGGGCCTCCCTGTGAGCTGCCGATCAGAGA----GAGAGA---- :  337
Aovo-DRB*W1802      : AGCGGAAAGGTGAGTGCGGCGGGGCGGGGCCTCCCTGTGAGCTGCCGATCAGAGA----GAGAGACTGA :  341
Aovo-DRB*W1803      : AGCGGAAAGGTGAGTGCGGCGGGGCGGGGCCTCCCTGTGAGCTGCCGATCAGAGA----GAGAGACTGA :  341
Aovo-DRB*W8801      : AGCGGAGAGGTGAGCGCGGCGGGGCGGGGCCTCCCTGTGAGCTGCCGATCAGAGAGAGACAGAGAGAGA :  345
Aovo-DRB*W2901      : AGCGGAGAGGTGAGCGCGGCGGGGCGGGGCCTCCCTGTGAGCTGCCGATCAGAGA----CAGAGACTGA :  341
Aona-DRB*W2908      : AGCGGAGAGGTGAGCGCGGCGGGGCGGGGCCTCCCTGTGAGCTGCCGATCAGAGA----CAGAGACTGA :  341
Aona-DRB*W2910      : AGCGGAGAGGTGAGCGCGGCGGGGCGGGGCCTCCCTGTGAGCTGCCGATCAGAGA----CAGAGACTGA :  341
Aovo-DRB*W3001      : AGCGGAGAGGTGAGCGCGGCGGGGCGGGGCCTCCCTGTGAGCTGCGAATCAGAGA----CAGAGACAGA :  341
Aona-DRB*W3002      : AGCGGAGAGGTGAGCGCGGCGGGGCGGGGCCTCCCTGTGAGCTGCGGATCAGAGA----CAGAGACAGA :  341
Aovo-DRB*W9201      : AGCGGAGAGGTGAGCGCGGCGGGGCGGGGCCTCCCTGTGAGCTGCGAATCAGAGA----CAGAGACAGA :  341
Aovo-DRB*W9202      : AGCGGAGAGGTGAGCGCGGCGGGGCGGGGCCTCCCTGTGAGCTGCGAGTCAGAGA----CAGAGACAGA :  341
Aona-DRB*W9101      : AGCGGAGAGGTGAGCGCGGCGGGACGGGGCCTCCCTGTGAACTGCCAATCAGAGA-------------- :  331
Aovo-DRB*W9101      : AGCGGAGAGGTGAGCGCGGCGGGACGGGGCCTCCCTGTGAACTGCCAATCAGAGA-------------- :  331
Aovo-DRB*W9102      : AGCGGAGAGGTGAGCGCGGCGGGGCGGGGCCTCCCTGTGAGCTGCCGATCAGAGA-------------- :  331
Aovo-DRB*W9001      : AGCGGAGAGGTGAGCGCGGCGGGGCGGGGCCTCCCTGTGAGCTGCCGATCAGAGA-------------- :  331
Aovo-DRB*W4501      : AGCGGAGAGGTGAGCGCGGCGGGGCGGGGCCTCCCTGTGAGCTGCGAATCAGAGA----CAGAGACTGA :  341
Aovo-DRB*W9301      : AGCGGAGAGGTGAGCGCGGCGGGGCGGGGCCTCCCTGTGAGCTGCCGATCAGAGA-------------- :  331

                                *       360         *        380         *        400         *
                                                                                  gAga agagaGA  gA
Aona-DRB1*0328GB    : CTGAGAGAGAC------CGAGAGAGAGACACAGAGACACTGAGAGA--GACAGAGAGAGAGAGACTGAG :  406
Aona-DRB1*0329GA    : CTGAGAGAGACACTGAGTGAGAGTGAGACAGAGAGACAGAGAAAGACTGACAGACAGAGAGAGACTGAG :  414
Aona-DRB1*031701GA  : CTGAGAGAGACACTGAGAGAGAGTGAGACAGAGAGACAGAGAAAGACTGACAGACAGAGAGAGACTGAG :  414
Aovo-DRB1*0304GA    : CCGAGAGAGA---------------GACACAGAGACACTGAGAGA--GACAGAGAGAGAGAGACTGAG :  396
Aovo-DRB1*0305GA    : CCGAGAGAGA---------------GACACAGAGACACTGAGAGA--GACAGAGAGAGAGAGACTGAG :  396
Aovo-DRB1*0306GA    : CCGAGAGAGA---------------GACACAGAGACACTGAGAGA--GACAGAGAGAGAGAGACTGAG :  396
Aovo-DRB1*0307GA    : CCGAGAGAGA-----------GAGAGACACAGAGACACTGAGAGA--GACAGAGAGAGAGAGACTGAG :  400
Aona-DRB3*0615      : CTGAGAGAGAC------CGAGAGAGAGACACAGAGACACTGAGAGA--GACAGAGAGAGAGAGACTGAG :  406
Aona-DRB3*0627      : CTGAGAGAGAC------CGAGAGAGAGACACAGAGACACTGAGAGA--GACAGAGAGAGAGAGACTGAG :  406
Aona-DRB3*062501    : CTGAGAGAGAC------CGAGAGAGAGACACAGAGACACTGAGAGA--GACAGAGAGAGAGAGACTGAG :  406
Aona-DRB3*0626      : CTGAGAGAGAC------CGAGAGAGAGACACAGAGACACTGAGAGA--GACAGAG--AGAGAGACTGAG :  404
Aona-DRB3*062502    : CCGAGAGAGAC------CGAGAGAGAGACACAGAGACACTGAGAGA--GACAGAGAGAGAGAGACTGAG :  406
Aona-DRB3*0628      : CTGAGAGAGAC------CGAGAGAGAGACACAGAGACACTGAGAGA--GACAGAGAGAGAGAGACTGAG :  406
Aovo-DRB3*0601      : CTGAGAGAGAC------CGAGAGAGAGACACAGAGACACTGAGAGA--GACAGAG-GAGAGAGACTGAG :  405
Aona-DRB*W8901      : GA-----------------------------------------------GAGACTGAGAGAGAGAG :  360
Aona-DRB*W1808      : GAGAGACTGAG---------------------AGAGAGAGAGAGA------GAGACACAGAGAGAGAT :  382
Aona-DRB*W1806      : GAGAGACTGAG---------------------AGAGAGAGAGAGA------GAGACACAGAGAGAGAT :  382
Aovo-DRB*W1801      : -------------------------------------------------GAGACACAGAGAGAGAT :  354
Aovo-DRB*W1802      : GAGAGACTGAG---------------------AGAGAG----AGA------GAGAGAGAGAGAGACAC :  378
Aovo-DRB*W1803      : GAGAGAGAGAG---------------------AGAGAGAGAGAGA------GAGACACAGAGAGAGAT :  382
Aovo-DRB*W8801      : GA-----------------------------------------------GAGACTGAGAGAGAGAG :  364
Aovo-DRB*W2901      : GA-----------------------------------------------GAGACTGAGAGAGAGAG :  360
Aona-DRB*W2908      : GA-----------------------------------------------GAGACTGAGAGAGAGAG :  360
Aona-DRB*W2910      : GA-----------------------------------------------GAGACTGAGAGAGAGAG :  360
Aovo-DRB*W3001      : GA-----------------------------------------------GAGAGAGAGAGAGAGAA :  360
Aona-DRB*W3002      : GA-----------------------------------------------GAGAGAGAGAGAGAAAG :  360
Aovo-DRB*W9201      : GA-----------------------------------------------GAGAGAGAGAGAGAGAA :  360
Aovo-DRB*W9202      : GA-----------------------------------------------GAGAGAGAGAGAGAAAG :  360
Aona-DRB*W9101      : -------------------------------------------------CAGAGAGACCGAGAGAG :  348
Aovo-DRB*W9101      : -------------------------------------------------CAGAGAGACCGAGAGAG :  348
Aovo-DRB*W9102      : -------------------------------------------------CAGAGAGACCGAGAGAG :  348
Aovo-DRB*W9001      : -------------------------------------------------CAGAGACTGAGAGAGAC :  348
Aovo-DRB*W4501      : GA-----------------------------------------------GAGACTGAGAGACAGAC :  360
Aovo-DRB*W9301      : -------------------------------------------------GAGACTGAGAGAGAGAG :  348
```

```
                         420         *        440         *        460         *        480
                     aga
Aona-DRB1*0328GB   : AGA-GACAGTGA--GAGA------------------------------CTGAGAGACTGAGACTGAGAG :  442
Aona-DRB1*0329GA   : AGG-TACACTGA--GAGAGACAGAGTGAGACAGAGAGACAGAGTAAGACTGACAGAGAGAGACTGAGAG :  480
Aona-DRB1*031701GA : AGG-TACACTGA--GAGAGACAGAGTGAGACAGAGAGACAGAGTAAGACTGACAGAGAGAGACTGAGAG :  480
Aovo-DRB1*0304GA   : AGA-GACAGTGA--GAGACT--------------------------GAGAGACTGAGAGAGAC------ :  430
Aovo-DRB1*0305GA   : AGA-GACAGTGA--GAGA------------------------------CTGAGAGACTGAGACTGAGAG :  432
Aovo-DRB1*0306GA   : AGA-GACAGTGA--GAGACT--------------------------GAGAGACTGAGAGAGACTGAGAG :  436
Aovo-DRB1*0307GA   : AGA-GACAGTGA--GAGACT--------------------------GAGAGACTGAGAGAGACTGAGAG :  440
Aona-DRB3*0615     : AGA-GACAGTGA--GAGACT--------------------------GAGAGACTGAGAGAGACTGAGAG :  446
Aona-DRB3*0627     : AGA-GACAGTGA--GAGACT--------------------------GAGAGACTGAGAGAGACTGAGAG :  446
Aona-DRB3*062501   : AGA-GACAGTGA--GAGACT--------------------------GAGAGACTGAGAGAGACTGAGAG :  446
Aona-DRB3*0626     : AGA-GACAGTGA--GAGACT--------------------------GAGAGACTGAGAGAGACTGAGAG :  444
Aona-DRB3*062502   : AGA-GACAGTGA--GAGACT--------------------------GAGAGACTGAGAGAGACTGAGAG :  446
Aona-DRB3*0628     : AGA-GACAGTGA--GAGACT--------------------------GAGAGACTGAGAGAGACTGAGAG :  446
Aovo-DRB3*0601     : AGA-GACAGTGA--GAGACT--------------------------GAGAGACTGAGAGAGACTGAGAG :  445
Aona-DRB*W8901     : AGA------------------------------------------------------------------ :  363
Aona-DRB*W1808     : AGAC--AGAGACAGA--GAG------------------------------------------------- :  398
Aona-DRB*W1806     : AGAC--AGAGACAGA--GAG------------------------------------------------- :  398
Aovo-DRB*W1801     : AGAC--AGAGACAGA--GAG------------------------------------------------- :  370
Aovo-DRB*W1802     : AGAG--AGAGATAGACAGAG------------------------------------------------- :  396
Aovo-DRB*W1803     : AGAC--AGAGACAGA--GAG------------------------------------------------- :  398
Aovo-DRB*W8801     : AGAC--TGAGAGAGAGAGAG------------------------------------------------- :  382
Aovo-DRB*W2901     : AGA------------------------------------------------------------------ :  363
Aona-DRB*W2908     : AGA------------------------------------------------------------------ :  363
Aona-DRB*W2910     : AGA------------------------------------------------------------------ :  363
Aovo-DRB*W3001     : AGA------------------------------------------------------------------ :  363
Aona-DRB*W3002     : AGA------------------------------------------------------------------ :  363
Aovo-DRB*W9201     : AGA------------------------------------------------------------------ :  363
Aovo-DRB*W9202     : AGA------------------------------------------------------------------ :  363
Aona-DRB*W9101     : AGA------------------------------------------------------------------ :  351
Aovo-DRB*W9101     : A-------------------------------------------------------------------- :  349
Aovo-DRB*W9102     : CGA------------------------------------------------------------------ :  351
Aovo-DRB*W9001     : TGA------------------------------------------------------------------ :  351
Aovo-DRB*W4501     : TGA------------------------------------------------------------------ :  363
Aovo-DRB*W9301     : ACT------------------------------------------------------------------ :  351

                           *        500         *        520         *        540         *
                                  gagaga a
Aona-DRB1*0328GB   : AGAC------ACTGAGAGAGACAGAGT----GAGACAGAGAGACA--GAGAAAGGCTGACAGACAGAGA :  499
Aona-DRB1*0329GA   : ATAC------ACTGAGAGAGACAGAGACTGAGAGACAGAGAGACACTGAGAGAGACAGAGAGACTGAAA :  543
Aona-DRB1*031701GA : ATAC------ACTGAGAGAGACAGAGACTGAGAGACAGCGAGACACTGAGAGAGACAGAGAGACTGAAA :  543
Aovo-DRB1*0304GA   : ----------ACTGAGAGAGACAGAGT----GAGACAGAGAAACA--GAGAAAGAC------------- :  470
Aovo-DRB1*0305GA   : AGAC------ACTGAGAGAGACAGAGG----GAGACAGAGAGACA--GAGAAAGACTGAGAGACAGAGA :  489
Aovo-DRB1*0306GA   : AGAC------ACTGAGAGAGACAGAGT----GAGACAGAGAAACA--GAGAAAGAC------------- :  480
Aovo-DRB1*0307GA   : AGAC------ACTGAGAGAGACAGAGT----GAGACAGAGAAACA--GAGAAAGAC------------- :  484
Aona-DRB3*0615     : AGAC------ACTGAGAGAGACAGAGT----GAGACAGAGAAACA--GAGAAAGAC------------- :  490
Aona-DRB3*0627     : AGAC------ACTGAGAGAGACAGAGG----GAGACAGAGAGACA--GAGAAAGACTGAGAGACAGAGA :  503
Aona-DRB3*062501   : AGAC------ACTGAGAGAGACAGAGT----GAGACAGAGAAACA--GAGAAAGAC------------- :  490
Aona-DRB3*0626     : AGAC------ACTGAGAGAGACAGAGT----GAGACAGAGAAACA--GAGAAAGAC------------- :  488
Aona-DRB3*062502   : AGAC------ACTGAGAGAGACAGAGT----GAGACAGAGAAACA--GAGAAAGAC------------- :  490
Aona-DRB3*0628     : AGAC------ACTGAGAGAGACAGAGG----GAGACAGAGAGACA--GAGAAAGACTGAGAGACAG--- :  500
Aovo-DRB3*0601     : AGAC------ACTGAGAGAGACAGAGT----GAGACAGAGAAACA--GAGAAAGAC------------- :  489
Aona-DRB*W8901     : -------------ATGAGAGAGACTGA------------------------------------------ :  377
Aona-DRB*W1808     : --ACTGAGAGACTGTGAGAGAGACTGA------------------------------------------ :  423
Aona-DRB*W1806     : --ACTGAGAGACTGTGAGAGAGACTG------------------------------------------- :  422
Aovo-DRB*W1801     : --ACTGAGAGACTGTGAGAGAGACTGA------------------------------------------ :  395
Aovo-DRB*W1802     : --ACTGAGAGACTGTGAGAGAGACTG------------------------------------------- :  420
Aovo-DRB*W1803     : --ACTGAGAGACTGTGAGAGAGACTG------------------------------------------- :  422
Aovo-DRB*W8801     : --ACTGAGAGAGAATGAGAGAGACTGA------------------------------------------ :  407
Aovo-DRB*W2901     : -------------ATGAAAGAGACTGA------------------------------------------ :  377
Aona-DRB*W2908     : -------------ATGAGAGAGACTGA------------------------------------------ :  377
Aona-DRB*W2910     : -------------ATGAGAGAGACTGA------------------------------------------ :  377
Aovo-DRB*W3001     : -------------GAGAGAGAGAG--------------------------------------------- :  374
Aona-DRB*W3002     : -------------GAGAGAGAGAG--------------------------------------------- :  374
Aovo-DRB*W9201     : -------------GAGAGAGAGAG--------------------------------------------- :  374
Aovo-DRB*W9202     : -------------GAGAGAGAGAA--------------------------------------------- :  374
Aona-DRB*W9101     : -------------CTGAGAGAGACTGC------------------------------------------ :  365
Aovo-DRB*W9101     : --------------------------------------------------------------------- :    -
Aovo-DRB*W9102     : -------------CTGAGAGAGACTGA------------------------------------------ :  365
Aovo-DRB*W9001     : -------------GAGA---------------------------------------------------- :  355
Aovo-DRB*W4501     : -------------GAGAGAGAGTG--------------------------------------------- :  374
Aovo-DRB*W9301     : -------------GAGAG--------------------------------------------------- :  356
```

```
                           560       *       580       *       600       *       620

Aona-DRB1*0328GB   : GAGACTGAGAGATACACTGAGAG----AGACAGAGACTGAGAGAC----AGACACT----------GAG :  550
Aona-DRB1*0329GA   : GA--CTGAGAGAGACAGAGAGAGACTGAGAGAGAGACTGAGAGACTGAGAGATACTTAGGGAGACTGAG :  610
Aona-DRB1*031701GA : GA--CTGAGAGAGACAGAGAGAGACTGAGAGAGAGACTGAGAGACTGAGAGATACTTAGGGAGACTGAG :  610
Aovo-DRB1*0304GA   : -----TGAGAGATACACTGAGAG----AGACAGAGACTGAGAGACAGAGAGACACT----------GAG :  520
Aovo-DRB1*0305GA   : GA--CTGAGAGATACACTGAGAG----AGACAGAGACTGAGAGACAGGGAGACACT----------GAG :  542
Aovo-DRB1*0306GA   : -----TGAGAAATACACTGAGAG----AGACAGAGACTGAGAGACAGAGAGACACT----------GAG :  530
Aovo-DRB1*0307GA   : -----TGAGAGATACACTGAGAG----AGACAGAGACTGAGAGACAGAGAGACACT----------GAG :  534
Aona-DRB3*0615     : -----TGAGAGATACACTGAGAG----AGACAGAGACTGAGAGACAGAGAGACAC------------AG :  538
Aona-DRB3*0627     : GAGACTGAGAGATACACTGAGAG----AGACAGAGACTGAGAGACAGAGAGACACT----------GAG :  558
Aona-DRB3*062501   : -----TGAGAGATACACTGAGAG----AGACAGAGACTGAGAGACAGAGAGACAC------------AG :  538
Aona-DRB3*0626     : -----TGAGAGATACACTGAGAG----AGACAGAGACTGAGAGACAGAGAGACAC------------AG :  536
Aona-DRB3*062502   : -----TGAGAGATACACTGAGAG----AGACAGAGACTGAGAGACAGAGAGACAC------------AG :  538
Aona-DRB3*0628     : -AGACTGAGAGATACACTGAGAG----AGACAGAGACTGAGAGACAGAGAGACACT----------GAG :  554
Aovo-DRB3*0601     : -----TGAGAGATACACTGAGAG----AGACAGAGACTGAGAGACAGAGAGACAC------------AG :  537
Aona-DRB*W8901     : ---------------GA----------------GAGAGACTGAGAGAGACTGAGA-------------- :  401
Aona-DRB*W1808     : ---------------GAGAGACT------GTGCAAGACCGAGAGAGAGACTGAGA-------------- :  457
Aona-DRB*W1806     : ------------------------------TGCAAGACCGAGAGAGAGACTGAGA-------------- :  447
Aovo-DRB*W1801     : ---------------GAGAGACT------GTGCAAGACCGAGAGAGAGATTGAGA-------------- :  429
Aovo-DRB*W1802     : ------------------------------TGCAAGACCGAGAGAGAGACTGAGA-------------- :  445
Aovo-DRB*W1803     : ------------------------------------------AGAGAGACTGTG--------------- :  434
Aovo-DRB*W8801     : ---------------GA----------------GAGAGACTGAGAGAGACTGAGA-------------- :  431
Aovo-DRB*W2901     : ---------------GA----------------GAGAGACTGAGAGAGACTGAGA-------------- :  401
Aona-DRB*W2908     : ---------------GA----------------GAGAGACTGAGAGAGACTGAGA-------------- :  401
Aona-DRB*W2910     : ---------------GA----------------GAGAGACTGAGAGAGACTGAGA-------------- :  401
Aovo-DRB*W3001     : ---------------------------------AAGACAGAGAGAGAGAGAGAGA-------------- :  396
Aona-DRB*W3002     : ----------------A----------------GAGAGAAAGAGAGAGAGAGAGAA------------- :  398
Aovo-DRB*W9201     : ---------------------------------AAGACAGAGAGAGAGAGAGAGA-------------- :  396
Aovo-DRB*W9202     : ---------------------------------GACAGAGAGAGAGAGAGAGAGA-------------- :  396
Aona-DRB*W9101     : ---------------------------------------GAGAGAGA---------------------- :  373
Aovo-DRB*W9101     : --------------------------------------------------------------------- :    -
Aovo-DRB*W9102     : ---------------------------------------GAGAGAGA---------------------- :  373
Aovo-DRB*W9001     : --------------------------------------------------------------------- :    -
Aovo-DRB*W4501     : --------------------------------------AGAGAGAGAGAGAGAGA-------------- :  391
Aovo-DRB*W9301     : --------------------------------------------------------------------- :    -

                             *       640       *       660       *       680       *
                                                      agaga   a
Aona-DRB1*0328GB   : AGAGACTGAGAGACTG--------TGAGAGAGAGACTGAGAGAGACAGA----GAGAGACTGAGAGAGA :  607
Aona-DRB1*0329GA   : AGAGACAGAGAGACTGAGACAGACTGAGAGAAAGACTGAGAGAGAGAGACTGAGAGAGGCTGAGAGAGA :  679
Aona-DRB1*031701GA : AGAGACAGAGAGACTGAGACAGACTGAGAGAAAGACTGAGAGAGAGAGACTGAGAGAGGCTGAGAGAGA :  679
Aovo-DRB1*0304GA   : AGAGACAGAGAGACTGA--------------AAGACTGAGAGAGACAGA------GAGACTGAGAGAAA :  569
Aovo-DRB1*0305GA   : AGAGACAGAGAGACTGA--------------AAGACTGAGAGAGACAGA----GAGAGACTGAGAGAGA :  593
Aovo-DRB1*0306GA   : AGAGACAGAGAGACTGA--------------AAGACTGAGAGAGACAGA------GAGACTGAGAGAAA :  579
Aovo-DRB1*0307GA   : AGAGACAGAGAGACTGA--------------AAGACTGAGAGAGACAGA------GAGACTGAGAGAAA :  583
Aona-DRB3*0615     : AGAGACAGAGAGACTGA--------------AAGACTGAGAGAGACAGA------GAGACTGAGAGAGA :  587
Aona-DRB3*0627     : AGAGACAGAGAGACTGA--------------AAGACTGAGAGAGACAGA----GAGAGACTGAGAGAGA :  609
Aona-DRB3*062501   : AGAGACAGAGAGACTGA--------------AAGACTGAGAGAGACAGA------GAGACTGAGAGAGA :  587
Aona-DRB3*0626     : AGAGACAGAGAGACTGA--------------AAGACTGAGAGAGACAGA------GAGACTGAGAGAGA :  585
Aona-DRB3*062502   : AGAGACAGAGAGACTGA--------------AAGACTGAGAGAGACAGA------GAGACTGAGAGAGA :  587
Aona-DRB3*0628     : AGAGACAGAGAGACTGA--------------AAGACTGAGAGAGACAGA----GAGAGACTGAGAGAGA :  605
Aovo-DRB3*0601     : AGAGACAGAGAGACTGA--------------AAGACTGAGAGAGACAGA------GAGACTGAGAGAGA :  586
Aona-DRB*W8901     : -----------------------------------GATAGAGACTGA---------------------- :  413
Aona-DRB*W1808     : -----------------------------------CTGAGAGATTGAGA------------GAGACAGA :  479
Aona-DRB*W1806     : -----------------------------------CTGAGAGATTGAGA------------GAGACAGA :  469
Aovo-DRB*W1801     : -----------------------------------CTGAGAGATTGAGA------------GAGACAGA :  451
Aovo-DRB*W1802     : -----------------------------------CTGAGAGATTGAGA------------GAGACAGA :  467
Aovo-DRB*W1803     : ------------------------------------CAAGACCGAGAGA------------GAGACTGA :  455
Aovo-DRB*W8801     : -----------------------------------GAGAGAGACTGAGA------------GAGAGAGA :  453
Aovo-DRB*W2901     : -----------------------------------GATAGAGACTGA---------------------- :  413
Aona-DRB*W2908     : -----------------------------------GATAGAGACTGA---------------------- :  413
Aona-DRB*W2910     : -----------------------------------GATAGAGACTGA---------------------- :  413
Aovo-DRB*W3001     : -----------------------------------GAGAGAGA--GA---------------------- :  406
Aona-DRB*W3002     : -----------------------------------GACAGAGAGAGA---------------------- :  410
Aovo-DRB*W9201     : -----------------------------------GAGAGAGA--GA---------------------- :  406
Aovo-DRB*W9202     : -----------------------------------GAGAGAGA--GA---------------------- :  406
Aona-DRB*W9101     : --------------------------------------------------------------------- :    -
Aovo-DRB*W9101     : --------------------------------------------------------------------- :    -
Aovo-DRB*W9102     : --------------------------------------------------------------------- :    -
Aovo-DRB*W9001     : --------------------------------------------------------------------- :    -
Aovo-DRB*W4501     : -----------------------------------GAGAGAGG-------------------------- :  399
Aovo-DRB*W9301     : --------------------------------------------------------------------- :    -
```

```
                         700         *        720         *        740         *        76

Aona-DRB1*0328GB   : CACTGAGAGAGACAGAGA--GACTGAAAGACTGAGAGATACTTAGGGAGACTGAGAGAAAC-------A :  667
Aona-DRB1*0329GA   : GACTGAGGGAGACAGAGAATGAGAGAGAGACTGAGAGACAAG-ACAGAGACTGAGAGAGATGAGACAGA :  747
Aona-DRB1*031701GA : GACTGAGGGAGACCGAGAATGAGAGAGAGACTGAGAGACAAG-ACAGAGACTGAGAGAGATGAGACAGA :  747
Aovo-DRB1*0304GA   : --------------------------------------------------------------------- :    -
Aovo-DRB1*0305GA   : GACT----------------------------------------------------------------- :  597
Aovo-DRB1*0306GA   : --------------------------------------------------------------------- :    -
Aovo-DRB1*0307GA   : --------------------------------------------------------------------- :    -
Aona-DRB3*0615     : --------------------------------------------------------------------- :    -
Aona-DRB3*0627     : GGCT----------------------------------------------------------------- :  613
Aona-DRB3*062501   : --------------------------------------------------------------------- :    -
Aona-DRB3*0626     : --------------------------------------------------------------------- :    -
Aona-DRB3*062502   : --------------------------------------------------------------------- :    -
Aona-DRB3*0628     : GACT----------------------------------------------------------------- :  609
Aovo-DRB3*0601     : --------------------------------------------------------------------- :    -
Aona-DRB*W8901     : --------------------------------------------------------------------- :    -
Aona-DRB*W1808     : --------------------------------------------------------------------- :    -
Aona-DRB*W1806     : --------------------------------------------------------------------- :    -
Aovo-DRB*W1801     : --------------------------------------------------------------------- :    -
Aovo-DRB*W1802     : --------------------------------------------------------------------- :    -
Aovo-DRB*W1803     : --------------------------------------------------------------------- :    -
Aovo-DRB*W8801     : --------------------------------------------------------------------- :    -
Aovo-DRB*W2901     : --------------------------------------------------------------------- :    -
Aona-DRB*W2908     : --------------------------------------------------------------------- :    -
Aona-DRB*W2910     : --------------------------------------------------------------------- :    -
Aovo-DRB*W3001     : --------------------------------------------------------------------- :    -
Aona-DRB*W3002     : --------------------------------------------------------------------- :    -
Aovo-DRB*W9201     : --------------------------------------------------------------------- :    -
Aovo-DRB*W9202     : --------------------------------------------------------------------- :    -
Aona-DRB*W9101     : --------------------------------------------------------------------- :    -
Aovo-DRB*W9101     : --------------------------------------------------------------------- :    -
Aovo-DRB*W9102     : --------------------------------------------------------------------- :    -
Aovo-DRB*W9001     : --------------------------------------------------------------------- :    -
Aovo-DRB*W4501     : --------------------------------------------------------------------- :    -
Aovo-DRB*W9301     : --------------------------------------------------------------------- :    -

                     0         *        780         *        800         *        820

Aona-DRB1*0328GB   : GAGAGACTGAGAGAGACTGAGAGAAAGACTGAGAGAGAGACTGAGAGAGGCTGAGAGACTGAGGGAGA- :  735
Aona-DRB1*0329GA   : GAGAGACTGAGAGAGACTAAGAGA--GACTGAGGGAGA--CTGAGAGAGACTGAAAGACTGAGAGAGAG :  812
Aona-DRB1*031701GA : GAGAGACTGAGAGAGACTAAGAGA--GACTGAGGGAGA--CTGAGAGAGACTGAAAGACTGAGAGAGAG :  812
Aovo-DRB1*0304GA   : ----------------------GA----------------CTGAGAGAGACTGAGAGACTGAGAGAAA- :  599
Aovo-DRB1*0305GA   : ----------GAGAGACTGAGAGA--TACTTAGGGAGA--CTGAGAGAGACAGAGAGACTGAGAGAGA- :  651
Aovo-DRB1*0306GA   : ----------------------GA----------------CTGAGAGAGACTGAGAGACTGAGAGAAA- :  609
Aovo-DRB1*0307GA   : ----------------------GA----------------CTGAGAGAGACTGAGAGACTGAGAGAAA- :  613
Aona-DRB3*0615     : ----------------CTGAGAGA----------AAGA--CTGAGAGAGACTGAGAGACTGAGAGAAA- :  627
Aona-DRB3*0627     : ----------GAGAGACTGAGAGA--TACTTAGGGAGA--CTGAGAGAGACAGAGAGACTGAGAGAAAG :  668
Aona-DRB3*062501   : ----------------CTGAGAGA----------AAGA--CTGAGAGAGACTGAGAGACTGAGAGAAA- :  627
Aona-DRB3*0626     : ----------------CTGAGAGA----------AAGA--CTGAGAGAGACTGAGAGACTGAGAGAAA- :  625
Aona-DRB3*062502   : ----------------CTGAAAGA----------------CTGAGAGAGACTGAGAGACTGAGAGAAA- :  623
Aona-DRB3*0628     : ----------GAGAGACTGAGAGA--TACTTAGGGAGA--CTGAGAGAGACAGAGAGACTGAGAGAAAG :  664
Aovo-DRB3*0601     : ----------------CTGAGAGA----------AAGA--CTGAGAGAGACTGAGAGACTGAGAGAAA- :  626
Aona-DRB*W8901     : ---------------------------------------------------------------AAGAC- :  418
Aona-DRB*W1808     : -----------------------------------------------------------GACTGAGA-- :  487
Aona-DRB*W1806     : -----------------------------------------------------------GACTGAGA-- :  477
Aovo-DRB*W1801     : -----------------------------------------------------------GACTGAGA-- :  459
Aovo-DRB*W1802     : -----------------------------------------------------------GACTGAGA-- :  475
Aovo-DRB*W1803     : -----------------------------------------------------------GACTGAGA-- :  463
Aovo-DRB*W8801     : -----------------------------------------------------------CTGAAAGAC- :  462
Aovo-DRB*W2901     : ---------------------------------------------------------------AAGAC- :  418
Aona-DRB*W2908     : ---------------------------------------------------------------AAGAC- :  418
Aona-DRB*W2910     : ---------------------------------------------------------------AAGAC- :  418
Aovo-DRB*W3001     : ---------------------------------------------------------------GAGAG- :  411
Aona-DRB*W3002     : ---------------------------------------------------------------GAG--- :  413
Aovo-DRB*W9201     : ---------------------------------------------------------------GAG--- :  409
Aovo-DRB*W9202     : ---------------------------------------------------------------GAG--- :  409
Aona-DRB*W9101     : --------------------------------------------------------------------- :    -
Aovo-DRB*W9101     : --------------------------------------------------------------------- :    -
Aovo-DRB*W9102     : --------------------------------------------------------------------- :    -
Aovo-DRB*W9001     : --------------------------------------------------------------------- :    -
Aovo-DRB*W4501     : --------------------------------------------------------------------- :    -
Aovo-DRB*W9301     : --------------------------------------------------------------------- :    -
```

```
                      *       840         *       860         *       880         *

Aona-DRB1*0328GB   : ----------------CAGAGAATGAGAGAGA---GACTGAGAGA---TAAGACAGAGACTGAGAGAGA :  782
Aona-DRB1*0329GA   : ACTGAGAGAGACAAGACAGAGACTGAGAGAGATGAGACAGAGAGAGACTGAGAGAAAGACTGAGAGACA :  881
Aona-DRB1*031701GA : ACTGAGAGAGACAAGACAGAGACTGAGAGAGATGAGACAGAGAGAGACTGAGAGAAAGACTGAGAGACA :  881
Aovo-DRB1*0304GA   : --------------------------------------------------------------------- :    -
Aovo-DRB1*0305GA   : -----------------------------------------------CTGAGAGAAAGACTGAGAGA-- :  671
Aovo-DRB1*0306GA   : --------------------------------------------------------------------- :    -
Aovo-DRB1*0307GA   : --------------------------------------------------------------------- :    -
Aona-DRB3*0615     : --------------------------------------------------------------------- :    -
Aona-DRB3*0627     : ACT------------------------------------------------GAGAGAGACTGAGAGACT :  689
Aona-DRB3*062501   : --------------------------------------------------------------------- :    -
Aona-DRB3*0626     : --------------------------------------------------------------------- :    -
Aona-DRB3*062502   : --------------------------------------------------------------------- :    -
Aona-DRB3*0628     : ACT------------------------------------------------GAGAGAGACTGAGAGACT :  685
Aovo-DRB3*0601     : --------------------------------------------------------------------- :    -
Aona-DRB*W8901     : --------------------------------------------------------------------- :    -
Aona-DRB*W1808     : --------------------------------------------------------------------- :    -
Aona-DRB*W1806     : --------------------------------------------------------------------- :    -
Aovo-DRB*W1801     : --------------------------------------------------------------------- :    -
Aovo-DRB*W1802     : --------------------------------------------------------------------- :    -
Aovo-DRB*W1803     : --------------------------------------------------------------------- :    -
Aovo-DRB*W8801     : --------------------------------------------------------------------- :    -
Aovo-DRB*W2901     : --------------------------------------------------------------------- :    -
Aona-DRB*W2908     : --------------------------------------------------------------------- :    -
Aona-DRB*W2910     : --------------------------------------------------------------------- :    -
Aovo-DRB*W3001     : --------------------------------------------------------------------- :    -
Aona-DRB*W3002     : --------------------------------------------------------------------- :    -
Aovo-DRB*W9201     : --------------------------------------------------------------------- :    -
Aovo-DRB*W9202     : --------------------------------------------------------------------- :    -
Aona-DRB*W9101     : --------------------------------------------------------------------- :    -
Aovo-DRB*W9101     : --------------------------------------------------------------------- :    -
Aovo-DRB*W9102     : --------------------------------------------------------------------- :    -
Aovo-DRB*W9001     : --------------------------------------------------------------------- :    -
Aovo-DRB*W4501     : --------------------------------------------------------------------- :    -
Aovo-DRB*W9301     : --------------------------------------------------------------------- :    -

                     900         *       920         *       940         *       960
                                                         ga ag             g        agagac
Aona-DRB1*0328GB   : T-GAGACAGAGAGA----GACTGA--GAGAAAGACTGAGAGACAGAGA----CTGAGAGACTGAGAGA- :  839
Aona-DRB1*0329GA   : --GAGACTGAGAGACTGAGACTGAGAGAAATAGACTGAGAGACTGAGAGACACTGAGAGACTGAGAGAC :  948
Aona-DRB1*031701GA : --GAGACTGAGAGACTGAGACTGAGAGAAATAGACTGAGAGACTGAGAGACACTGAGAGACTGAGAGAC :  948
Aovo-DRB1*0304GA   : ------CAGAGA----------------------CTGAGAGACTGAGAGACACTGAGAGACTGAGAGAC :  640
Aovo-DRB1*0305GA   : ----GACTGAGAGA------------AACAGGGACTGAGAGACTGAGAGACACTGAGAGACTGAGAGAC :  724
Aovo-DRB1*0306GA   : ------CAGAGA----------------------CTGAGAGACTGAGAGACACTGAGAGACTGAGAGAC :  650
Aovo-DRB1*0307GA   : ------CAGAGA----------------------CTGAGAGACTGAGAGACACTGAGAGACTGAGAGAC :  654
Aona-DRB3*0615     : ------CAGAGA----------------------CTGAGAGACTGAGAGACACTGAGAGACTGAGAGAC :  668
Aona-DRB3*0627     : GAGAGACTGAGAGA------------AACAGAGACTGAGAGACTGAGAGACACTGAGAGACTGAGAGAC :  746
Aona-DRB3*062501   : ------CAGAGA----------------------CTGAGAGACTGAGAGACACTGAGAGACTGAGAGAC :  668
Aona-DRB3*0626     : ------CAGAGA----------------------CTGAGAGACTGAGAGACACTGAGAGACTGAGAGAC :  666
Aona-DRB3*062502   : ------CAGAGA----------------------CTGAGAGACTGAGAGACACTGAGAGACTGAGAGAC :  664
Aona-DRB3*0628     : GAGAGACTGAGAGA------------AACAGAGACTGAGAGACTGAGAGACACTGAGAGACTGAGAGAC :  742
Aovo-DRB3*0601     : ------CAGAGA----------------------CTGAGAGACTGAGAGACACTGAGAGACTGAGAGAC :  667
Aona-DRB*W8901     : ----------------------------------GAGACAG------------AG--------AGAAAC :  433
Aona-DRB*W1808     : ----------------------------------GAGGCTG------------AGAGACTCGGAGAGAC :  510
Aona-DRB*W1806     : ----------------------------------GAGACTG------------AGAGACTCGGAGAGAC :  500
Aovo-DRB*W1801     : ----------------------------------GAGACTG------------AGAGACTCGGAGAGAC :  482
Aovo-DRB*W1802     : ----------------------------------GAGACTG------------AGAGACTCGGAGAGAC :  498
Aovo-DRB*W1803     : ----------------------------------GAGACTG------------AGAGACTCGGAGAGAC :  486
Aovo-DRB*W8801     : ----------------------------------GAGACAG------------AG--------AGAGAC :  477
Aovo-DRB*W2901     : ----------------------------------GAGACAG------------AG--------AGAGAC :  433
Aona-DRB*W2908     : ----------------------------------GAGACAG------------AG--------AGAAAC :  433
Aona-DRB*W2910     : ----------------------------------GAGACAG------------AG--------AGAAAC :  433
Aovo-DRB*W3001     : ----------------------------------GAGACAG------------AG--------AGAGAC :  426
Aona-DRB*W3002     : -----------------------------------AGAGAG------------AGT-------GGAGAC :  428
Aovo-DRB*W9201     : ----------------------------------GAGACAG------------AG--------AGAGAC :  424
Aovo-DRB*W9202     : ----------------------------------GAGACAG------------AG--------AGAGAC :  424
Aona-DRB*W9101     : ----------------------------------GACTGAG------------AGAGA----GAGACTG :  392
Aovo-DRB*W9101     : ----------------------------------GACTGAG------------AGAGA----GAGACTG :  368
Aovo-DRB*W9102     : ----------------------------------GACTGAG------------AGAGA----GAGACTG :  392
Aovo-DRB*W9001     : ----------------------------------GAATGAG------------AGAGA----CTGAGAG :  374
Aovo-DRB*W4501     : -----------------------------------AGACAG------------AG--------AGAGAC :  413
Aovo-DRB*W9301     : --------------------------------------------------------------------- :    -
```

```
                        *       980         *      1000         *      1020         *
                       ag  aga a                             ActGA agAga   agA A
Aona-DRB1*0328GB   : ---GACAGAGACTGAGAGACTGAGAGA----CTGAGAGACACAGATAGAGATTGAGAGACTGAGAGAGA :  901
Aona-DRB1*0329GA   : ACAGATAGAGATTGAGAGACTGAGAGAGAGACTGAGAGACACTGATAGAGACTGAGAGAGAGACTGAGA : 1017
Aona-DRB1*031701GA : ACAGATAGAGATTGAGAGACTGAGAGAGAGACTGAGAGACACTGATAGAGACTGAGAGAGAGACTGAGA : 1017
Aovo-DRB1*0304GA   : ACAGATAGAGATTGAGAGACTGAGAGA------------CACTGATAGAGACTGAGAGA--GACTGAGA :  695
Aovo-DRB1*0305GA   : ACAGATAGAAATTGAGAGACTGAGAGA------------CACTGATAGAGACTGAGAGAGAGACTGAGA :  781
Aovo-DRB1*0306GA   : ACAGATAGAGATTGAGAGACTGAGAGA------------CACTGATAGAGACTGAGAGA--GACTGAGA :  705
Aovo-DRB1*0307GA   : ACAGATAGAGATTGAGAGACTGAGAGA------------CACTGATAGAGACTGAGAGA--GACTGAGA :  709
Aona-DRB3*0615     : ACAGATAGAGATTGAGAGACTGAGAGAGAGACTGAGAGACACTGATAGAGACTGAGAGAGAGACTGAGA :  737
Aona-DRB3*0627     : ACAGATAGAGATTGAGAGACTGAGAGA------------CACTGATAGAGACTGAGAGAGAGACTGAGA :  803
Aona-DRB3*062501   : ACAGATAGAGATTGAGAGACTGAGAGAGAGACTGAGAGACACTGATAGAGACTGAGAGAGAGACTGAGA :  737
Aona-DRB3*0626     : ACAGATAGAGATTGAGAGACTGAGAGAGAGACTGAGAGACACTGATAGAGACTGAGAGAGAGACTGAGA :  735
Aona-DRB3*062502   : ACAGATAGAGATTGAGAGACTGAGAGAGAGACTGAGAGACACTGATAGAGACTGAGAGAGAGA------ :  727
Aona-DRB3*0628     : ACAGATAGAGATTGAGAGACTGAGAGA------------CACTGATAGAGACTGAGAGAGAGACTGAGA :  799
Aovo-DRB3*0601     : ACAGATAGAGATTGAGAGACTGAGAGAGAGACTGAGAGACACTGATAGAGACTGAGAGAGAGACTGAGA :  736
Aona-DRB*W8901     : TGAG--AGAAACAT--------------------AGA--GACTGAGAGAGAG--ACACA---------- :  466
Aona-DRB*W1808     : TGAGCGAGAGACTG--------------------AGATAGACTGAGAGAAACTGAGAGA---------- :  549
Aona-DRB*W1806     : TGAGCGAGAGACTG--------------------AGATAGACTGAGAGAAACTGAGAGA---------- :  539
Aovo-DRB*W1801     : TGAGCGAGAGACTG--------------------AGATAGACTGAGAGAAACTGAGAGA---------- :  521
Aovo-DRB*W1802     : TGAGCGAGAGACTG--------------------AGATAGACTGAGAGAAAC--AGAGA---------- :  535
Aovo-DRB*W1803     : TGAGCGAGAGACTG--------------------AGATAGACTGAGAGAAACTGAGAGA---------- :  525
Aovo-DRB*W8801     : TGAG--AGAAACAG--------------------AGA--GACTGAGAGAGGG--ACACA---------- :  510
Aovo-DRB*W2901     : TGAG--AGAAACAT--------------------AGA--GACTGAGAGAGAG--ACACA---------- :  466
Aona-DRB*W2908     : TGAG--AGAAACAT--------------------AGA--GACTGAGAGAGAG--ACACA---------- :  466
Aona-DRB*W2910     : TGAG--AGAAACAT--------------------AGA--GACTGAGAGAGAG--ACACA---------- :  466
Aovo-DRB*W3001     : --AG--AGAGACTG--------------------AGA--GACTGAGAAAGAC--AGAGA---------- :  457
Aona-DRB*W3002     : AGAG--AGAGACAG--------------------AGA--GACTGAGAAAGAC--AGAGA---------- :  461
Aovo-DRB*W9201     : --AG--AGAGACTG--------------------AGA--GACTGAGAAAGAC--AGAGA---------- :  455
Aovo-DRB*W9202     : --AG--AGAGACTG--------------------AGA--GACTGAGAAAGAC--AGAGA---------- :  455
Aona-DRB*W9101     : AAAGACGAGACA-------------------------GAGAGAGACTGAGAGAGGGACA---------- :  426
Aovo-DRB*W9101     : AAAGACGAGACA-------------------------GAGAGAGACTGAGAGAGGGACA---------- :  402
Aovo-DRB*W9102     : AAAGACGAGACA-------------------------GAGAGAGACTGAGAGAGGGACA---------- :  426
Aovo-DRB*W9001     : AGACTGAGAGAT----------------------------AGAGACTGAGAGACAGACA---------- :  405
Aovo-DRB*W4501     : --AG--AGAGGCTG--------------------AGA--GACTGAGAAAGAC--AGAGA---------- :  444
Aovo-DRB*W9301     : ------------------------------------AGAGACTGAGAGAGAGAGAGAGA---------- :  379

                      1040         *      1060         *      1080         *      1100
                         ag  agaGAGa AgaG ga                                GaGagaGc
Aona-DRB1*0328GB   : GACTGGGGGGG-GAGAGAGAGAGAGAGAGAGAGA--------------------CTGAGAGAGCACGTG :  949
Aona-DRB1*0329GA   : GACTGAGAGAGAGAGAGAGAGAGAGAGAGAGAGAGAGAGAGAGAGAGAGAGA----CTGAGAGAGCGCGTG : 1082
Aona-DRB1*031701GA : GACTGAGAGAGAGAGAGAGAGAGAGAGAGAGAGAGAGAGAGAGAGAGAGAGAGACTGAGAGAGCGCGTG : 1086
Aovo-DRB1*0304GA   : GACTGAGAGAGAGAGAGAGAGAGAGAAA--------------------------CTGAGAGAGCACTTG :  738
Aovo-DRB1*0305GA   : GACTGAGAGACAGAGAGAGAGAGAGAGAGAAA----------------------CTGAGAGAGCACTTG :  828
Aovo-DRB1*0306GA   : GACTGAGAGAGAGAGAGAGAGAGAGAGAGAAA----------------------CTGAGAGAGCACTTG :  752
Aovo-DRB1*0307GA   : GACTGAGAGAGAGAGAGAGAGAGAGAGAGAAA----------------------CTGAGAGAGCACTTG :  756
Aona-DRB3*0615     : GACTGAGAGAGAGAGAGAGAGAGAGAGAGAGAGA--------------------CTGAGAGAGCGCTTG :  786
Aona-DRB3*0627     : GACTGAGAGAGAGAGAGAGAGAGAAA----------------------------CTGAGAGAGCACTTG :  844
Aona-DRB3*062501   : GACTGAGAGAGAGAGAGAGAGAGAGAGAGAGAGA--------------------CTGAGAGAGCGCTTG :  786
Aona-DRB3*0626     : GACTGAGAGAGAGAGAGAGAGAGAGAGAGAGAGA--------------------CTGAGAGAGCGCTTG :  784
Aona-DRB3*062502   : ------GAGAGAGAGAGAGAGAGAGAGA--------------------------CTGAGAGAGCGCTTG :  764
Aona-DRB3*0628     : GACTGAGAGAGAGAGAGAGAGAGAGAAA--------------------------CTGAGAGAGCACTTG :  842
Aovo-DRB3*0601     : GACTGAGAGAGAGAGAGAGAGAGAGAGAGAGAGA--------------------CTGAGAGAGCGCTTG :  785
Aona-DRB*W8901     : ----GAG--AGAGAGACAGAGCGA--------------------------------GAGAGAGCG---- :  493
Aona-DRB*W1808     : ----TACTGAGAGAGAGAGAGCGC--------------------------------GCGACAGCG---- :  578
Aona-DRB*W1806     : ----TACTGAGAGAGAGAGAGCGC--------------------------------GCGACAGCG---- :  568
Aovo-DRB*W1801     : ----TACTGAGAGAGAGAGAGCGC--------------------------------GCGACAGCG---- :  550
Aovo-DRB*W1802     : ----TACTGAGAGAGAGAGAGCGC--------------------------------GCGACAGCG---- :  564
Aovo-DRB*W1803     : ----TACTGAGAGAGAGAGAGCGC--------------------------------GCGACAGCG---- :  554
Aovo-DRB*W8801     : ----GAG--AGAGAGACAGAGCGA--------------------------------GAGAGAGCG---- :  537
Aovo-DRB*W2901     : ----GAG--AGAGAGACAGAGCGA--------------------------------GAGAGAGCG---- :  493
Aona-DRB*W2908     : ----GAG--AGAGAGACAGAGCGA--------------------------------GAGAGAGCG---- :  493
Aona-DRB*W2910     : ----GAG--AGAGAGACAGAGCGA--------------------------------GAGAGAGCG---- :  493
Aovo-DRB*W3001     : ----GAGCCAGAGAGCCAGAGCCA--------------------------------GAGAGAGCA---- :  486
Aona-DRB*W3002     : ----GAGCCAGAGAGCCAGAGCCA--------------------------------GAGAGAGCA---- :  490
Aovo-DRB*W9201     : ----GAGCCAGAGAGCCAGAGCCA--------------------------------GAGAGAGCA---- :  484
Aovo-DRB*W9202     : ----GAGCCAGAGAGCCAGAGCCA--------------------------------GAGAGAGCA---- :  484
Aona-DRB*W9101     : ----CAGAGAGAGAGACAGAGCGA--------------------------------GAGAGAGCG---- :  455
Aovo-DRB*W9101     : ----CAGAGAGAGAGACAGAGCGA--------------------------------GAGAGAGCG---- :  431
Aovo-DRB*W9102     : ----CAGAGAGAGAGACAGAGCGA--------------------------------GAGAGAGCG---- :  455
Aovo-DRB*W9001     : ----CAGAGAGAGAGACAGAGCGA--------------------------------GAGAGAGCG---- :  434
Aovo-DRB*W4501     : ----GAGCCAGAGAGCCAGAGCCA--------------------------------GAGAGAGCA---- :  473
Aovo-DRB*W9301     : ----GAGAGAGAGAGAGACTGAGA--------------------------------GAGCGCGTG---- :  408
```

```
                          *      1120         *      1140         *      1160         *
                       C aTCTGTGAG  TT AGaATCCTcTc ATCCTGAGCagGGAGcTCtGaGGGCACAggTGTgTGTgt
Aona-DRB1*0328GB    : CCATCTGTGAGCATTCAGAATCCTCTCCATCCTGAGCAGGGAGCTCTGAGGGCACAGGTGTGTGTGT-- : 1016
Aona-DRB1*0329GA    : CCATCTGTGAGCATTCAGAATCCTGTCCATCCTGAGCAGGGAGCTCTGGGGGCACAGGTGTGTGTAT-- : 1149
Aona-DRB1*031701GA  : CCATCTGTGAGCATTCAGAATCCTGTCCATCCTGAGCAGGGAGCTCTGAGGGCACAGGTGTTTGTAT-- : 1153
Aovo-DRB1*0304GA    : CAATCTGTGAGCATTCAGAATCCTCTCCATCCTGAGCAGGGAGCTCTGAGGGCACAGGTGTGTGTGT-- :  805
Aovo-DRB1*0305GA    : CAATCTGTGAGCATTCAGAATCCTCTCCATCCTGAGCAGGGAGCTCTGAGGGCACAGGTGTGTGTGT-- :  895
Aovo-DRB1*0306GA    : CAATCTGTGAGCATTCAGAATCCTCTCCATCCTGAGCAGGGAGCTCTGAGGGCACAGGTGTGTGTGT-- :  819
Aovo-DRB1*0307GA    : CAATCTGTGAGCATTCAGAATCCTCTCCATCCTGAGCAGGGAGCTCTGAGGGCACAGGTGTGTGTGT-- :  823
Aona-DRB3*0615      : CAGTCTGTGAGCATTCAGAATCCTCTCCATCCTGAGCAGGGAGCTCTGAGGGCACAGGTGTGTGT---- :  851
Aona-DRB3*0627      : CCATCTGTGAGCATTCAGAATCCTCTCCATCCTGAGCAGGGAGCTCTGAGGGCACAGGTGTGTGTGT-- :  911
Aona-DRB3*062501    : CAGTCTGTGAGCATTCAGAATCCTCTCCATCCTGAGCAGGGAGCTCTGAGGGCACAGGTGTGTGT---- :  851
Aona-DRB3*0626      : CAGTCTGTGAGCATTCAGAATCCTCTCCATCCTGAGCAGGGAGCTCTGAGGGCACAGGTGTGTGT---- :  849
Aona-DRB3*062502    : CAGTCTGTGAGCATTCAGAATCCTCTCCATCCTGAGCAGGGAGCTCTGAGGGCACAGGTGTGTGT---- :  829
Aona-DRB3*0628      : CCATCTGTGAGCATTCAGAATCCTCTCCATCCTGAGCAGGGAGCTCTGAGGGCACAGGTGTGTGTGT-- :  909
Aovo-DRB3*0601      : CAGTCTGTGAGCATTCAGAATCCTCTCCATCCTGAGCAGGGAGCTCTGAGGGCACAGGTGTGTGT---- :  850
Aona-DRB*W8901      : CCATCTGTGAGAGTTCAGTATCCTCTCAATCCTGAGCAGGGAGCTCTGAGGGCACAGGTGTGTGTGT-- :  560
Aona-DRB*W1808      : CCATCTGTGAGCGTTTAGAATCCTCTCAATCCTGAGCGGGGAGCTCTGAGGGCACAAGTGTGTGTGT-- :  645
Aona-DRB*W1806      : CCATCTGTGAGCGTTTAGAATCCTCTCAATCCTGAGCAGGGAGCTCCGAGGGCACAAGTGTGTGTGT-- :  635
Aovo-DRB*W1801      : CCATCTGTGAGCGTTTAGAATCCTCTCAATCCTGAGCAGGGAGCTCTGAGGGCACAGGTGTGTGTGTGT :  619
Aovo-DRB*W1802      : CCATCTGTGAGAGTTTAGAATCCTCTCAATCCTGAGCAGGGAGCTCTGAGGGCACAGGTGTGTGTGT-- :  631
Aovo-DRB*W1803      : CCATCTGTGAGCGTTTAGAATCCTCTCAATCCTGAGCAGGGAGCTCTGAGGGCACAGGTGTGTGTGT-- :  621
Aovo-DRB*W8801      : CCATCTGTGAGAGTTTAGAATCCTCTCAATCCTGAGCAAGGAGTTCTGAGGGCACAGATGTGTGTGT-- :  604
Aovo-DRB*W2901      : CCATCTGTGAGAGTTCAGTATCCTCTCAATCCTGAGCAGGGAGCTCTGAGGGCACAGGTGTGTGTGT-- :  560
Aona-DRB*W2908      : CCATCTGTGAGAGTTCAGTATCCTCTCAATCCTGAGCAGGGAGCTCTGAGGGCACAGGTGTGTGTGT-- :  560
Aona-DRB*W2910      : CCATCTGTGAGAGTTCAGTATCCTCTCAATCCTGAGCAGGGAGCTCTGAGGGCACAGGTGTGTGTGT-- :  560
Aovo-DRB*W3001      : CCATCTGTGAGAGTTTAGAATCCTCTAAATCCTGAGCAGGGAGCTCTGAGGGCACAGGTGTGTGTGT-- :  553
Aona-DRB*W3002      : CCATCTGTGAGAGTTTAGAATCCTCTAAATCCTGAGCAGGGAGCTCTGAGGGCACAGGTGTGTGTGT-- :  557
Aovo-DRB*W9201      : CCATCTGTGAGAGTTTAGAATCCTCTAAATCCTGAGCAGGGAGCTCTGAGGGCACAGGTGTGTGTGT-- :  551
Aovo-DRB*W9202      : CCATCTGTGAGAGTTTAGAATCCTCTAAATCCTGAGCAGGGAGCTCTGAGGGCACAGGTGTGTGTGT-- :  551
Aona-DRB*W9101      : CCATCTGTGAGAGTTTAGAATCCTCTCAATCCTGAGCAAGGAGTTCTGAGGGCACAGATGTGTGTGT-- :  522
Aovo-DRB*W9101      : CCATCTGTGAGAGTTTAGAATCCTCTCAATCCTGAGCAAGGAGTTCTGAGGGCACAGATGTGTGTGT-- :  498
Aovo-DRB*W9102      : CCATCTGTGAGAGTTTAGAATCCTCTCAATCCTGAGCAAGGAGTTCTGAGGGCACAGATGTGTGTGT-- :  522
Aovo-DRB*W9001      : CCATCTGTGAGAGTTCAGTATCCTCTCAATCCTGAGCAAGGAGCTCTGAGGGCACAGGTGTGTGTGT-- :  501
Aovo-DRB*W4501      : CCATCTGTGAGAGTTTAGAATCCTCTAAATCCTGAGCAGGGAGCTCTGAGGGCACAGGTGTGTGTGT-- :  540
Aovo-DRB*W9301      : CCATCTGTGAGCATTCAGTATCCTCTCAATCCTGAGCAGGGAGCTCTGAGGGCACAGATGTGTGTGT-- :  475

                       1180         *      1200         *      1220         *      1240
                       AGAGTGTGGATTTGTGTG G GGCTGTTGTGgGagGgGAgGCAGGAGGGGGCTTCTTC TA CCTTGGA
Aona-DRB1*0328GB    : AGAGTGTGGATTTGTGTGTGTGGCTGTTGTGGGAGGGGAGGCAGGAGGGGGCTTCTTCCTACCCTTGGA : 1085
Aona-DRB1*0329GA    : AGAGTGTGGATTTGTGTGTGAGGCTGTTGTGGGAGGGGAGGCAGGAGGGGGCTTCTTCCTATCCTTGGA : 1218
Aona-DRB1*031701GA  : AGAGTGTGGATTTGTGTGTGAGGCTGTTGTGGGAGGGGAGGCAGGAGGGGGCTTCTTCCTATCCTTGGA : 1222
Aovo-DRB1*0304GA    : AGAGTGTGGATTTGTGTGTGAGGCTGTTGTGGGAGGGGAGGCAGGAGGGGGCTTCTTCCTATCCTTGGA :  874
Aovo-DRB1*0305GA    : AGAGTGTGGATTTGTGTGTGAGGCTGTTGTGGGAGGGGAGGCAGGAGGGGGCTTCTTCCTATCCTTGGA :  964
Aovo-DRB1*0306GA    : AGAGTGTGGATTTGTGTGTGAGGCTGTTGTGGGAGGGGAGGCAGGAGGGGGCTTCTTCCTATCCTTGGA :  888
Aovo-DRB1*0307GA    : AGAGTGTGGATTTGTGTGTGAGGCTGTTGTGGGAGGGGAGGCAGGAGGGGGCTTCTTCCTATCCTTGGA :  892
Aona-DRB3*0615      : AGAGTGTGGATTTGTGTGTGAGGCTGTTGTGGGAGGGGAGGCAGGAGGGGGCTTCTTCCTATCCTTGGA :  920
Aona-DRB3*0627      : AGAGTGTGGATTTGTGTGTGAGGCTGTTGTGGGAGGGGAGGCAGGAGGGGGCTTCTTCCTATCCTTGGA :  980
Aona-DRB3*062501    : AGAGTGTGGATTTGTGTGTGAGGCTGTTGTGGGAGGGGAGGCAGGAGGGGGCTTCTTCCTATCCTTGGA :  920
Aona-DRB3*0626      : AGAGTGTGGATTTGTGTGTGAGGCTGTTGTGGGAGGGGAGGCAGGAGGGGGCTTCTTCCTATCCTTGGA :  918
Aona-DRB3*062502    : AGAGTGTGGATTTGTGTGTGAGGCTGTTGTGGGAGGGGAGGCAGGAGGGGGCTTCTTCCTATCCTTGGA :  898
Aona-DRB3*0628      : AGAGTGTGGATTTGTGTGTGAGGCTGTTGTGGGAGGGGAGGCAGGAGGGGGCTTCTTCCTATCCTTGGA :  978
Aovo-DRB3*0601      : AGAGTGTGGATTTGTGTGTGAGGCTGTTGTGGGAGGGGAGGCAGGAGGGGGCTTCTTCCTATCCTTGGA :  919
Aona-DRB*W8901      : AGAGTGTGGATTTGTGTGTGTGGCTGTTGTGAG-AGGGAGGCAGGAGGGGGCTTCTTCTTACCCTTGGA :  628
Aona-DRB*W1808      : AGAGTGTGGATTTGTGTGTGTGGCTGTTGTGGGAGGGGAAGCAGGAGGGGGCTTCTTCTTATCCTTGGA :  714
Aona-DRB*W1806      : AGAGTGTGGATTTGTGTGTGTGGCTGTTGTGGGAGGGGAAGCAGGAGGGGGCTTCTTCTTATCCTTGGA :  704
Aovo-DRB*W1801      : AGAGTGTGGATTTGTGTGTGTGGCTGTTGTGGGAGGGGAAGCAGGAGGGGGCTTCTTCTTATCCTTGGA :  688
Aovo-DRB*W1802      : AGAGTGTGGATTTGTGTGTGAGGCTGTTGTGGGAGGGGAGGCAGGAGGGGGCTTCTTCCTATCCTTGGA :  700
Aovo-DRB*W1803      : AGAGTGTGGATTTGTGTGTGTGGCTGTTGTGGGAGGGGAAGCAGGAGGGGGCTTCTTCTTATCCTTGGA :  690
Aovo-DRB*W8801      : AGAGTGTGGATTTGTGTGCGTGGCTGTTGTGGGAGGGGAGGCAGGAGGGGGCTTCTTCTTACCCTTGGA :  673
Aovo-DRB*W2901      : AGAGTGTGGATTTGTGTGTGTGGCTGTTGTGAGAGGGGAGGCAGGAGGGGGCTTCTTCTTACCCTTGGA :  629
Aona-DRB*W2908      : AGAGTGTGGATTTGTGTGTGTGGCTGTTGTGAGAGGGGAGGCAGGAGGGGGCTTCTTCTTACCCTTGGA :  629
Aona-DRB*W2910      : AGAGTGTGGATTTGTGTGTGTGGCTGTTGTGAGAGGGGAGGCAGGAGGGGGCTTCTTCTTACCCTTGGA :  629
Aovo-DRB*W3001      : AGAGTGTGGATTTGTGTGAGAGGCTGTTGTGGGAGGAGAGGCAGGAGGGGGCTTCTTCCTATCCTTGGA :  622
Aona-DRB*W3002      : AGAGTGTGGATTTGTGTGAGAGGCTGTTGTGGGAGGGGAGGCAGGAGGGGGCTTCTTCCTATCCTTGGA :  626
Aovo-DRB*W9201      : AGAGTGTGGATTTGTGTGAGAGGCTGTTGTGGGAGGAGAGGCAGGAGGGGGCTTCTTCTTATCCTTGGA :  620
Aovo-DRB*W9202      : AGAGTGTGGATTTGTGTGAGAGGCTGTTGTGGGAGGAGAGGCAGGAGGGGGCTTCTTCTTATCCTTGGA :  620
Aona-DRB*W9101      : AGAGTGTGGATTTGTGTGCGTGGCTGTTGTGGGAGGGGAGGCAGGAGGGGGCTTCTTCTTACCCTTGGA :  591
Aovo-DRB*W9101      : AGAGTGTGGATTTGTGTGCGTGGCTGTTGTGGGAGGGGAGGCAGGAGGGGGCTTCTTCTTACCCTTGGA :  567
Aovo-DRB*W9102      : AGAGTGTGGATTTGTGTGCGTGGCTGTTGTGGGAGGGGAGGCAGGAGGGGGCTTCTTCTTACCCTTGGA :  591
Aovo-DRB*W9001      : AGAGTGTGGATTTGTGTGTGTGGCTGTTGTGAGAGGGGAGGCAGGAGGGGGCTTCTTCTTACCCTTGGA :  570
Aovo-DRB*W4501      : AGAGTGTGGATTTGTGTGAGAGGCTGTTGTGGGAGGAGAGGCAGGAGGGGGCTTCTTCTTATCCTTGGA :  609
Aovo-DRB*W9301      : AGAGTGTGGATTTGTGTGTGAGGCTGTTGTGAGAGGCGAGGCAGGAGGGGGCTTCTTCTTATCCTTGGA :  544
```

```
                            *      1260         *      1280         *      1300         *
                     Ggcctct gtg  gagg gaca   gagg gg t cagggg tggaga ggaggagacct gattgtcc
Aona-DRB1*0328GB   : GGCCTCT-GTGGGGAGGTGACACAGGAGGTGGGTGCAGGGG-TGGAGAGGGAGGAGACCTGGATTGTCC : 1152
Aona-DRB1*0329GA   : GGCCTCT-GTGGGGAGGTGACACAGGAGGTGGGTGCAGGGG-TGGAGAGGGAGGAGACCTCGATTGTCC : 1285
Aona-DRB1*031701GA : GGCCTCT-GTGGGGAGGTGACACAGGAGGTGGGTGCAGGGG-TGGAGAGGGAGGAGACCTCGATTGTCC : 1289
Aovo-DRB1*0304GA   : GGCCTCT-GTGGGGAGGTGACACAGGAGGTGGGTGCAGGGG-TGGAGAGGGAGGAGACCTCGATTGTCC :  941
Aovo-DRB1*0305GA   : GGCCTCT-GTGGGGAGGTGACACAGGAGGTGGGTGCAGGGG-TGGAGAGGGAGGAGACCTCGATTGTCC : 1031
Aovo-DRB1*0306GA   : GGCCTCT-GTGGGGAGGTGACACAGGAGGTGGGTGCAGGGG-TGGAGAGGGAGGAGACCTCGATTGTCC :  955
Aovo-DRB1*0307GA   : GGCCTCT-GTGGGGAGGTGACACAGGAGGTGGGTGCAGGGG-TGGAGAGGGAGGAGACCTCGATTGTCC :  959
Aona-DRB3*0615     : GGCCTCT-GTGGGGAGGTGACACAGGAGGTGGGTGCAGGGG-TGGAGAGGGAGGAGACCTCGATTGTCC :  987
Aona-DRB3*0627     : GGCCTCT-GTGGGGAGGTGACACAGGAGGTGGGTGCAGGGG-TGGAGAGGGAGGAGACCTCGATTGTCC : 1047
Aona-DRB3*062501   : GGCCTCT-GTGGGGAGGTGACACAGGAGGTGGGTGCAGGGG-TGGAGAGGGAGGAGACCTCGATTGTCC :  987
Aona-DRB3*0626     : GGCCTCT-GTGGGGAGGTGACACAGGAGGTGGGTGCAGGGG-TGGAGAGGGAGGAGACCTCGATTGTCC :  985
Aona-DRB3*062502   : GGCCTCT-GTGGGGAGGTGACACAGGAGGTGGGTGCAGGGG-TGGAGAGGGAGGAGACCTCGATTGTCC :  965
Aona-DRB3*0628     : GGCCTCT-GTGGGGAGGTGACACAGGAGGTGGGTGCAGGGG-TGGAGAGGGAGGAGACCTCGATTGTCC : 1045
Aovo-DRB3*0601     : GGCCTCT-GTGGGGAGGTGACACAGGAGGTGGGTGCAGGGG-TGGAGAGGGAGGAGACCTCGATTGTCC :  986
Aona-DRB*W8901     : GGCCTCT-GTGAGGAGGTGACATGGGAGGCGGGTGCAGGGG-TGGAGAGGGAGGAGACCTGGATTGTCC :  695
Aona-DRB*W1808     : GGCCTCTTGTGTTGAGGGGACATGTGAGGTGGCTGCAGGGGCTGGAGAGGGAGGAGACCTCGATTGTCC :  783
Aona-DRB*W1806     : GGCCTCTTGTGTTGAGGGGACATGTGAGGTGGCTGCAGGGGCTGGAGAGGGAGGAGACCTCGATTGTCC :  773
Aovo-DRB*W1801     : G------------------------------------------------------------------- :  689
Aovo-DRB*W1802     : GGCCTCTTGTGTTGAGGGGACATGTGAGGTGGCTGCAGGGGCTGGAGAGGGAGGAGACCTCGATTGTCC :  769
Aovo-DRB*W1803     : GGCCTCTTGTGTTGAGGGGACATGTGAGGTGACTGCAGGGGCTGGAGAGGGAGGAGACCTCGATTGTCC :  759
Aovo-DRB*W8801     : GGCCTCT-GTGAGGAGGTGACATGGGAGGCGGGTGCAGGGG-TGGAGACGGAGGAGACCTGGATTGTCC :  740
Aovo-DRB*W2901     : GGCCTCT-GTGAGGAGGTGACATGGGAGGCGGGTGCAGGGG-TGGAGAGGGAGGAGACCTGGATTGTCC :  696
Aona-DRB*W2908     : GGCCTCT-GTGAGGAGGTGACATGGGAGGCGGGTGCAGGGG-TGGAGAGGGAGGAGACCTGGATTGTCC :  696
Aona-DRB*W2910     : GGCCTCT-GTGAGGAGGTGACATGGGAGGCGGGTGCAGGGG-TGGAGAGGGAGGAGACCTGGATTGTCC :  696
Aovo-DRB*W3001     : G------------------------------------------------------------------- :  623
Aona-DRB*W3002     : GGCCTCT-GTGGGGAGGTGACACAGGAGGTGGGTGCAGAGG-TGGGGAGGGAGGAGACCTCGTTTGTCA :  693
Aovo-DRB*W9201     : G------------------------------------------------------------------- :  621
Aovo-DRB*W9202     : G------------------------------------------------------------------- :  621
Aona-DRB*W9101     : GGCCTCT-GTGAGGAGGTGACATGGGAGGCGGGTACAGGGG-TGGAGACGGAGGAGACCTGGATTGTCC :  658
Aovo-DRB*W9101     : GGCCTCT-GTGAGGAGGTGACATGGGAGGCGGGTACAGGGG-TGGAGACGGAGGAGACCTGGATTGTCC :  634
Aovo-DRB*W9102     : GGCCTCT-GTGAGGAGGTGACATGGGAGGCGGGTACAGGGG-TGGAGACGGAGGAGACCTGGATTGTCC :  658
Aovo-DRB*W9001     : GGCCTCT-GTGAGGAGGTGACATGGGAGGCGGGTGCAGGGG-TGGAGAGGGAGGAGACCTGGATTGTCC :  637
Aovo-DRB*W4501     : G------------------------------------------------------------------- :  610
Aovo-DRB*W9301     : G------------------------------------------------------------------- :  545

                          1320         *      1340         *      1360         *
                     t ggtccttagagat caggaa  g aa   tga gtgtgtgtggctggggtgagggttta
Aona-DRB1*0328GB   : TTGGTCCTTAGAGATGCAGGAA-GGGAAATGTGAAGTGTGTGTGGCTGGGGTGAGGGTTTA : 1212
Aona-DRB1*0329GA   : TTGGTCCTTAGAGATGCAGGAA-GGGAAATGTGAAGTGTGTGTGGCTGGGGTGAGGGTTTA : 1345
Aona-DRB1*031701GA : TTGGTCCTTAGAGATGCAGGAA-GGGAAATGTGAAGTGTGTGTGGCTGGGGTGAGGGTTTA : 1349
Aovo-DRB1*0304GA   : TTGGTCCTTAGAGATGCAGGAA-GGGAAATGTGAAGTGTGTGTGGCTGGGGTGAGGGTTTA : 1001
Aovo-DRB1*0305GA   : TTGGTCCTTAGAGATGCAGGAA-GGGAAATGTGAAGTGTGTGTGGCTGGGGTGAGGGTTTA : 1091
Aovo-DRB1*0306GA   : TTGGTCCTTAGAGATGCAGGAA-GGGAAATGTGAAGTGTGTGTGGCTGGGGTGAGGGTTTA : 1015
Aovo-DRB1*0307GA   : TTGGTCCTTAGAGATGCAGGAA-GGGAAATGTGAAGTGTGTGTGGCTGGGGTGAGGGTTTA : 1019
Aona-DRB3*0615     : TTGGTCCTTAGAGATGCAGGAA-GGGAAATGTGAAGTGTGTGTGGCTGGGGTGAGGGTTTA : 1047
Aona-DRB3*0627     : TTGGTCCTTAGAGATGCAGGAA-GGGAAATGTGAAGTGTGTGTGGCTGGGGTGAGGGTTTA : 1107
Aona-DRB3*062501   : TTGGTCCTTAGAGATGCAGGAA-GGGAAATGTGAAGTGTGTGTGGCTGGGGTGAGGGTTTA : 1047
Aona-DRB3*0626     : TTGGTCCTTAGAGATGCAGGAA-GGGAAATGTGAAGTGTGTGTGGCTGGGGTGAGGGTTTA : 1045
Aona-DRB3*062502   : TTGGTCCTTAGAGATGCAGGAA-GGGAAATGTGAAGTGTGTGTGGCTGGGGTGAGGGTTTA : 1025
Aona-DRB3*0628     : TTGGTCCTTAGAGATGCAGGAA-GGGAAATGTGAAGTGTGTGTGGCTGGGGTGAGGGTTTA : 1105
Aovo-DRB3*0601     : TTGGTCCTTAGAGATGCAGGAA-GGGAAATGTGAAGTGTGTGTGGCTGGGGTGAGGGTTTA : 1046
Aona-DRB*W8901     : TGGGTCCTTAGAGATGCAGGAAGGGAAATG-TGAGGTGTGTGTGGCTGGGGTGAGGGTTTA :  755
Aona-DRB*W1808     : TGGGTCCTTAGAGATGCAGGAAGGGAAATG-TGAAGTGTGTGTGGCTGGGGTGAGGGTTTA :  843
Aona-DRB*W1806     : TGGGTCCTTAGAGATGCAGGAAGGGAAATG-TGAGGTGTGTGTGGCTGGGGTGAGGGTTTA :  833
Aovo-DRB*W1801     : ------------------------------------------------------------ :    -
Aovo-DRB*W1802     : TGGGTCCTTAGAGATGCAGGAAGGGAAATG-TGAGGTGTGTGTGGCTGGGGTGAGGGTTTA :  829
Aovo-DRB*W1803     : TTGGTCCTTAGAGATGCAGGAAGGGAAATG-TGAAGTGTGTGTGGCTGGGGTGAGGGTTTA :  819
Aovo-DRB*W8801     : TGGGTCCTTAGAGATGCAGGAATGGAAATG-TGAGGTGTGTGTGGCTGGGGTGAGGGTTTA :  800
Aovo-DRB*W2901     : TGGGTCCTTAGAGATGCAGGAAGGGAAATG-TGAGGTGTGTGTGGCTGGGGTGAGGGTTTA :  756
Aona-DRB*W2908     : TGGGTCCTTAGAGATGCAGGAAGGGAAATG-TGAGGTGTGTGTGGCTGGGGTGAGGGTTTA :  756
Aona-DRB*W2910     : TGGGTCCTTAGAGATGCAGGAAGGGACCTG-TGAGGTGTGTGTGGCTGGGGTGAGGGTTTA :  756
Aovo-DRB*W3001     : ------------------------------------------------------------ :    -
Aona-DRB*W3002     : TTGGTCCTTAGAGATGCAGGAATGGAAATG-TGAGGTGTGTGTGGCTGGGGTGAGGGTTTA :  753
Aovo-DRB*W9201     : ------------------------------------------------------------ :    -
Aovo-DRB*W9202     : ------------------------------------------------------------ :    -
Aona-DRB*W9101     : TGGGTCCTTAGAGATTCAGGAA-TGGAAATGTGAGGTGTGTGTGGCTGGGGTGAGGGTTTA :  718
Aovo-DRB*W9101     : TGGGTCCTTAGAGATTCAGGAA-TGGAAATGTGAGGTGTGTGTGGCTGGGGTGAGGGTTTA :  694
Aovo-DRB*W9102     : TGGGTCCTTAGAGATTCAGGAA-TGGAAATGTGAGGTGTGTGTGGCTGGGGTGAGGGTTTA :  718
Aovo-DRB*W9001     : TGGGTCCTTAGAGATGCAGGAA-GGGAAATGTGAGGTGTGTGTGGCTGGGGTGAGGGTTTA :  697
Aovo-DRB*W4501     : ------------------------------------------------------------ :    -
Aovo-DRB*W9301     : ------------------------------------------------------------ :    -
```
